# Identification and bioinformatic analysis of the membrane proteins of *synechocystis *sp. PCC 6803

**DOI:** 10.1186/1477-5956-7-11

**Published:** 2009-03-25

**Authors:** Yingchun Wang, Wu Xu, Parag R Chitnis

**Affiliations:** 1 Department of Biochemistry, Biophysics and Molecular Biology, Iowa State University, Ames, IA 50011, USA; 2 Department of Chemistry, University of Louisiana at Lafayette, Lafayette, LA 70504, USA; 3 Department of Pathology and Moores Cancer Center, University of California, San Diego, 9500 Gilman Dr., MC 0612, La Jolla, CA 92093, USA; 4 Division of Molecular & Cellular Biosciences (BIO/MCB), National Science Foundation, 4201 Wilson Blvd, Arlington, VA 22230, USA

## Abstract

**Background:**

The membranes of *Synechocystis *sp. PCC 6803 play a central role in photosynthesis, respiration and other important metabolic pathways. Comprehensive identification of the membrane proteins is of importance for a better understanding of the diverse functions of its unique membrane structures. Up to date, approximately 900 known or predicted membrane proteins, consisting 24.5% of *Synechocystis *sp. PCC 6803 proteome, have been indentified by large-scale proteomic studies.

**Results:**

To resolve more membrane proteins on 2-D gels for mass spectrometry identification, we separated integral proteins from membrane associated proteins and collected them as the integral and peripheral fractions, respectively. In total, 95 proteins in the peripheral fraction and 29 proteins in the integral fraction were identified, including the 5 unique proteins that were not identified by any previous studies. Bioinformatic analysis revealed that the identified proteins can be functionally classified into 14 distinct groups according to the cellular functions annotated by Cyanobase, including the two largest groups hypothetical and unknown, and photosynthesis and respiration. Homology analysis indicates that the identified membrane proteins are more conserved than the rest of the proteome.

**Conclusion:**

The proteins identified in this study combined with other published proteomic data provide the most comprehensive *Synechocystis *proteome catalog, which will serve as a useful reference for further detailed studies to address protein functions through both traditional gene-by-gene and systems biology approaches.

## Background

The membrane system of *Synechocystis *sp. PCC 6803 (thereafter referred as *Synechocystis*) is one of the best systems for performing functional membrane proteomic analysis because of its unique membrane organization. *Synechocystis*, a widely used model strain of gram-negative unicellular cyanobacterium for the studies of photosynthesis and other metabolic processes, has the outer and plasma membranes as well as an intracellular membrane system, called the thylakoid [1,2]. The outer and plasma membranes of *Synechocystis *contain important proteins involved in a variety of functions, such as nutrient uptake, secretion, and multidrug efflux pumps and energy transduction while the thylakoid membrane enriches photosynthetic and respiratory proteins [3-6]. A large-scale functional proteomic analysis can help to identify novel proteins involved in photosynthesis, respiration and other cellular processes to extend the current understanding of the fundamental signal transduction and metabolic pathways. In addition, the 3.57 Mb-genome of *Synechocystis *was completely sequenced [7] and a total of 3,673 genes including 3,168 genomic genes and 505 plasmid genes were annotated in Cyanobase , making it feasible for the large scale proteomic analysis. Furthermore, *Synechocystis *can be easily transformed and has a homologous recombination system, enabling the further functional study of proteins identified by proteome using reverse genetics approaches. Although multiple studies have been performed to catalog *Synechocystis *membrane proteome [3-6,8-10], it is still a challenge to identify all the membrane proteins due to their low abundance and low solubility.

Two-dimensional gel electrophoresis in conjunction with mass spectrometry has been widely used for global analysis of proteins. However, membrane proteins, especially integral membrane proteins, remain as difficult for 2-DE analysis due to their insolubility, high hydrophobicity, low abundance and/or aggregation during IEF [4,11-13]. Use of different combinations of strong nonionic detergents and chaotropes can increase the solubility of membrane proteins [14,15], whereas separation of membrane proteins into different compartments can help to enrich low-abundance membrane proteins [3-5,8]. In addition, protein prefractionation that reduces sample complexity and enriches low abundant proteins, combined with the use of stronger denaturing and reducing reagents compatible for 2-DE running conditions, could be an effective method to resolve membrane proteins on 2-D gels.

Previously, we identified 51 proteins from the membranes of *Synechocystis *by TCA/acetone precipitation and 2-DE, most of which are membrane associated proteins [6]. Here, we describe a different approach to separate and enrich integral proteins from membrane associated proteins using high concentration of urea. This approach has been reported to improve identification of integral proteins from the purified *Synechocystis *plasma membrane [4] and thylakoid membrane [5]. Proteins from each fraction were resolved by 2-DE with multiple pH ranges in the first dimension and were identified by matrix-assisted laser-desorption and ionization time-of-flight (MALDI-TOF) mass spectrometry. The results along with other published proteome data serve as a reference for further studies to address detailed functions of membrane proteins in a specific physiological context. Bioinformatic analysis suggested that the identified proteins are more evolutionally conserved than the rest of the proteins in the *Synechocystis *proteome. Functional classification of these proteins revealed that the identified membrane proteins are implicated in a wide spectrum of cellular processes, including the highly represented process, i.e., photosynthesis and respiration.

## Methods

### Growth of *Synechocystis *sp. PCC 6803 and preparation of membranes

The wild-type strain of *Synechocystis *was cultured in BG-11 medium [16] with 5.0 mM glucose under ~40 μmol·m^-2^·s^-1 ^light intensity at 30°C. For membrane preparation, cells at a late exponential phase were harvested and resuspended in a buffer containing 0.4 M sucrose, 50 mM MOPS, pH 7.0, 10 mM NaCl, 5 mM EDTA, and 0.5 mM PMSF. Cells were broken using a bead beater and the membranes were isolated by differential centrifugation [17,18]. The chlorophyll concentration of each membrane preparation was measured in 80% acetone using a UV-160 U spectrophotometer (Shimadzu Scientific Instruments, Columbia, MD, USA) [19,20].

### Isolation of integral and membrane associated proteins

The membranes were further purified through washing with 20 mM MOPS, pH 7.0, 50 mM EDTA for 5 times to remove cytoplasmic proteins. The purified membranes were first extracted with 8.0 M urea to release the membrane associated proteins, and then centrifuged at 75,600 × g to pellet the insoluble fraction that was enriched with the integral membrane proteins. The supernatant and the pellet were collected, respectively. The pellet was further extracted with 8.0 M urea, and the supernatant of this extraction was combined with the supernatant from the first extraction and labeled as the peripheral fraction. Similarly, the insoluble pellet from the second extraction was labeled as the integral fraction. The peripheral fraction was diluted four times with deionized water and centrifuged at 75,600 × g to collect the carry-over insoluble fraction; the latter was combined with the previously collected integral fraction. The proteins in the peripheral fraction were precipitated by 10% TCA for 30 minutes on ice. The precipitated proteins were spun down and subsequently extracted with 100% ice-cold acetone to remove lipids and pigments. The integral fraction was also washed with ice-cold acetone multiple times until the wash acetone was colorless. Both fractions were dried under a vacuum, solubilized with the multiple surfactant solution (5.0 M urea, 2.0 M thiourea, 2.0 mM TBP, 2% CHAPS, 2% sulfobetaine 3–10, 0.5% carrier ampholytes, 40 mM Tris, 0.001% orange G dye), and sonicated for 15 minutes in a waterbath at 4°C. During this step, nearly all proteins in the peripheral fraction were dissolved, whereas the integral proteins were only partially solubilized. The insoluble parts in both fractions were removed by centrifugation at 75,600 × g. Protein concentration of both fractions was measured with Bio-Rad Dc Protein assay kit (Bio-Rad, Richmond, CA, USA).

### 2-DE of membrane proteins

The immobilized pH gradient strips with different pH ranges (18 cm, pH 3–10, non-linear, pH 4–7, pH 4–5, pH 5–6, Pharmacia Biotech, Uppsala, Sweden) were rehydrated by 320 μl sample solutions containing approximately 500 μg of proteins from the corresponding fractions. Active rehydration was accomplished by applying low voltage [21] for 10 hours after 2-hour rehydration without voltage at 20°C. The first-dimensional IEF for pH 3–10 and pH 4–7 was performed with an IPGphor instrument (Pharmacia Biotech, Uppsala, Sweden) using the following voltage settings: 100 V for 0.5 h, 300 V for 0.5 h, 1,000 V for 0.5 h, 2,500 V for 0.5 h, 5,000 V for 0.5 h, and then 8,000 V until a total of 80,000 Vh was reached. For narrow pH range IPG (pH 4–5 and pH 5–6), the settings were the same except that a total of 12,000 Vh was reached. Upon electrophoresis, the proteins on the strips were denatured and cysteinyl residues were reduced by equilibrating the IPG strips with a buffer containing 6.0 M urea, 2% SDS, 0.375 M Tris/HCl, pH 8.8, 20% glycerol, 5.0 mM TBP, and 2.5% acrylamide monomer for 20 minutes. The second-dimensional electrophoresis was performed using 12–18% gradient SDS-PAGE gels. Upon electrophoresis, the protein spots on the SDS-PAGE gel were stained with colloidal Coomassie Brilliant Blue (CBB) and the gels were scanned using GS-800 Calibrated Imaging Densitometer (Bio-Rad, Richmond, CA, USA) to obtain images for analysis by Melanie II software [22,23].

### Mass spectrometry

Protein spots that were visualized with CBB were excised manually and incubated at 37°C with 2.5 mM Tris HCl (pH 8.5) in 50% acetonitrile to remove the dye bound to the proteins. The gel pieces were dried under a vacuum followed by incubation with 10 μl of 10 μg/ml trypsin in 2.5 mM Tris-HCl, pH 8.5 at 37°C for 18 h. The resulting tryptic fragments were eluted by diffusion into 50% acetonitrile and 0.5% trifluoroacetic acid (TFA). Diffusion of peptide fragments was facilitated by ultrasonication in a waterbath at 4°C. One microliter of the tryptic peptides of each sample was mixed with 1.0 μl of α-cyano-4-hydroxy-cinnamic acid matrix prior to be transferred to a 100-well plate for MALDI-TOF. A Voyager-DE PRO Biospectrometry Workstation was used to acquire mass spectra in a reflection-delayed extraction mode over a mass range of 600–4,000 Da. The final mass spectra were the accumulation of the spectra obtained from 3–6 positions with 64 shots (total 192–384 shots). If high resolved mass spectra could not be obtained for a spot, the sample would be concentrated by drying again using a vacuum followed by resuspension in 2.5 μl of elution buffer and 2.5 μl of 2.5 mM Tris HCl (pH 8.5). The increased peptide concentrations allowed mass spectral detection of any protein spots that could be visualized with CBB. Some proteins had fewer tryptic sites and did not produce enough tryptic peptides within the mass range 600–4,000 Da for the identification. For such proteins, spectra within the mass range 3,000–6,000 Da were also acquired to ensure good resolution for the spectra between 3,000–5,000 Da. Subsequently, the peptide mass fingerprints (PMF) generated from the two mass ranges were combined as one PMF file to search the database. The peptide ions generated by autolysis of trypsin (with m/z 832.33^+^, 842.51^+^, 1045.56^+^, 2211.10^+^) were used as the internal standard peaks for mass calibration. The mass spectra were analyzed and the PMF for each sample was generated with the Data Explorer software.

### Data analysis

Peptide masses were used to search NCBInr databases with the MS-Fit program  using the following parameters: mass tolerance of 25 ppm, a minimum of two peptides match with one missed cleavage. The identity of proteins with high scores in the MS-Fit analysis was further validated by three other criteria: mass, p*I *and amino acid sequence coverage. The deduced mass of the putatively identified proteins should match the apparent mass estimated from the corresponding spots on the 2-D gel. In the mass comparisons, we considered possible post-translational modifications (PTMs) which could increase or decrease the mass. The second criterion is the deduced p*I *of proteins, which should be close to the p*I *estimated from the 2-D gel. Again, the possibility of PTMs that may change p*I *was considered. The last criterion is the amino acid sequence coverage which delineates the ratio of the number of identified amino acid residues to the total number of amino acid residues for each individual protein. Generally the higher the sequence coverage, the more confident the identification is. However, it should be noted that the sequence coverage of a protein strongly depends on the chemophysical property of its amino acid sequence and the abundance. Sequence coverage information is particularly important for the identification of low molecular weight proteins, e.g. photosystem I PsaE subunit [82% coverage, see additional file 1], because their MOWSE scores are usually lower compared with those of high molecular weight proteins due to the smaller number of distinct tryptic peptides. In addition to these criteria, correspondence to each of the identified proteins in the Cyano2Dbase was also used as a positive control. If a protein spot is identified as the same protein represented by a spot in Cyano2Dbase and their coordinates (apparent protein mass and p*I*) are also matched, then the identification for this particular protein is considered to be further confirmed.

The motif and domain of hypothetical proteins were predicted by InterPro program . Hydropathy analysis for deduced protein sequences was performed by predicting transmembrane (TM) helices using TopPred program . The cut-off for TopPred scores was set to 0.6 to include only high confident TM prediction [24].

### Conservative analysis of Synechocystis proteins

The amino acid sequences of *Synechocystis *proteins were retrieved from Cyanobase automatically and saved into a file in fasta format using an in-house software. The entire proteome sequences of *Arabidopsis thaliana *(thereafter *Arabidopsis*) were retrieved from The Arabidopsis Information Resource (TAIR, ). *Synechocystis *proteins and their *Arabidopsis *homologs were analyzed using the software Blastpro [25], which automatically searches the *Arabidopsis *proteome for the homolog of each *Synechocystis *protein.

## Results

### Prefractionation of membrane proteins

**Figure 1:**
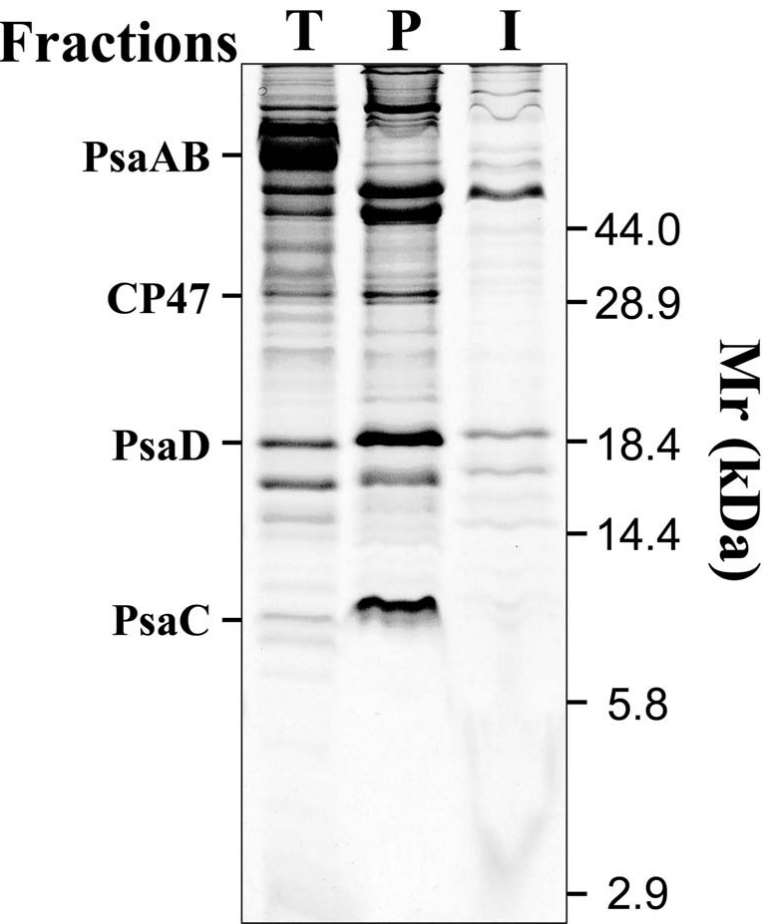
**Separation of integral and membrane associated proteins**. The total *Synechocystis *membranes were treated with 8.0 M urea to release the membrane associated proteins. The supernatants containing the released membrane associated proteins were collected as the peripheral fraction after centrifugation, and the insoluble pellet was collected as the integral fraction. The proteins in the peripheral fraction were further precipitated with 10% TCA, washed with ice-cold acetone, and re-solubilized by the multi-surfactant solution. The integral proteins were directly washed with acetone and solubilized with the same volume of multi-surfactant solution. Equal volume of samples from the peripheral fraction (Lane P) and the integral fraction (Lane I) were separated by SDS-PAGE. Total membranes containing 10 μg of chlorophyll were used as the loading control (Lane T). All the samples were incubated with denaturing dye (2% SDS and 0.1 M DTT) at room temperature for 4 h before separated by SDS-PAGE.

The low abundance of some membrane proteins is one of the major hindrances to their identification and functional characterization. To analyze the membrane proteome of *Synechocystis*, we first enriched low abundant proteins by separating membranes into the peripheral and integral fractions. The peripheral fraction mainly contains the membrane associated proteins that were released from the lipid-bilayer by 8.0 M urea extraction, whereas the integral fraction mainly contains the integral membrane proteins that are refractory to the urea extraction. The fractionated proteins were separated by SDS-PAGE and stained with CBB. The result revealed that the protein migration patterns of the two fractions are not only different from each other, but also different from those of the total membrane proteins (Figure 1). The difference of the protein patterns was exhibited by the different dominant protein bands on the SDS-PAGE gel that are specific to either fraction, but not both (Figure 1), indicating that the fractionation is an efficient way to separate membrane associated proteins from integral proteins, which is in agree with the literature [4,5]. The 68 kDa major bands representing high abundant integral proteins PsaA and PsaB were present only in the lane for the total membrane proteins but not in the other two lanes, indicating that these two 11-TM containing highly hydrophobic proteins were not solubilized by the extraction buffer. In fact, no report has shown that these proteins can be solubilized in a buffer compatible for 2-DE. Both the peripheral and integral fractions contain many high molecular weight proteins that were not detected in our previous study using TCA/acetone precipitation of total membranes (Figure 1) [6]. Although the multiple surfactant solution used here is stronger than the rehydration buffer used in our previous study [6], detection of more high molecular weight proteins is more likely due to enrichment of low abundant proteins by serial extraction rather than the difference of solubilizing solution. This is supported by the observation that the serial extraction resolved more high molecular weight proteins than TCA/acetone precipitation did even we used the same solution for protein solubilization (unpublished data). Therefore, the serial extraction is an effective way for enriching low abundant membrane proteins.

### 2-D separation and identification of the fractionated proteins

**Figure 2:**
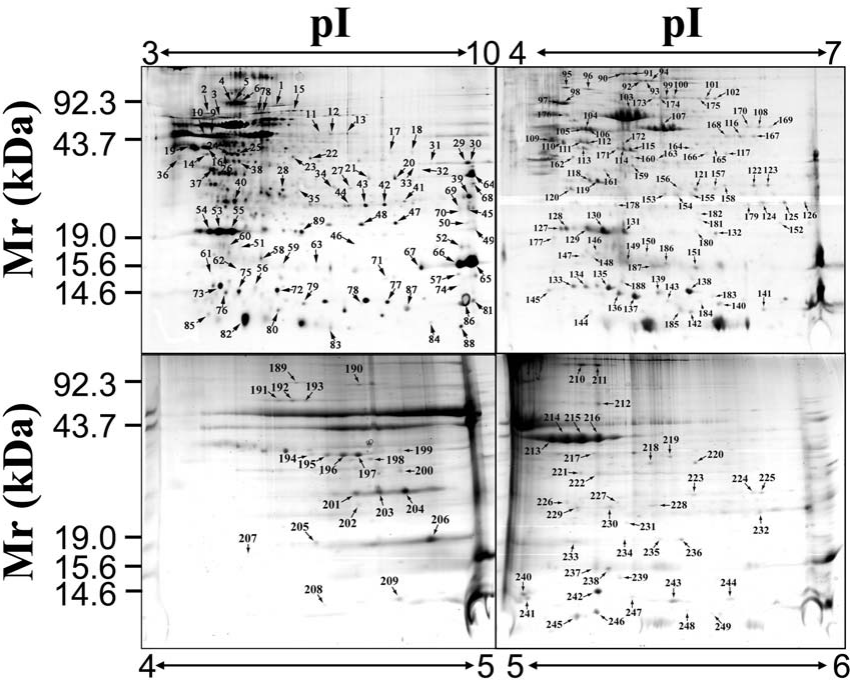
**Separation of membrane associated proteins by 2-DE**. The membrane associated proteins in the peripheral fraction were separated using 18-cm IPG strips covering pH ranges 3–10 (nonlinear), 4–7, 4–5, and 5–6 for the first dimension, and then separated by 12–18% gradient SDS-PAGE gels for the second dimension. Each gel shown is a representative of the three replicates that has the best resolution. All the labeled protein spots in the 2-D gels were analyzed by mass spectrometry and identified using peptide mass fingerprinting.

**Figure 3:**
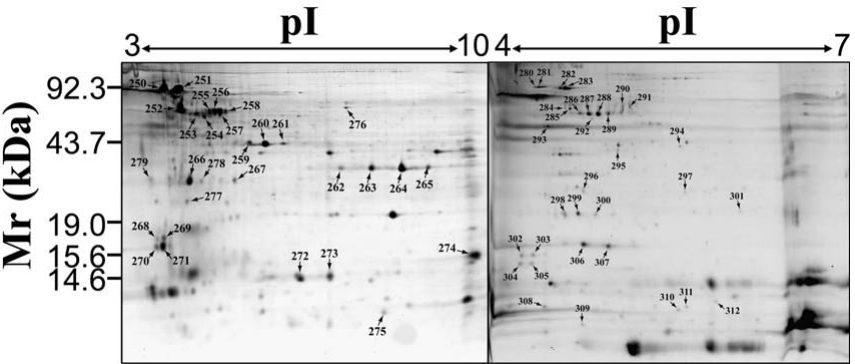
**Separation of integral membrane proteins by 2-DE**. The integral proteins were separated using 18-cm IPG strips covering pH ranges 3–10 (nonlinear), and 4–7 for the first dimension, and then separated by 12–18% gradient SDS-PAGE gel in the second dimension. Each gel shown is a representative of the three replicates that has the best resolution. All the labeled protein spots in the 2-D gel were analyzed by mass spectrometry and identified using peptide mass fingerprinting.

To further enrich low abundant membrane proteins for the identification, we separated proteins on 2-D gels with multiple pH ranges. For the peripheral fraction, we used four 2-D gels covering pH ranges 3–10, 4–7, 4–5 and 5–6 (Figure 2). For the integral fraction, we used only two 2-D gels with pH range 3–10 and 4–7 (Figure 3) because the sample complexity in this fraction was relatively low compared with the peripheral fraction (Figure 1). Each gel had three replicates and only the protein spots presented in all replicates were chosen for PMF identification. In total, more than 600 protein spots were observed on 2-D gels for the peripheral fraction, and more than 200 protein spots were observed on 2-D gels for the integral fraction.

Proteins were identified as previously described [6]. A total of 112 proteins were identified from 312 spots, including 95 proteins represented by 249 spots in the peripheral fraction, and 29 proteins represented by 63 spots in the integral fraction [see additional file 1]. It is notable that 12 proteins were present in both the fractions. These proteins may contain differential PTMs that allow different subpopulations of these proteins to differentially associate with the membranes, because PTM appears to be common for membrane proteins as evidenced by many proteins with multiple isoforms [Figure 2 and 3; see additional file 1]. Nevertheless, the majority of the identified proteins in the integral fraction were not found in the peripheral fraction or *vice versa*, indicating that the fractionation method is effective. Forty of these proteins contain multi-isoforms that appeared as multiple-spots with equal mass but different p*I *values on 2-D gels, an indicative of PTMs causing the protein p*I *shift. The topology analysis using the software TopPred [6,24] revealed that 20 out of 29 (69.0%) proteins in the integral fraction contain at least one TM [see additional file 2], including the 4-TM containing protein Slr0891. The 9 predicted non-TM containing proteins may either tightly interact with the membrane embedded proteins, or may associate with the membranes through post-translationally attached lipid components. For example, the protein Sll1450 was predicted to be a lipoprotein by the algorithm LipoP, which correctly predicts 96.8% of lipoproteins in Gram-negative bacteria [26]. In contrast, 16 out of 95 (16.8%) proteins in the peripheral fraction were predicted to contain one TM (data not shown). The presence of TM containing proteins in the peripheral fraction is possibly due to the cross contamination caused by incompletely spinning down of insoluble fraction during fractionation. However, the higher ratio of TM-containing proteins in the integral fraction strongly suggests that integral membrane proteins were specifically enriched by the serial extraction approach. The majority of the proteins (77 out of 112) identified here were not identified in our previous study, where 51 proteins were identified from the total membranes without prefractionation [6]. Among the 128 proteins identified by these two studies, 41 proteins were also described in Cyano2Dbase [27,28]. It should be mentioned that only 5 proteins were exclusively identified by this study, and the rest of the proteins have been previously identified by others using either 2-DE based proteomic approach or shotgun proteomic approach (see the discussion).

### Analysis of the subcellular localization of the identified membrane proteins

The *Synechocystis *is a Gram-negative cyanobacterium containing a plasma membrane, an outer membrane, and a photosynthetic thylakoid membrane. Seventy-two, 29, and 76 proteins have been identified from each purified membrane by several consecutive studies [3-5,8], respectively. Because we did not separate the membranes into such three fractions, an identified protein in additional file 1 may come from any of the three distinct membrane fractions mentioned above. It should be mentioned that the proteins identified from our previously work might come from any of the three distinct membrane fractions as well [6]. Comparisons of the proteins in additional file 1 and those identified by the above studies revealed that 34 proteins were matched as the thylakoid membrane proteins [5], 33 proteins were matched as the plasma membrane proteins [4,8], and 15 proteins were matched as the outer membrane proteins [3]. Several interesting observations were also obtained from the comparisons. First, 11 proteins were matched as both the thylakoid and plasma membrane proteins, *e.g.*, the subunits of the photosystem I/II complexes and the subunits of ATP synthase [see additional file 1]. This is not surprising because accumulating evidence suggested that the plasma membrane is involved in the early steps of the biogenesis of the photosystem [8,29]. Furthermore, membrane vesicles transport from the plasma membrane to the thylakoid membrane or *vice versa *could also facilitate the exchange of the protein components between the two membrane systems [8,30,31]. Second, 6 proteins were matched as both the plasma membrane and outer membrane proteins, including two porins (Slr1841, Slr1908) and four hypothetical proteins (Slr1506, Sll1835, Slr0431 and Slr1270). Finally, 2 proteins were present in all of the three membrane systems, including photosystem II subunit PsbQ (Sll1638) [32-34] and the iron transport system substrate-binding protein Slr1295. Both are lipoproteins predicted by LipoP [26]. The last two observations are intriguing and cannot simply be explained by the cross contamination of distinct membrane fractions because the data used for the comparison were generated from the completely separated membrane fractions [3-5,8]. A more reasonable explanation is that there are interactions or transport processes between these membrane systems causing the exchange of the protein components, yet the type and function of the interactions and transport processes remain to be further characterized [3,5,8].

In addition to the overlapping proteins whose cellular locations were determined by aforementioned studies, 54 proteins identified by the current study were not assigned a subcellular location because they were not identified by the above studies. Some of these proteins are known to be the thylakoid membrane associated proteins including multiple photosystem I and II related proteins, *e.g.*, the photosystem II subunit PsbU (Sll1194), photosystem II oxygen-evolving complex 23 K protein PsbP homolog (Sll1418), and photosystem I assembly related protein (Slr0823) [see additional file 1]. The data suggest that the serial extraction of membrane proteins combined with narrow pH range 2-DE separation helped to resolve and identify more *Synechocystis *membrane proteins.

### Functions of the identified membrane proteins

**Figure 4:**
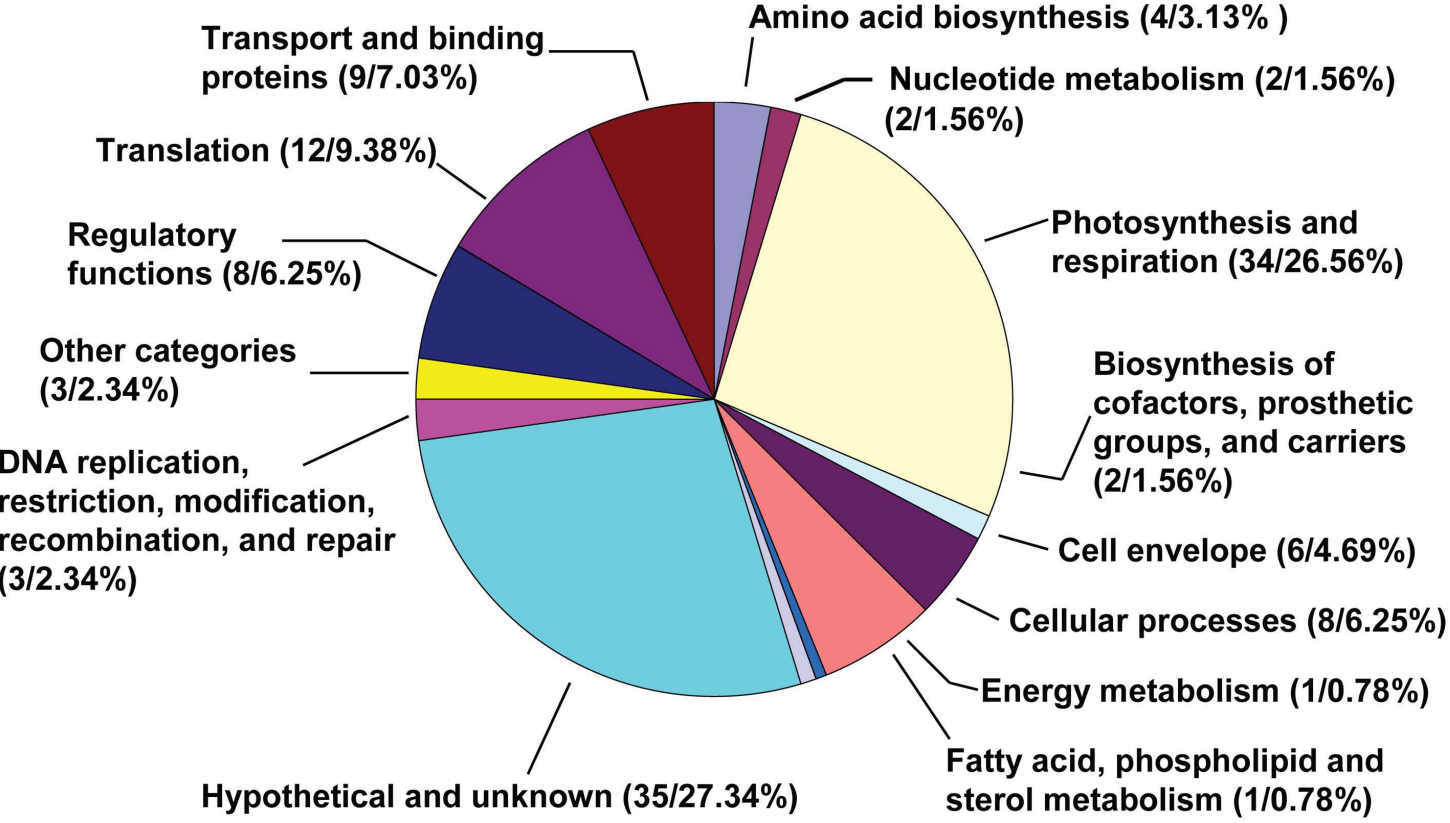
**Functional groups of the identified membrane proteins**. The pie diagram shows that the 128 membrane proteins in additional file 1 are grouped into 14 major categories by their functions assigned in Cyanobase. The total number of proteins in each group and its ratio (percent) to the total number of identified proteins are indicted in the parenthesis.

To better understand the functional diversity and importance of the membrane proteins, we analyzed the cellular function of each identified protein by searching the gene annotations in Cyanobase [35]. The identified proteins can be categorized into 14 different functional groups, which is 87.5% (14 out of 16) of all functional categories of *Synechocystis *proteins described in Cyanobase [35]. Except the hypothetical and unknown proteins, the largest group of the identified proteins is photosynthesis and respiration (Figure 4), which consists of 26.56% (34 out of 128) of the total identified proteins. In this functional group, some components of the major functional protein complexes in photosynthetic and respiratory electron transport chains were identified. For example, in the photosynthetic process, proteins from photosystem I (Ssl0563, Ssr2831, and Slr0737), photosystem II (Slr2034, Sll0427, Sll1418, Sll1194, Sll1398, and Sll1638), cytochrome *b*_6_*f *complex (Sll136, Ssr2998), and ATP synthase subunits (Sll1326, Sll1325, Sll1324, Sll1323, and Slr1330) were identified [32-34,36,37], whereas in the respiratory process, NADH dehydrogenase subunits (Slr0261, Slr1280, Slr1281, Slr1623, and Ssl1690) were also identified [21,38-40]. Moreover, multiple phycobilisome subunits were identified, including the proteins Sll1580, Sll1577, Slr2067, and Slr1986. These proteins are known to be the thylakoid membrane associated proteins involved in photosynthesis [41].

### Conservative analysis of the identified membrane proteins

**Figure 5:**
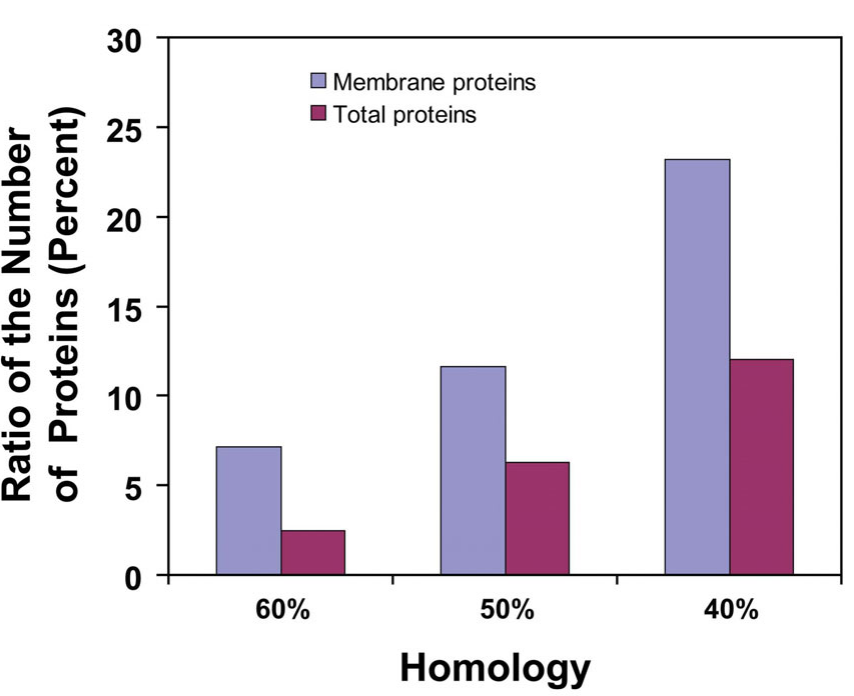
**Homology analyses for the identified membrane proteins between *Synechocystis *and *Arabidopsis***. For the comparison, the amino acid sequences of the 128 identified membrane proteins and the entire *Synechocystis *proteome were blasted against the entire *Arabidopsis *proteome using Blastpro, respectively. The bars represent the ratio (percent) of the number of *Synechocystis *proteins that have *Arabidopsis *homologs with sequence similarity higher than or equal to 40%, 50%, and 60% relative to the total number of *Synechocystis *proteins for each comparison.

Cyanobacteria are considered to be the ancestor of chloroplast in higher plants [42]. In this context, many proteins are expected to be conserved between *Synechocystis *and higher plants, e.g., *Arabidopsis*, for performing conserved functions. However, *Synechocystis *is an independent organism requiring diversified functions to support life cycles, whereas the chloroplast of *Arabidopsis *is a subcellular organelle specialized for a certain type of functions, *e.g.*, photosynthesis. Therefore, some proteins that are essential in *Synechocystis *may not be necessary for the proper functioning of chloroplast in plants. This functional redundancy may facilitate the release of the selection pressure that favors the conservation of these proteins, which will gain diversified functions and amino acid sequences through evolution. To investigate which proteins are more phylogenically conserved between *Synechocystis *and *Arabidopsis*, we performed the conservative analysis for all *Synechocystis *proteins by automatically searching the *Arabidopsis *proteome using Blastpro, an automatic Blast software for the analysis of the homology between two lists of proteins [25,43]. In total, 393 (12.0%), 206 (6.3%), and 81 (2.5%) *Synechocystis *proteins have homologs in *Arabidopsis *with minimal sequence similarity 40%, 50%, and 60% respectively (Figure 5). To investigate the conservation of the membrane proteins, we performed a similar analysis for the membrane proteins using Blastpro [25]. Of the identified 128 proteins, 30 (23.4%), 17 (13.3%), and 9 (7.0%) proteins have homologs in *Arabidopsis *with minimal sequence similarity 40%, 50%, and 60%, respectively (Figure 5). Interestingly, the ratio of the highly conserved proteins, that is, proteins with minimal sequence similarity 40%, 50% or 60% in the membranes, is much higher than that in the total proteins (Figure 5). This result suggests that the identified membrane proteins are more evolutionally conserved than the rest of the proteins in the *Synechocystis *proteome, probably due to the necessity of the functional conservation of the corresponding proteins.

### Products of the identified hypothetical genes

The genome of *Synechocystis *contains approximately 50% hypothetical or unknown genes that encode proteins with unknown functions. From our current and previous studies [6], we identified a total of 35 (27.34%) hypothetical and unknown proteins based on the information in Cyanobase and the literature (Figure 4). The majority of hypothetical proteins identified are low abundant because they were shown as weakly-stained spots on 2-D gels. The low abundance might prevent the previous discovery and functional analysis of these proteins. Several hypothetical proteins were present as major spots in 2-D gels with multiple isoforms, for example, the hypothetical protein encoded by the ORF *slr1506*, which was shown as the dominant spots at 68 kDa with 4 isoforms in the 2-D gel with pH 3–10 [see additional file 1 and Figure 3]. The unknown functions of these abundant hypothetical proteins may be due to their insolubility in normal aqueous solutions, which hampers their functional analysis.

To gain preliminary functional information of these hypothetical proteins, we performed the functional domain and motif analysis using the computational software InterProScan . The results revealed that 12 hypothetical proteins contain one or more domains and motifs [see additional file 3]. For examples, the protein encoded by ORF *slr1506 *has two domains, including the esterase/lipase/thioesterase domain and the ATP/GTP-binding site motif A. The esterase/lipase/thioesterase domain indicates that the protein has the hydrolase activity whereas the ATP/GTP-binding site motif A suggests that the activity is driven by the energy from ATP or GTP. The protein encoded by ORF *slr0038 *belongs to the mitochondrial energy transfer protein (carrier proteins) family that are found in the inner mitochondrial membrane [44-48]. In *Synechocystis*, the thylakoid membrane contains the machinery for energy metabolism such as the photosynthesis and respiration electron transport chains [38]. Therefore, it is highly possible that the hypothetical protein encoded by *slr0038 *is a substrate carrier protein functionally related with the thylakoids.

Functional characterization of these proteins remains a challenge. Although computational prediction of functional domains of these proteins could partially alleviate this problem by gaining functional information from the domains themselves and the proteins that they may interact with, reverse genetic approach to generate gene knockout mutants and relevant biochemical and physiological characterizations are necessary to understand the detailed functions of these hypothetical proteins.

## Discussion

Membrane proteins contain integral proteins as well as membrane associated proteins linked to the lipid bilayer through direct protein-protein interactions or PTMs (e.g., lipid-anchored proteins). The membrane associated proteins can be released using organic solvents [49], high pH solutions [11,50], or chaotropes [51,52]. Here we used a high concentration of urea to separate the *Synechocystis *membranes into the integral and peripheral fractions; the latter mainly contains membrane associated proteins. Detection and identification of many proteins that are weakly stained on 2-D gels suggest that the fractionation of the membranes effectively enriched low abundant membrane proteins [Figure 1, 2, and 3; see additional file 1] probably because these proteins were unable to be detected in our previous study [6]. Furthermore, the ratio of TM-containing proteins in the integral fraction is much higher than in the peripheral fraction, indicating that the fractionation approach specifically enriched integral membrane proteins. As stated previously, high concentration of urea has been successfully used to enrich integral membrane proteins in other studies [4,5]. Collectively, the data suggest that the high concentration of urea is an effective way to separate membrane associated proteins and integral proteins for proteomic studies.

*Synechocystis *has its unique membrane and soluble compartments. The membrane compartments can be separated into the outer, plasma and thylakoid membranes whereas the soluble compartments can be separated into the periplasm, cytoplasm and lumen [53]. All the membrane and lumenal spaces have their unique protein compositions, which are related to their specific functions in each subcellular location. It is of importance to precisely identify the subcellular locations of proteins, where their functions can be predicted. A number of studies have been recently reported to analyze the *Synechocystis *proteome. These studies were focused on proteins either in specific subcellular locations including the outer membrane [3,53], plasma membrane [4,8,10,53,54], thylakoid membrane [5,53], periplasm [53,55,56], or cytoplasm [56], or in the more general locations [57] such as membrane fraction [6,58,59], soluble fraction [60-62], or both [27,28,63-67]. To summarize these works and catalog the proteins with known or predicted subcellular locations, we generated a table [see additional file 4] that contains all the *Synechocystis *proteins identified based on the references above and the proteins identified in this study. In total, about 47.3% of *Synechocystis *proteome (1,738 proteins) have been identified [see additional file 4]. Specific subcellular locations of 173 identified proteins (10.0%) are determined (outer membrane: 10 proteins; plasma membrane: 75 proteins; periplasm: 52 proteins; thylakoid membrane: 36). For the rest of 1,565 proteins, they either have more than one locations in a cell or their exact location(s) has (have) not been determined by experiments. The proteins with undetermined cellular localizations were assigned as predicted membrane (PM)- or predicted soluble (PS)-proteins based on the TM prediction by TopPred [see additional file 4] [24]. Among the 1,738 proteins, 1,291 proteins (74.3%) were uniquely identified by LC-MS/MS based shotgun proteomic approach [63-67]. The remaining 447 proteins were identified either uniquely by 2-DE based proteomic approach, or by both. Shotgun proteomic approach provides unprecedented power in protein identification and the majority of the proteins in additional file 1 were also identified by this approach. However, five new proteins identified in the current study have never been identified in any previous proteomic studies, including phosphate transport ATP-binding protein PstB (Sll0684), transcriptional repressor SmtB (Sll0792), and three hypothetical proteins (Sll1630, Sll1862, and Slr1053) [see additional file 1]. Identification of these new proteins is more likely due to the protein prefractionation that reduces sample complexity and enriches low abundant proteins. Therefore, we expect that sample prefractionation combined with LC-MS/MS based shotgun proteomics would provide higher coverage in proteome identification in the future.

In spite of the collective efforts in cataloging the *Synechocystis *proteome, 52.7% of the proteome (1,935 proteins) has not been identified so far [see additional file 5]. Multiple possibilities could account for the failure of identification for these proteins (i) some proteins are transiently expressed responding to a specific internal or external signal; (ii) proteins with high hydrophobicity or low abundance are generally difficult to be identified (iii) some proteins may not even express. The identified *Synechocystis *proteome contains 38.7% hypothetical and unknown proteins (673/1,738) [see additional file 6]. In contrast, the unidentified *Synechocystis *proteome contains much higher percent of hypothetical and unknown proteins (1,284/1,935 = 66.4%) [see additional file 7], suggesting that some of hypothetical and unknown proteins might not express. However, experimental evidences are still needed to verify their expression.

Collectively, the information on these identified proteins can be used as a reference for any studies targeting at mechanisms of important biological processes of *Synechocystis*, such as signal transduction cascades stimulated by a specific stimulus. In fact, some proteins were identified from *Synechocystis *under such physiological conditions including heat shock [62], salt stress [10,64], acid stress [56], or heterotrophic condition [60]. In addition, this information can be used for bioinformatic and computational analyses of *Synechocystis *proteome. For example, N-terminal features have been predicted for the outer and plasma membranes, the periplasm, and the thylakoid lumenal proteins using a combined proteomic and multivariate sequence analysis [53].

As mentioned earlier, the purified membranes in the current study contain the thylakoid membrane as well as the plasma membrane and outer membrane since we did not specifically isolate each type of the membrane. However, the abundance of the thylakoid membrane is orders of magnitude higher than that of the plasma membrane and outer membrane [68]. Therefore, the majority of the membrane proteins are predicted to be associated with the thylakoid membrane instead of the plasma or outer membranes. In fact, more than 50% of the proteins with the assigned subcellular locations [3-5,8] in additional file 1 are specific to the thylakoid membrane. This ratio allows us to deduce that the majority of the proteins in additional file 1 without the assigned subcellular locations may be also specific to the thylakoid membrane.

Among the proteins in additional file 1, 34 identified proteins are involved in the processes of photosynthesis and respiration. This group consists of 26.56% of the total identified membrane proteins, whereas only 3.89% of the whole *Synechocystis *proteome belong to this group. The data suggest that these proteins are highly abundant in the *Synechocystis *membranes, indicating that one of the major functions of the *Synechocystis *membranes is photosynthesis and respiration. Besides these proteins, the proteins involved in the process of translation are also highly represented, including the elongation factor EF-Tu and ribosomal proteins [see additional file 1 and Figure 4]. This is not surprising because ribosomes target to the thylakoid membrane when membrane-targeting proteins are synthesized in both cyanobacteria and chloroplasts of higher plants [69,70].

One of the interesting findings from our study is that the identified membrane proteins are more conserved than the other proteins in *Synechocystis*, suggesting that the membrane proteome is more conserved than the rest of the proteome. The protein sequence conservation through evolution ensured the conservation of one of the major functions of the membranes, photosynthesis, between *Synechocystis *and the chloroplasts of higher plants that shares the nearly identical mechanism for the photosynthetic electron transport on the thylakoid membrane [71].

## Conclusion

By using the serial extraction approach to separate membranes into the peripheral and integral fractions, we identified 112 membrane proteins from *Synechocystis*. The identified proteins are involved in 14 distinct groups of functions, which is an indicative of the diversified functions of the *Synechocystis *membrane system. Conserved analysis revealed that membrane proteins are evolutionally more conserved than soluble proteins, suggesting that membranes may perform more conserved functions through evolution.

Although the 2-DE based proteomic approach used here is less robust compared with the LC-MS/MS based shotgun proteomic approach, five of the proteins identified in this study were not identified previously by any shotgun proteomic studies, indicating that sample prefractionation is important for the identification of low abundant proteins. Therefore, we suggest that sample prefractionation combined with LC-MS/MS shotgun proteomic approach would achieve higher proteome coverage in protein identification.

The proteins identified in this study, together with previously identified proteins by others [3-5,8,10,27,28,53-67], will provide the most comprehensive database to date for the identified *Synechocystis *proteins. This database will serve as a useful reference for future studies to understand the mechanisms of basic biological processes of this photosynthetic organism.

## Abbreviations

CBB: Coomassie Brilliant Blue; CHAPS: 3-[(3-cholamidopropyl)dimethylamonio]-1-propanesulphonate; 2-D: two-dimensional; 2-DE: two-dimensional electrophoresis; DTT: dithiothreitol; EDTA: ethylenediaminetetraacetic acid; IEF: isoeletric focusing; MALDI-TOF: matrix-assistant laser-desporption ionization time-of-flight; MOPS: 3-(*N*-morpholino)ethanesulphonic acid; ORF: open reading frame; PAGE: polyacrylamide gel electrophoresis; PM: predicted membrane; PMF: peptide mass fingerprinting; PMSF: phenylmethylsulphonyl fluoride; PS: predicted soluble; PTM: post-translational modification; SDS: sodium dodecyl sulphate; TBP: tributylphosphine; TCA: trichloroacetic acid; TM: transmembrane; Tris: tris(hydroxymethyl)aminomethane

## Competing interests

The authors declare that they have no competing interests.

## Authors' contributions

YW, WX, and PRC conceived the study, YW and WX carried out the proteomics experiments and bioinformatic analysis. PRC and WX provided all the reagents and instruments. YW and WX drafted the manuscript, and all the authors read and approved the final manuscript.

## Supplementary Material

Additional file 1 **All the identified *Synechocystis *sp.** PCC 6803 membrane proteins. Additional file 1 is a MS word table containing a total of 128 proteins with their deduced mass, deduced p*I*, apparent p*I*, mowse score, number of matching peptides, sequence covered by matched peptides, and type of membrane identified in this study and our previous study. Click here for file

Additional file 2 **Transmembrane domain prediction of the identified proteins in the integral fractions.** Additional file 2 is a MS word table containing the proteins with their predicted position of transmembrane domain, score of hydrophobicity, and size using TopPred. Click here for file

Additional file 3 **Functional domain prediction of the hypothetical proteins.** Additional file 3 is a MS word table containing the functional domain and motif analysis of 12 hypothetical proteins identified in this study using the computational software InterProScan. Click here for file

Additional file 4 **The proteins of *synechocystis *sp.** PCC 6803 identified through large-scale proteomic approaches. Additional file 4 is a MS word table containing 1,738 proteins identified based on the references mentioned in the text with their subcellular locations determined by either experiments or predicted by TopPred. Click here for file

Additional file 5 **The proteins of *synechocystis *sp.** PCC 6803 not identified yet by large-scale proteomic approaches. Additional file 5 is a MS word table containing 1,935 proteins not identified based on the references mentioned in the text. Click here for file

Additional file 6 **Functional groups of the identified proteins based on the references mentioned in the text.** Additional file 6 is a pie diagram shows that the 1,738 proteins in additional file 4 are grouped into 17 major categories by their functions assigned in Cyanobase. Click here for file

Additional file 7 **Functional groups of the not yet identified proteins based on the references mentioned in the text.** Additional file 7 is a pie diagram shows that the 1,935 proteins in additional file 5 are grouped into 17 major categories by their functions assigned in Cyanobase. Click here for file
