# Supplementary material for: Identification and bioinformatic analysis of the membrane proteins of synechocystis sp. PCC 6803
Source: Proteome Sci. 2009 Mar 25;7:11. doi: 10.1186/1477-5956-7-11 (PMC2666656; doi:10.1186/1477-5956-7-11)
Supplement: Additional file 1 — All the identified Synechocystis sp. PCC 6803 membrane proteins. Additional file 1 is a MS word table containing a total of 128 proteins with their deduced mass, deduced pI, apparent pI, mowse score, number of matching peptides, sequence covered by matched peptides, and type of membrane identified in this study and our previous study. [file 1477-5956-7-11-S1.doc]

| **Additional file 1. The identified *Synechocystis* sp. PCC 6803 membrane proteins** | | | | | | | | | | |
| --- | --- | --- | --- | --- | --- | --- | --- | --- | --- | --- |
| **Cyanobase accesssion** | **Protein ID** | **No.** | **Deduced mass (Da)** | **Deduced p*I*** | **Apparent p*I*** | **Mowse score** | **Number of matching peptides** | **Sequence covered by matched peptides (%)** | **Type of membrane^†^** | **References** |
|
| sll0020 | ATP-dependent Clp protease regulatory subunit | 1 | 91174.9 | 5.4 | 5.7 | 1.93E+12 | 29 | 43 | T | [5] |
| sll0065 | Acetolactate synthase small subunit | 180 | 18871 | 5.36 | 5.8 | 3.02E+03 | 6 | 36 |  |  |
| sll0224 | Amino-acid ABC transporter binding protein | 279 | 32830.1 | 4.25 | 4.25 | 510 | 4 | 21 |  |  |
| sll0272 | Hypothetical protein | 57 | 17658.5 | 8.73 | 8.95 | 1.26E+04 | 7 | 54 |  |  |
| sll0289 | Septum site-determining protein MinD | 227 | 29047.2 | 5.26 | 5.36 | 1.96E+04 | 9 | 38 |  |  |
| sll0368 | Pyrimidine operon regulatory protein PyrR | 50 | 19946.4 | 9.15 | 9.15 | 1.55E+05 | 11 | 62 |  |  |
| sll0408 | Peptidyl-prolyl cis-trans isomerase | 14, 113, 194, 195, 196, 197 | 43909.7 | 4.71 | 4.7, 4.65, 4.78, 4.46, 4.50, 4.54, 4.58 | 7.24E+03 | 9 | 29 | T | [5] |
| sll0427 | Photosystem II manganese-stabilizing polypeptide | 37, 118, 119, 201, 203, 204 | 29911.8 | 4.82 | 4.78, 4.80, 4.90, 4.57, 4.63, 4.71 | 3.40E+03 | 12 | 45 | P,T | [5],[8] |
| sll0617 | Chloroplast membrane-associated 30 kD protein | 38, 159 | 28905.5 | 4.95 | 5.18, 5.25 | 3.46E+03 | 6 | 27 | P,T | [5],[8] |
| sll0680 | Phosphate-binding periplasmic protein precursor (PBP) | 19, 109 | 40023.4 | 4.57 | 4.5, 4.5 | 1.07E+05 | 9 | 28 |  |  |
| sll0683 | Phosphate transport ATP-binding protein PstB | 34, 35, 223, 225 | 30210.5 | 5.75 | 6.47, 6.00, 5.57, 5.78 | 6.80E+04 | 14 | 42 |  |  |
| sll0684* | Phosphate transport ATP-binding protein PstB | 157, 122, 158 | 29244.6 | 6.13 | 6.05, 6.13, 6.40 | 9.93E+04 | 14 | 28 |  |  |
| sll0772 | Probable porin; major outer membrane protein | 292 | 59146.2 | 4.86 | 4.86 | 318 | 11 | 30 |  |  |
| sll0792* | Transcriptional repressor SmtB | 308 | 15083.4 | 6.41 | 4.5 | 150 | 5 | 68 |  |  |
| sll0829 | Methyltransferase | 41 | 23071.7 | 6.28 | 8.01 | 2.33E+03 | 7 | 29 |  |  |
| sll0947 | Light repressed protein | 42, 43, 44, 124, 125, 126, 232 | 21895 | 6.07 | 7.10, 6.78, 6.59, 6.50, 6.70, 6.90, 5.77 | 5.17E+06 | 14 | 68 | P | [8] |
| sll0998 | LysR transcriptional regulator | 22, 23, 117, 166, 218, 220 | 38017.7 | 5.54 | 6.15, 5.95, 6.15, 5.90, 5.45, 5.58 | 5.31E+05 | 10 | 32 |  |  |
| sll1028 | Carbon dioxide concentrating mechanism protein CcmK | 80, 142, 246, 249 | 11134.8 | 5.33 | 5.78, 5.68, 5.30, 5.63 | 1.69E+05 | 8 | 80 | P | [8] |
| sll1029 | Carbon dioxide concentrating mechanism protein CcmK | 275 | 12102 | 6.11 | 7.7 | 488 | 5 | 43 | P,T | [5],[8] |
| sll1099 | Protein synthesis elongation factor Tu | 15, 101, 102, 107, 175, 210, 211, 212, 213, 214, 215, 216 | 43733.3 | 5.16 | 5.95, 5.95, 6.05, 5.55, 5.88, 5.26, 5.31, 5.32, 5.28, 5.22, 5.26, 5.32 | 1.48E+04 | 9 | 28 | P,T | [5],[8] |
| sll1118 | Hypothetical protein | 248 | 11450 | 5.61 | 5.56 | 1.87E+03 | 7 | 73 | P | [8] |
| sll1194 | Photosystem II subunit PsbU | 145 | 14231.3 | 4.44 | 4.44 | 3.44E+03 | 6 | 57 |  |  |
| sll1196 | Phosphofructokinase | 200 | 38588.4 | 5.87 | 4.7 | 246 | 7 | 16 |  |  |
| sll1260 | 30S ribosomal protein S2 | 221, 222 | 30150.3 | 5.14 | 5.26, 5.31 | 1.85E+03 | 9 | 26 |  |  |
| sll1261 | Elongation factor TS | 154, 155, 228, 229, 230 | 24231 | 5.37 | 5.65, 5.84, 5.47, 5.36, 5.22 | 1.68E+04 | 9 | 37 |  |  |
| sll1306 | Periplasmic protein, function unknown | 20, 21, 262, 263, 264, 265 | 38270.8 | 7.82 | 7.50, 6.80, 6.80, 7.50, 8.03, 8.5 | 6.96E+05 | 14 | 31 |  |  |
| sll1307 | Periplasmic protein, function unknown | 272, 273, 312 | 17848.8 | 6.06 | 5.8 | 906 | 4 | 33 | O | [3] |
| sll1316 | Plastoquinol--plastocyanin reductase | 306, 307 | 18996.5 | 4.87 | 4.79, 5.00 | 703 | 7 | 47 | T | [5] |
| sll1323 | ATP synthase subunit b | 62 | 16245.5 | 5.16 | 5.16 | 3.04E+04 | 13 | 52 | P,T | [5],[8] |
| sll1324 | ATP synthase subunit b | 51 | 19804.6 | 5.08 | 5.2 | 1.50E+04 | 11 | 37 | P,T | [5],[8] |
| sll1325 | ATP synthase d subunit | 89, 132, 235, 236 | 20093.4 | 6.33 | 6.04, 6.04, 5.48, 5.54 | 9.41E+04 | 9 | 42 |  |  |
| sll1326 | ATP synthase a subunit | 290 | 53965.8 | 4.96 | 5.13 | 7.62E+03 | 9 | 18 | P | [5],[8] |
| sll1338 | Unknown protein | 207, 268, 269, 270, 271, 302, 303, 304, 305 | 20183 | 4.52 | 4.26, 4.32, 4.42, 4.32, 4.42, 4.3, 4.39, 4.29, 4.38 | 1.58E+03 | 4 | 30 | O | [3] |
| sll1358 | Putative oxalate decarboxylase | 164, 259, 260, 261, 294 | 43151.2 | 5.81 | 5.8, 5.4, 5.6, 5.8, 5.6 | 5.16E+08 | 17 | 31 | O | [3] |
| sll1363 | Ketol-acid reductoisomerase | 160 | 35822.1 | 4.91 | 5.25 | 3.72E+06 | 12 | 41 | P,T | [5],[8] |
| sll1398 | Photosystem II 13 kD protein | 75, 76, 136, 137, 241 | 12590.4 | 4.96 | 5.16, 4.89, 5.18, 5.24, 5.11 | 8.39E+04 | 7 | 69 | T | [5] |
| sll1418 | Photosystem II oxygen-evolving complex 23K protein PsbP homolog (*psbP2*) | 177 | 20747.3 | 4.55 | 4.5 | 1.48E+04 | 8 | 57 |  |  |
| sll1450 | Nitrate transport 45kD protein | 293 | 48966.9 | 5.19 | 4.53 | 562 | 8 | 23 | P | [4] |
| sll1533 | Twitching mobility protein | 11, 12, 13, 108, 169, 170 | 47899.1 | 5.84 | 6.34, 6.45, 6.59, 6.45, 6.59, 6.34 | 4.48E+06 | 16 | 36 |  |  |
| sll1577 | Phycocyanin b subunit | 53, 54, 55, 129, 130, 131, 146, 150, 206, 233 | 18126.6 | 4.98 | 4.85, 4.70, 4.98, 4.70, 4.98, 5.04, 4.91, 5.36, 4.78, 5.23 | 2.27E+06 | 11 | 70 | P,T | [4],[5] |
| sll1578 | Phycocyanin a subunit | 58, 59, 149, 151, 186, 187, 237, 238 | 17586.7 | 5.35 | 5.64, 5.82, 5.19, 5.72, 5.55, 5.42, 5.30, 5.34 | 1.78E+04 | 7 | 43 | P,T | [5],[8] |
| sll1580 | Phycocyanin associated linker protein | 29, 30, 31, 32, 33, 162 | 32520.8 | 9.35 | 9.15, 9.20, 8.6, 8.2, 8.06, 9.3, 4.69 | 2.55E+08 | 17 | 56 | P | [4] |
| sll1581 | GumB protein | 254 | 54125.4 | 4.97 | 4.97 | 7.52E+09 | 17 | 50 | O | [3] |
| sll1583 | DNA ligase | 92, 93, 94 | 61469 | 5.07 | 5.31, 5.38, 5.43 | 9.24E+06 | 13 | 22 | T | [5] |
| sll1626 | SOS function regulatory protein | 179, 301 | 22744.3 | 5.84 | 6.34, 6.09 | 1.35E+03 | 5 | 25 | T | [5] |
| sll1630* | Unknown protein | 63 | 15796.1 | 5.08 | 6.25 | 643 | 4 | 37 |  |  |
| sll1638 | Photosystem II subunit PsbQ | 143 | 16477.1 | 6.73 | 5.57 | 718 | 6 | 48 | P, O, T | [3],[5],[8] |
| sll1679 | Protease HhoA | 165, 217, 219 | 41336.3 | 6.34 | 6.02, 5.29, 5.52 | 1.74E+06 | 15 | 45 |  |  |
| sll1745 | Ribosomal protein L10 | 52 | 18675.7 | 8.64 | 8.95 | 1.22E+03 | 6 | 41 |  |  |
| sll1746 | Ribosomal protein L12 | 73, 135 | 13259.4 | 4.74 | 4.85, 5.02 | 613 | 6 | 59 |  |  |
| sll1762 | Putative polar amino acid transport system substrate-binding protein | 120 | 41985.4 | 4.8 | 4.67 | 1.89E+04 | 9 | 30 |  |  |
| sll1767 | 30S ribosomal protein S6 | 74 | 13237.3 | 7.93 | 8.95 | 1.78E+03 | 5 | 46 |  |  |
| sll1808 | 50S ribosomal protein L5 | 49 | 20230.5 | 9.69 | 9.3 | 2.33E+08 | 16 | 69 |  |  |
| sll1815 | Adenylate kinase | 234 | 20453.6 | 5.39 | 5.39 | 2.39E+03 | 8 | 61 |  |  |
| sll1835 | Periplasmic protein, function unknown | 298 | 28832.3 | 4.88 | 4.63 | 5.14E+03 | 6 |  | P, O | [3],[8] |
| sll1862* | Unknown protein | 71 | 15194.2 | 5.65 | 7.1 | 1.52E+03 | 4 | 28 |  |  |
| sll1915 | Carbonic anhydrase | 27 | 34806.6 | 5.45 | 6.62 | 2.43E+05 | 9 | 41 |  |  |
| sll1941 | DNA gyrase subunit A | 85 | 8171.5 | 8.9 | 4.7 | 47.9 | 2 | 46 |  |  |
| slr0012 | Ribulose bisphosphate carboxylase small subunit | 183 | 13239.1 | 5.36 | 6.03 | 754 | 4 | 38 | T | [5] |
| slr0013 | Hypothetical protein | 311 | 18689.9 | 8.96 | 5.67 | 5.99E+03 | 7 | 43 | P(I) | [4],[8] |
| slr0038 | Hypothetical protein | 188 | 15240.5 | 4.83 | 5.15 | 3.20E+03 | 5 | 55 |  |  |
| slr0151 | Unknown protein | 24, 25, 26, 114, 115, 171, 172, 295 | 34915.2 | 4.95 | 4.98, 5.17, 4.85, 5.17, 5.22, 5.11, 5.17, 5.09 | 1.41E+06 | 13 | 25 | P | [4],[8] |
| slr0161 | Twitching motility protein | 17, 18 | 40616.2 | 6.57 | 7.35, 8.05 | 8.23E+04 | 12 | 34 |  |  |
| slr0165 | ATP-dependent Clp protease proteolytic subunit | 181 | 22396.9 | 5.74 | 5.92 | 945 | 6 | 26 |  |  |
| slr0244 | Hypothetical protein | 153, 226 | 31204.5 | 5.13 | 5.56, 5.22 | 1.28E+04 | 7 | 34 |  |  |
| slr0261 | NADH dehydrogenase subunit 7 | 116, 167, 168 | 45534.7 | 5.75 | 6.27, 6.40, 6.13 | 4.23E+09 | 19 | 37 | T | [5] |
| slr0362 | Hypothetical protein | 46 | 20576.4 | 5.92 | 6.2 | 2.28E+06 | 12 | 66 |  |  |
| slr0431 | Hypothetical protein | 266, 278, 296 | 27011.9 | 7.93 | 4.78, 4.95 | 1.19E+05 | 9 | 40 | P, O | [3],[8] |
| slr0447 | Negative aliphatic amidase regulator | 9, 10, 104, 105, 106 | 48359.8 | 4.82 | 4.78, 4.65, 4.78, 4.82, 4.86 | 8.51E+07 | 13 | 39 | P | [4],[8] |
| slr0552 | Hypothetical protein | 40 | 26721.6 | 4.91 | 4.91 | 1.80E+05 | 8 | 43 |  |  |
| slr0670 | Hypothetical protein | 28, 121, 156, 297 | 32677 | 5.38 | 5.82, 5.84, 5.67, 5.65 | 3.19E+08 | 14 | 73 |  |  |
| slr0729 | Hypothetical protein | 144 | 10946.7 | 4.63 | 4.72 | 1.12E+03 | 5 | 61 | T | [5] |
| slr0731 | Hypothetical protein | 176 | 44716.3 | 4.42 | 4.63 | 1.87E+05 | 11 | 29 |  |  |
| slr0737 | Photosystem I subunit PsaD | 64, 65, 66, 67, 68, 69, 70, 274 | 15643.9 | 8.95 | 9.3, 9.3, 8.95, 8.3, 9.3, 8.95, 8.95, 9.3 | 1.18E+03 | 6 | 52 | P,T | [5],[8] |
| slr0823 | Photosystem I assembly related protein (ycf3) | 178 | 19878.3 | 4.72 | 5.09 | 9.11E+07 | 12 | 71 |  |  |
| slr0848 | Hypothetical protein | 161 | 31790.6 | 4.83 | 4.98 | 112 | 5 | 20 | P | [4] |
| slr0891 | N-acetylmuramoyl-L-alanini-amidase | 276 | 64377 | 5.8 | 7 | 2.04E+04 | 10 | 17 |  |  |
| slr0929 | Chromosome partitioning protein, ParA family | 45 | 21699.2 | 8.83 | 9.15 | 3.51E+06 | 13 | 74 |  |  |
| slr1034 | Hypothetical protein | 148 | 14408.4 | 4.67 | 4.88 | 2.85E+03 | 7 | 39 | T | [5] |
| slr1053* | Unknown protein | 267 | 29848.2 | 5.29 | 5.3 | 2.84E+04 | 11 | 44 |  |  |
| slr1227 | Chloroplast import-associated channel IAP75 | 95, 189, 250, 280, 281 | 92307.8 | 4.29 | 4.6, 4.42, 4.4, 4.40, 4.41 | 1.20E+13 | 28 | 42 | O | [3] |
| slr1270 | Periplasmic protein, function unknown | 3, 252, 284, 285 | 57735.4 | 4.81 | 4.85, 4.63, 4.63, 4.68 | 9.57E+06 | 13 | 34 | P, O | [3],[8] |
| slr1272 | Probable porin; major outer membrane protein | 277, 299, 300 | 28029.6 | 4.83 | 4.76, 4.76, 4.88 | 1.03E+03 | 5 | 24 | O | [3] |
| slr1274 | Membrane protein | 16, 111 | 40879.8 | 4.51 | 4.7, 4.75 | 4.76E+06 | 17 | 37 | P | [8] |
| slr1275 | PilN | 110 | 30034.2 | 4.45 | 4.59 | 1.75E+03 | 7 | 36 | P | [8] |
| slr1276 | PilO | 36 | 30067.1 | 4.22 | 4.22 | 5.42E+04 | 8 | 24 | P | [8] |
| slr1277 | General secretion pathway protein D | 96, 190, 251, 282, 283 | 84642.3 | 4.64 | 4.81, 4.58, 4.6, 4.60, 4.63 | 2.12E+11 | 23 | 45 | O | [3] |
| slr1280 | NADH-ubiquinone oxidoreductase subunit PsbG | 39 | 27345.7 | 8.31 | 9.15 | 3.18E+05 | 17 | 54 | T | [5] |
| slr1281 | NADH dehydrogenase subunit I | 127, 128, 205 | 18286.9 | 4.65 | 4.56, 4.63, 4.47 | 3.62E+04 | 11 | 48 | T | [5] |
| slr1295 | Iron transport protein | 199 | 39370.4 | 4.87 | 4.7 | 1.11E+05 | 14 | 38 | P, O,T | [3],[5],[8] |
| slr1329 | ATP synthase b subunit | 4, 5, 6, 7, 8, 90, 91, 99, 100, 103, 173, 174 | 51733.3 | 4.89 | 4.98, 5.15, 5.20, 5.56, 5.64, 5.15, 5.20, 5.56, 5.64, 5.19, 5.42, 5.51 | 3.41E+13 | 27 | 63 | T | [5] |
| slr1330 | ATPase subunit epsilon | 56, 72, 138, 139, 240, 242, 310 | 14580.6 | 5.2 | 5.50, 5.76, 5.76, 5.50, 5.09, 5.31, 5.58 | 3.98E+04 | 6 | 49 | T | [5] |
| slr1347 | Carbonic anhydrase | 123, 224 | 30761 | 5.8 | 6.54, 5.75 | 1.74E+05 | 10 | 43 |  |  |
| slr1356 | 30S ribosomal protein S1 | 112, 202 | 36570.2 | 4.57 | 4.81, 4.58 | 1.22E+06 | 16 | 40 |  |  |
| slr1416 | MorR protein | 163 | 35091.8 | 5.06 | 5.47 | 1.30E+06 | 10 | 28 |  |  |
| slr1506 | Hypothetical protein | 255, 256, 257, 258, 291 | 68847.7 | 5.16 | 5.16, 5.21, 5.27, 5.32, 5.18 | 3.44E+09 | 18 | 45 | P, O | [3],[8] |
| slr1577 | Hypothetical protein | 147 | 18231.9 | 4.98 | 4.75 | 1.37E+04 | 5 | 30 |  |  |
| slr1623 | Ndh subunit | 133, 134, 208, 209 | 14077.7 | 4.56 | 4.56, 4.68, 4.48, 4.69 | 1.95E+04 | 8 | 47 | T | [5] |
| slr1839 | Carbon dioxide concentrating mechanism protein CcmK | 141 | 11902.7 | 5.79 | 6.51 | 1.47E+03 | 8 | 47 |  |  |
| slr1841 | Probable porin | 2, 97, 98, 191, 192, 193 | 67601.3 | 4.47 | 4.7, 4.52, 4.60, 4.35, 4.40, 4.45 | 3.71E+05 | 12 | 22 | P, O | [3],[4],[8] |
| slr1908 | Probable porin | 253, 286, 287, 288, 289 | 64511.2 | 5.07 | 4.83, 4.77, 4.83, 4.90, 5.00 | 1.26E+06 | 17 | 32 | P, O | [3],[4],[8] |
| slr1909 | NarL subfamily | 182, 231 | 24046.5 | 5.33 | 5.9, 5.39 | 1.10E+03 | 5 | 52 |  |  |
| slr1986 | Allophycocyanin beta subunit | 61, 239 | 17215.7 | 5.43 | 4.7, 5.37 | 472 | 4 | 20 |  |  |
| slr2024 | CheY subfamily | 47, 48 | 20232.9 | 6.34 | 7.50, 6.72 | 1.77E+04 | 7 | 53 |  |  |
| slr2034 | Putative homolog of plant HCF136 | 198 | 37291.1 | 4.76 | 4.62 | 2.11E+03 | 9 | 29 |  |  |
| slr2067 | Allophycocyanin alpha subunit | 60 | 17354.8 | 4.75 | 4.9 | 5.24E+04 | 9 | 55 |  |  |
| ssl0563 | Photosystem I subunit PsaC | 82, 83 | 8828.3 | 5.65 | 5.25, 6.45 | 44 | 2 | 22 | P,T | [5],[8] |
| ssl0707 | PII protein | 77, 78, 79, 140, 184, 243, 244, 247, 309 | 12397.4 | 6.33 | 7.15, 6.78, 6.07, 6.07, 5.90, 5.52, 5.69, 5.41, 4.79 | 2.65E+04 | 10 | 63 | P | [8] |
| ssl1690 | NdhO | 84 | 8288.6 | 6.56 | 8.5 | 3.41E+03 | 6 | 68 | T | [5] |
| ssl3093 | Phycocyanin associated linker protein | 81 | 9322.4 | 9.39 | 9.3 | 6.03E+04 | 7 |  |  |  |
| ssr2831 | Photosystem I subunit PsaE | 86, 87, 185, 245 | 8145.2 | 8.03 | 9.15, 8.03, 5.68, 5.24 | 341 | 5 | 82 | T | [5] |
| ssr2998 | Cytochrome b6f associated protein | 88 | 7220.4 | 8.03 | 9 | 1.14E+03 | 5 | 78 |  |  |
|  |  |  |  |  |  |  |  |  |  |  |
| **The proteins below were identified by Wang et. al. [4]** | | | | | |  |  |  |  |  |
| sll0057 | Heat shock protein GrpE |  | 27567.7 | 4.62 | 4.4 | 901 | 6 | 29 |  |  |
| sll0503 | Hypothetical protein |  | 51159.2 | 4.99 | 4.52 | 249 | 4 | 13 |  |  |
| sll1184 | Heme oxygenase |  | 27050.8 | 6.24 | 6.33 | 4.71E+04 | 8 | 39 |  |  |
| sll1284 | Serine esterase |  | 22209.7 | 5.08 | 5.36 | 1.05E+03 | 4 | 31 |  |  |
| sll1423 | Global nitrogen regulator |  | 25046.3 | 6.53 | 7.2 | 6.72E+03 | 6 | 38 |  |  |
| slr0075 | ABC transporter subunit |  | 28419.6 | 4.76 | 4.62 | 247 | 4 | 31 |  |  |
| slr0172 | IMP dehydrogenase |  | 17270.1 | 5.18 | 5.34 | 4.81E+05 | 7 | 45 |  |  |
| slr0455 | Hypothetical protein |  | 15790.8 | 5.05 | 4.65 | 1.30E+03 | 5 | 36 |  |  |
| slr1254 | Phytoene dehydrogenase |  | 52921.1 | 5.13 | 5.3 | 353 | 5 | 14 | T | [5],[6] |
| slr1350 | ATPase subunit epsilon |  | 14580.6 | 5.2 | 5.61 | 1.78E+05 | 8 | 50 |  |  |
| slr1668 | periplasmic protein, function unknown (target gene of sycrp1) |  | 30633.4 | 5.97 | 5.4 | 1.50E+03 | 4 | 18 |  |  |
| ssl0352 | Hypothetical protein |  | 6577.5 | 5.13 | 5.16 | 1.82E+03 | 5 | 65 |  |  |
| ssl0707 | PII protein |  | 12397.4 | 6.33 | 5.06, 5.7, 5.97 | 5.88E+03 | 6 | 48 |  |  |
| ssl2501 | Unknown protein |  | 10648.1 | 4.61 | 4.08 | 2.09E+05 | 8 | 64 | T | [5],[6] |
| ssl2781 | Hypothetical protein |  | 9655.2 | 5.86 | 6.38 | 1.76E+04 | 6 | 90 |  |  |
| ssr3532 | Unknown protein |  | 9170.6 | 5.23 | 4.62 | 71 | 4 | 35 |  |  |
| In this column, * represents the proteins that were identified for the first time in the current work. | | | | | |  |  |  |  |  |
| ^†^In this column, O, P, and T represent the our membrane, and the thylakoid membrane respectively | | |  |  |  |  |  |  |  |  |
