# Supplementary material for: Identification and bioinformatic analysis of the membrane proteins of synechocystis sp. PCC 6803
Source: Proteome Sci. 2009 Mar 25;7:11. doi: 10.1186/1477-5956-7-11 (PMC2666656; doi:10.1186/1477-5956-7-11)
Supplement: Additional file 2 — Transmembrane domain prediction of the identified proteins in the integral fractions. Additional file 2 is a MS word table containing the proteins with their predicted position of transmembrane domain, score of hydrophobicity, and size using TopPred. [file 1477-5956-7-11-S2.doc]

| **Additional file 2. Transmembrane domain prediction of the identified proteins in the integral fractions** | | | |
| --- | --- | --- | --- |
| **Protein** | **Position of TM** | **Score for hydrophobicity*** | **Size of protein (aa)** |
| sll0224 | 4 - 24 | 1.69 | 298 |
| sll0772 | 1 - 21 | 1.614 | 546 |
|  | 472 - 492 | 1.079 |  |
| sll1306 | 6 - 26 | 1.094 | 335 |
| sll1307 | 4 - 24 | 1.863 | 175 |
|  | 122 - 142 | 1.263 |  |
| sll1307 | 4 - 24 | 1.863 | 175 |
|  | 122 - 142 | 1.263 |  |
| sll1316 | 18 - 38 | 1.612 | 180 |
| sll1338 | 5 - 25 | 1.738 | 187 |
| sll1358 | 13 - 33 | 2.295 | 394 |
| sll1450 | 11 - 31 | 1.569 | 446 |
|  | 119 - 139 | 1.151 |  |
| sll1581 | 11 - 31 | 1.54 | 504 |
|  | 483 - 503 | 1.085 |  |
| sll1835 | 16 - 36 | 1.985 | 265 |
| slr0013 | 19 - 39 | 2.35 | 175 |
| slr0151 | 30 - 50 | 1.767 | 320 |
| slr0431 | 20 - 40 | 1.579 | 250 |
| slr0891 | 6 - 26 | 2.052 | 591 |
|  | 42 - 62 | 1.315 |  |
|  | 107 - 127 | 1.019 |  |
|  | 133 - 153 | 1.08 |  |
| slr1053 | 1 - 21 | 1.788 | 267 |
| slr1270 | 12 - 32 | 1.957 | 526 |
| slr1272 | 8 - 28 | 1.785 | 254 |
| slr1277 | 10 - 30 | 1.05 | 785 |
|  | 512 - 532 | 1.175 |  |
|  | 14 - 34 | 1.081 |  |
|  | 498 - 518 | 1.476 |  |
| slr1506 | 11 - 31 | 1.121 | 622 |
|  | 337 - 357 | 1.218 |  |
|  | 491 - 511 | 1.163 |  |
| slr1908 | 11 - 31 | 1.534 | 591 |
|  | 513 - 533 | 1.246 |  |
| *The hydrophobicity scores delineate the certainty of the TM prediction. A score that is higher or equal to 1.0 indicates that the prediction is **certain**; A score that is higher than 0.6, but smaller than 1.0 indicates that the prediction is **putative**. Only TMs with high prediction certainty (certain) were included in this table. | | | |
