# Supplementary material for: Identification and bioinformatic analysis of the membrane proteins of synechocystis sp. PCC 6803
Source: Proteome Sci. 2009 Mar 25;7:11. doi: 10.1186/1477-5956-7-11 (PMC2666656; doi:10.1186/1477-5956-7-11)
Supplement: Additional file 3 — Functional domain prediction of the hypothetical proteins. Additional file 3 is a MS word table containing the functional domain and motif analysis of 12 hypothetical proteins identified in this study using the computational software InterProScan. [file 1477-5956-7-11-S3.doc]

| **Additional file 3. Functional domain prediction of the hypothetical proteins** | | | |
| --- | --- | --- | --- |
| **Protein** | **Profile** | **Region** | **Type** |
| sll1118 | Protein of unknown function DUF74 | 1-108 | Family |
| sll1306 | Polysaccharide deacetylase | 20-165 | Family |
| sll1835 | Curli production assembly/transport component CsgG | 2-242 | Family |
| slr0038 | Mitochondrial energy transfer proteins(carrier protein) | 9-18 | Family |
| slr0151 | RNA-binding region RNP-1 (RNA recognition motif) | 165-172 | Domain |
|  | TPR repeat | 125-192 | Repeat |
| slr0244 | Usp domain | 1-132 | Domain |
|  |  | 139-280 |  |
| slr0362 | HAD-superfamily hydrolase, subfamily IIIA | 41-152 | Family |
| slr0670 | Usp domain | 1-134 | Domain |
| slr0848 | ATP synthase B/B' CF (0) | 17-71 | Family |
| slr1034 | Single-strand binding protein | 1-100 | Family |
| slr1270 | Outer membrane efflux protein | 103-318 | Family |
|  |  | 330-525 |  |
| slr1506 | Esterase/lipase/thioesterase | 232-343 | Family |
|  | ATP/GTP-binding site motif A | 39341 | Domain |
