# Supplementary material for: Identification and bioinformatic analysis of the membrane proteins of synechocystis sp. PCC 6803
Source: Proteome Sci. 2009 Mar 25;7:11. doi: 10.1186/1477-5956-7-11 (PMC2666656; doi:10.1186/1477-5956-7-11)
Supplement: Additional file 4 — The proteins of synechocystis sp. PCC 6803 identified through large-scale proteomic approaches. Additional file 4 is a MS word table containing 1,738 proteins identified based on the references mentioned in the text with their subcellular locations determined by either experiments or predicted by TopPred. [file 1477-5956-7-11-S4.doc]

| **Additional file 4. The proteins of *Synechocystis* sp. PCC 6803 identified through large-scale proteomic approaches** | | | |
| --- | --- | --- | --- |
| **ORF** | **Gene Product** | **Subcellular Locations^†^** | **References^‡^** |
| sll0002 | penicillin-binding protein | PM | [67] |
| sll0005 | hypothetical protein | PS | [66], [67] |
| sll0006 | putative aminotransferase | PM | [67] |
| sll0016 | probable membrane-bound lytic transglycosylase A | PM | [66], [67] |
| sll0017 | glutamate-1-semialdehyde aminomutase | PM | [63], [64], [65], [66], [67] |
| sll0018 | fructose-1,6-bisphosphate aldolase | S, T | [27], [28], [56], [60], [62], [63], [64], [65], [66], [67] |
| sll0019 | 1-deoxy-d-xylulose 5-phosphate reductoisomerase | PM | [63], [64], [66], [67] |
| sll0020 | ATP-dependent Clp protease regulatory subunit | M, P, S, T | [C], [5], [10], [59], [62], [63], [64], [65], [66], [67] |
| sll0023 | hypothetical protein | PS | [67] |
| sll0033 | carotene isomerase | PM | [67] |
| sll0034 | putative carboxypeptidase | P | [4], [8], [10], [66], [67] |
| sll0036 | hypothetical protein | PM | [66], [67] |
| sll0037 | hypothetical protein | PS | [67] |
| sll0038 | positive phototaxis protein, two-component response regulator PatA subfamily | PS | [67] |
| sll0041 | methyl-accepting chemotaxis protein, pixJ1 | P | [4], [67] |
| sll0043 | positive phototaxis protein, homologous to chemotaxis protein CheA, two-component hybrid histidine kinase | PM | [66], [67] |
| sll0044 | unknown protein | PS | [67] |
| sll0045 | sucrose phosphate synthase | PM | [67] |
| sll0048 | unknown protein | PM | [64], [66], [67] |
| sll0051 | hypothetical protein | PS | [63], [65], [66], [67] |
| sll0053 | biotin carboxylase | PS | [66], [67] |
| sll0055 | processing protease | PS | [67] |
| sll0057 | heat shock protein GrpE | M, S | [6], [61], [63], [64], [65], [66], [67] |
| sll0058 | DnaK protein 1, heat shock protein 70, molecular chaperone | PM | [64], [67] |
| sll0064 | amino-acid ABC transporter binding proteinH | PP | [55], [63], [64], [67] |
| sll0065 | acetolactate synthase, small subunit | M | [C], [59], [64], [67] |
| sll0066 | unknown protein | PM | [67] |
| sll0067 | glutathione S-transferase | PM | [67] |
| sll0068 | unknown protein | PM | [66], [67] |
| sll0069 | hypothetical protein | S | [62], [67] |
| sll0070 | phosphoribosylglycinamide formyltransferase | PS | [67] |
| sll0071 | hypothetical protein | PS | [66], [67] |
| sll0078 | threonyl-tRNA synthetase | PS | [63], [66], [67] |
| sll0080 | N-acetyl-gamma-glutamyl-phosphate reductase | PM | [63], [65], [66], [67] |
| sll0083 | phosphoheptose isomerase | PS | [67] |
| sll0085 | unknown protein | PS | [66] |
| sll0086 | oxyanion-translocating ATPase, ArsA | M | [59], [64], [67] |
| sll0096 | hypothetical protein | PS | [66], [67] |
| sll0098 | hypothetical protein | PS | [67] |
| sll0100 | N-acyl-L-amino acid amidohydrolase | S | [27], [28] |
| sll0102 | hypothetical protein | PS | [64], [66], [67] |
| sll0103 | hypothetical protein | S | [60], [62], [64], [66], [67] |
| sll0108 | ammonium/methylammonium permease | PM | [67] |
| sll0135 | putative 5'-methylthioadenosine phosphorylase | PS | [66], [67] |
| sll0136 | aminopeptidase P | PS | [66], [67] |
| sll0141 | membrane fusion protein | P | [8], [67] |
| sll0142 | probable cation efflux system protein | PM | [66], [67] |
| sll0144 | uridine monophosphate kinase | PS | [64], [67] |
| sll0145 | ribosome releasing factor | PS | [27], [28], [63], [64], [65], [67] |
| sll0147 | hypothetical protein | PS | [66], [67] |
| sll0148 | hypothetical protein | PM | [66], [67] |
| sll0149 | hypothetical protein | PM | [66], [67] |
| sll0154 | hypothetical protein | PM | [66] |
| sll0158 | 1,4-alpha-glucan branching enzyme | PM | [63], [64], [65], [66], [67] |
| sll0160 | hypothetical protein | PM | [67] |
| sll0162 | hypothetical protein | PS | [67] |
| sll0163 | WD-repeat protein | PM | [66], [67] |
| sll0166 | a fusion protein between uroporphyrinogen-III C-methyltransferase (CobA/CorA) and uroporphyrinogen-III synthase (HemD) | PM | [66], [67] |
| sll0167 | unknown protein | PP | [56] |
| sll0169 | cell division protein Ftn2 homolog | PM | [67] |
| sll0170 | DnaK protein 2, hsp 70 | C, S, T | [5], [56], [60], [61], [62], [63], [64], [65], [66], [67] |
| sll0171 | probable aminomethyltransferase | PS | [63], [67] |
| sll0172 | hypothetical protein | PP | [27], [28], [55], [56], [66] |
| sll0173 | virginiamycin b hydrolase | O, PP | [3], [55], [63], [64], [67] |
| sll0175 | hypothetical protein | PM | [67] |
| sll0178 | hypothetical protein | PM | [66] |
| sll0179 | glutamyl-tRNA synthetase | S | [60], [63], [64], [66], [67] |
| sll0180 | membrane fusion protein | O, P | [3], [4], [8], [10], [67] |
| sll0182 | ABC transporter ATP-binding protein | PM | [67] |
| sll0184 | group2 RNA polymerase sigma factor SigC | PS | [67] |
| sll0185 | hypothetical protein | PM | [66], [67] |
| sll0188 | unknown protein | PM | [66], [67] |
| sll0195 | probable ATP-dependent protease | PS | [66], [67] |
| sll0199 | plastocyanin precursor | S | [27], [28], [60], [63], [64], [65], [66], [67] |
| sll0202 | glucose inhibited division protein | PM | [66], [67] |
| sll0204 | glucose inhibited division protein | PM | [67] |
| sll0207 | glucose-1-phosphate thymidylyltransferase | PM | [67] |
| sll0208 | hypothetical protein | PM | [67] |
| sll0209 | hypothetical protein | PS | [67] |
| sll0210 | bacitracin resistance protein | PM | [67] |
| sll0216 | hypothetical protein | PS | [67] |
| sll0220 | L-glutamine:D-fructose-6-P amidotransferase | PM | [63], [64], [66], [67] |
| sll0224 | amino-acid ABC transporter binding protein | M | [C], [64], [67] |
| sll0226 | photosystem I assembly related protein | PM | [67] |
| sll0227 | peptidyl-prolyl cis-trans | P, PP | [10], [55], [56], [63], [67] |
| sll0228 | arginase | PS | [67] |
| sll0230 | hypothetical protein | PM | [27], [28], [63] |
| sll0236 | unknown protein | PS | [67] |
| sll0240 | ABC transporter ATP-binding protein | PS | [67] |
| sll0242 | unknown protein | PM | [66] |
| sll0244 | UDP-glucose 4-epimerase | PM | [64], [67] |
| sll0245 | putative GTP-binding protein | M, S | [59], [61], [63], [64], [65], [66], [67] |
| sll0247 | Iron-stress induced Chl-binding protein CP43 | M | [58], [67] |
| sll0248 | flavodoxin | PS | [64], [67] |
| sll0250 | pantothenate metabolism flavoprotein | PM | [67] |
| sll0252 | unknown protein | PM | [66], [67] |
| sll0254 | probable phytoene dehydrogenase Rieske iron-sulfur component | PM | [67] |
| sll0258 | cytochrome c550 | S | [27], [28], [61], [63], [64], [65], [66], [67] |
| sll0260 | hypothetical protein | PM | [67] |
| sll0262 | acyl-lipid desaturase (delta 6) | PM | [67] |
| sll0267 | unknown protein | PM | [66], [67] |
| sll0270 | primosomal protein N | S | [61] |
| sll0271 | N utilization substance protein B homolog | PS | [66], [67] |
| sll0272 | hypothetical protein | M | [C], [6], [27], [28], [63], [65], [67] |
| sll0273 | Na+/H+ antiporter | PM | [67] |
| sll0274 | hypothetical protein | PM | [27], [28], [63] |
| sll0283 | hypothetical protein | PM | [67] |
| sll0289 | septum site-determining protein MinD | M | [C], [64], [67] |
| sll0290 | polyphosphate kinase | PS | [66], [67] |
| sll0293 | unknown protein | PP | [56], [66] |
| sll0301 | hypothetical protein | PM | [66] |
| sll0306 | RNA polymerase group 2 sigma factor | PS | [66] |
| sll0312 | probable oligopeptides ABC transporter permease protein | PM | [67] |
| sll0314 | hypothetical protein | P, PP | [10], [27], [28], [55], [64], [66], [67] |
| sll0318 | hypothetical protein | PM | [67] |
| sll0319 | hypothetical protein | O, PP | [3], [27], [28], [55], [56], [67] |
| sll0320 | probable ribonuclease D | PS | [63], [64], [67] |
| sll0322 | putative hydrogenase expression/formation protein HypF | PS | [66] |
| sll0325 | hypothetical protein | P | [10], [67] |
| sll0329 | 6-Phosphogluconate dehydrogenase, decarboxylating (gnd) | S | [60], [61], [62], [63], [64], [65], [66], [67] |
| sll0335 | hypothetical protein | PM | [64], [66], [67] |
| sll0336 | acetyl-CoA carboxylase beta subunit | PM | [67] |
| sll0337 | phosphate sensor, two-component sensor histidine kinase | PS | [67] |
| sll0350 | hypothetical protein | PM | [66] |
| sll0356 | N-(5'-phosphoribosyl)anthranilate isomerase | PM | [66] |
| sll0359 | hypothetical protein | PS | [27], [28], [63], [65], [66], [67] |
| sll0362 | alanyl-tRNA synthetase | PM | [66], [67] |
| sll0368 | pyrimidine operon regulatory protein PyrR | M | [C], [63], [65], [67] |
| sll0370 | carbamoyl-phosphate synthase, pyrimidine-specific, large chain | PM | [66], [67] |
| sll0373 | gamma-glutamyl phosphate reductase | PS | [64], [66], [67] |
| sll0374 | urea transport system ATP-binding protein | PM | [67] |
| sll0376 | unknown protein | PM | [67] |
| sll0377 | transcription-repair coupling factor | PS | [66], [67] |
| sll0378 | uroporphyrin-III C-methyltransferase | PM | [66], [67] |
| sll0379 | acyl-[acyl-carrier-protein]--UDP-N-acetylglucosamine o-acyltransferase | PS | [66], [67] |
| sll0380 | probable glycosyltransferase | PM | [66] |
| sll0381 | hypothetical protein | PM | [66], [67] |
| sll0384 | unknown protein | PM | [67] |
| sll0395 | phosphoglycerate mutase | PS | [64], [66], [67] |
| sll0396 | two-component response regulator OmpR subfamily | PS | [67] |
| sll0401 | citrate synthase | PM | [63], [64], [65], [66], [67] |
| sll0402 | aspartate aminotransferase | PS | [64], [66], [67] |
| sll0404 | glycolate oxidase subunit GlcD | PS | [67] |
| sll0405 | unknown protein | PS | [66] |
| sll0408 | hypothetical protein - peptidylprolyl cis-trans isomerase | M, S, T | [C], [5], [60], [61], [62], [63], [64], [65], [66], [67] |
| sll0410 | hypothetical protein | PS | [67] |
| sll0412 | hypothetical protein | T | [5], [66], [67] |
| sll0413 | hypothetical protein | PS | [67] |
| sll0414 | hypothetical protein | PS | [67] |
| sll0415 | ATP-binding protein of ABC transporter | PM | [67] |
| sll0416 | GroEL protein 2 | C, P, S, T | [5], [8], [10], [27], [28], [56], [60], [61], [62], [63], [64], [65], [66], [67] |
| sll0418 | 2-methyl-6-phytylbenzoquinone methyltransferase | PM | [67] |
| sll0420 | urease beta subunit | S | [27], [28] |
| sll0421 | adenylosuccinate lyase | PS | [63], [64], [65], [66], [67] |
| sll0422 | asparaginase | PS | [27], [28], [67] |
| sll0424 | hypothetical protein | PM | [66] |
| sll0427 | PSII subunit PsbO | M, P, S, T | [C], [5], [6], [8], [10], [27], [28], [60], [62], [63], [64], [65], [66], [67] |
| sll0430 | HtpG, hsp 90 | S | [62], [64], [66], [67] |
| sll0441 | unknown protein | PS | [66] |
| sll0443 | hypothetical protein | P | [10], [67] |
| sll0445 | unknown protein | PM | [67] |
| sll0446 | unknown protein | PM | [63], [66], [67] |
| sll0454 | phenylalanyl-tRNA synthetase alpha chain | PS | [64], [67] |
| sll0455 | homoserine dehydrogenase | PM | [64], [67] |
| sll0456 | hypothetical protein | PS | [67] |
| sll0459 | excinuclease ABC subunit B | PM | [67] |
| sll0461 | gamma-glutamyl phosphate reductase | PS | [63], [66], [67] |
| sll0467 | S-adenosylmethionine:tRNA ribosyltransferase-isomerase | PS | [67] |
| sll0469 | ribose-phosphate pyrophosphokinase | PS | [66], [67] |
| sll0470 | hypothetical protein | PM | [63], [66], [67] |
| sll0471 | hypothetical protein | PM | [66] |
| sll0474 | two-component hybrid sensor and regulator | PM | [66] |
| sll0477 | putative biopolymer transport ExbB-like protein | PM | [66], [67] |
| sll0480 | hypothetical protein | S | [60], [63], [64], [66], [67] |
| sll0482 | unknown protein | PM | [67] |
| sll0487 | hypothetical protein | PS | [67] |
| sll0489 | ATP-binding protein of ABC transporter | PS | [67] |
| sll0493 | hypothetical protein | PS | [67] |
| sll0495 | asparaginyl-tRNA synthetase | PM | [66], [67] |
| sll0497 | hypothetical protein | PS | [64], [67] |
| sll0499 | hypothetical protein | PM | [66], [67] |
| sll0501 | probable glycosyltransferase | PM | [67] |
| sll0502 | arginyl-tRNA-synthetase | PS | [66], [67] |
| sll0503 | hypothetical protein | M | [6] |
| sll0504 | diaminopimelate decarboxylase | PS | [67] |
| sll0505 | hypothetical protein | PM | [66] |
| sll0506 | undecaprenyl pyrophosphate synthetase | PS | [67] |
| sll0507 | probable cation transporter | PM | [67] |
| sll0509 | similar to 5',5'''-P-1,P-4-tetraphosphate phosphorylase II | PS | [66] |
| sll0513 | hypothetical protein | PM | [67] |
| sll0514 | hypothetical protein | PM | [67] |
| sll0518 | unknown protein | PM | [67] |
| sll0519 | NADH dehydrogenase subunit 1 | PM | [67] |
| sll0520 | NADH dehydrogenase subunit, NdhI | M, T | [5], [58], [67] |
| sll0521 | NADH dehydrogenase subunit 6 | PM | [67] |
| sll0524 | hypothetical protein | PM | [66], [67] |
| sll0528 | hypothetical protein | PM | [67] |
| sll0529 | hypothetical protein | S | [60], [63], [64], [65], [66], [67] |
| sll0533 | trigger factor | S | [61], [63], [64], [65], [66], [67] |
| sll0534 | ATP-dependent Clp protease subunits | S | [60], [67] |
| sll0535 | ATP-dependent Clp protease ATPase subunit | PS | [67] |
| sll0539 | hypothetical protein | S | [61] |
| sll0540 | phosphate-binding protein PstS homolog | PM | [66] |
| sll0541 | acyl-lipid desaturase (delta 9) | PM | [67] |
| sll0542 | acetyl-coenzyme A synthetase | PS | [63], [64], [66], [67] |
| sll0545 | hypothetical protein | PM | [67] |
| sll0550 | flavoprotein | S | [60], [63], [64], [65], [66], [67] |
| sll0553 | hypothetical protein | PM | [64], [66], [67] |
| sll0554 | ferredoxin-thioredoxin reductase, catalytic chain | PS | [63] |
| sll0555 | methionine aminopeptidase | PS | [67] |
| sll0556 | Na+/H+ antiporter | PM | [66] |
| sll0558 | hypothetical protein YCF53 | PS | [67] |
| sll0563 | unknown protein | PS | [66] |
| sll0565 | hypothetical protein | P | [8], [10] |
| sll0567 | ferric uptake regulation protein | PS | [67] |
| sll0569 | RecA | M | [59], [66], [67] |
| sll0572 | hypothetical protein | PS | [66], [67] |
| sll0573 | carbamate kinase | PM | [66], [67] |
| sll0574 | probable permease protein of lipopolysaccharide ABC transporter | PM | [67] |
| sll0575 | probable lipopolysaccharide ABC transporter ATP binding subunit | PS | [66] |
| sll0576 | putative sugar-nucleotide epimerase/dehydratease | PM | [63], [65], [66], [67] |
| sll0578 | phosphoribosylaminoimidazole carboxylase ATPase subunit | PM | [67] |
| sll0585 | hypothetical protein | PS | [67] |
| sll0587 | pyruvate kinase | PM | [63], [67] |
| sll0588 | unknown protein | PM | [63], [65] |
| sll0593 | glucokinase | PM | [63], [64], [66], [67] |
| sll0594 | transcriptional regulator | PS | [66] |
| sll0596 | hypothetical protein | PM | [63], [64], [67] |
| sll0601 | nitrilase homolog | PS | [27], [28], [64], [67] |
| sll0602 | hypothetical protein | PM | [66], [67] |
| sll0606 | ABC transporter; branched chain aa binding protein | P | [8], [10], [67] |
| sll0615 | hypothetical protein | PM | [67] |
| sll0616 | preprotein translocase SecA | M, S, T | [5], [59], [62], [63], [64], [66], [67] |
| sll0617 | Vipp1 | M, P, T | [C], [5], [8], [10], [27], [28], [63], [64], [65], [66], [67] |
| sll0622 | quinolinate synthetase | PS | [67] |
| sll0625 | unknown protein | PS | [64], [67] |
| sll0626 | putative neutral invertase | PM | [67] |
| sll0630 | unknown protein | S | [27], [28] |
| sll0631 | L-aspartate oxidase | PM | [64], [66], [67] |
| sll0634 | photosystem I biogenesis protein BtpA | PS | [66], [67] |
| sll0635 | thiamine-phosphate pyrophosphorylase (thiE) | S | [62], [67] |
| sll0638 | hypothetical protein | PP | [55], [67] |
| sll0639 | hypothetical protein | PS | [66], [67] |
| sll0644 | probable esterase | PM | [66] |
| sll0645 | unknown protein | PM | [67] |
| sll0646 | guanylyl cyclase | PM | [67] |
| sll0654 | alkaline phosphatase | PM | [66] |
| sll0657 | phospho-N-acetylmuramoyl-pentapeptide-transferase | PM | [67] |
| sll0659 | hypothetical protein | PM | [67] |
| sll0660 | pyridoxal phosphate biosynthetic protein PdxA | PS | [67] |
| sll0664 | unknown protein | PS | [67] |
| sll0672 | cation-transporting p-type ATPase PacL | PM | [66], [67] |
| sll0679 | periplasmic phosphate-binding protein of ABC transporter | PM | [66] |
| sll0680 | ABC transporter; Phosphate binding protein | M, P | [C], [10], [63], [65], [66], [67] |
| sll0681 | phosphate transport system permease protein PstC homolog | PM | [67] |
| sll0683 | phosphate transport ATP-binding protein PstB | M | [C], [63], [66], [67] |
| sll0684 | phosphate transport ATP-binding protein PstB | M | [C] |
| sll0689 | Na+/H+ antiporter | PM | [66], [67] |
| sll0698 | drug sensory protein A, low temperature sensor, two-component sensor histidine kinase | PM | [67] |
| sll0703 | unknown protein | PS | [67] |
| sll0707 | PII protein | M | [6] |
| sll0708 | dimethyladenosine transferase | PS | [66], [67] |
| sll0709 | putative endonuclease | PS | [66] |
| sll0711 | isopentenyl monophosphate kinase | PM | [66], [67] |
| sll0712 | cysteine synthase | PM | [27], [28], [63], [66], [67] |
| sll0716 | leader peptidase I, signal peptidase I | T | [5], [67] |
| sll0721 | unknown protein | PS | [67] |
| sll0723 | unknown protein | PM | [66] |
| sll0726 | phosphoglucomutase | PS | [63], [64], [65], [66], [67] |
| sll0728 | acetyl-CoA carboxylase alpha subunit | PM | [66], [67] |
| sll0729 | probable DNA methyltransferase | PS | [67] |
| sll0735 | hypothetical protein | PS | [63], [64], [66], [67] |
| sll0737 | hypothetical protein | PM | [67] |
| sll0738 | molybdate-binding periplasmic protein | PP | [56] |
| sll0739 | ATP-binding protein of molybdate ABC transporter | PM | [66] |
| sll0740 | hypothetical protein | PS | [66] |
| sll0741 | pyruvate flavodoxin oxidoreductase | PM | [66], [67] |
| sll0745 | phosphofructokinase | PS | [67] |
| sll0749 | hypothetical protein | P | [8], [10], [67] |
| sll0750 | two-component sensor histidine kinase, KaiC-interacting protein, involved in circadian rhythm | PS | [66], [67] |
| sll0751 | hypothetical protein YCF22 | PM | [67] |
| sll0752 | hypothetical protein | P | [4], [8], [66], [67] |
| sll0753 | FolD bifunctional protein | PM | [27], [28], [64], [66], [67] |
| sll0754 | ribosome binding factor A | PS | [27], [28], [64] |
| sll0755 | thioredoxin peroxidase | PS | [64], [67] |
| sll0756 | unknown protein | S | [27], [28] |
| sll0757 | amidophosphoribosyltransferase | PM | [67] |
| sll0759 | ABC transporter ATP-binding protein | PS | [67] |
| sll0764 | urea transporter, ATP-binding subunit (UrtD) | M | [59], [66], [67] |
| sll0765 | hypothetical protein | PS | [67] |
| sll0766 | DNA repair protein RadC | PS | [66] |
| sll0767 | 50S ribosomal protein L20 | PS | [67] |
| sll0771 | glucose transport protein | PM | [67] |
| sll0772 | probable porin; major outer membrane protein | M | [C], [67] |
| sll0779 | unknown protein | PM | [66] |
| sll0781 | hypothetical protein | PS | [63], [64], [65], [66] |
| sll0783 | unknown protein | PS | [67] |
| sll0790 | two-component sensor histidine kinase | PM | [66] |
| sll0792 | transcriptional repressor SmtB | M | [C] |
| sll0794 | cobalt-dependent transcriptional regulator | PM | [66] |
| sll0807 | pentose-5-phosphate 3-epimerase | S | [27], [28], [60], [61], [63], [64], [66], [67] |
| sll0813 | cytochrome c oxidase subunit II, CoxB | P | [4], [8], [10] |
| sll0814 | hypothetical protein | PM | [67] |
| sll0815 | unknown protein | PS | [67] |
| sll0816 | probable oxidoreductase | PM | [66], [67] |
| sll0817 | tRNA delta-2-isopentenylpyrophosphate (IPP) transferase | PS | [67] |
| sll0819 | PSI subunit PsaF | M, P, T | [5], [8], [10], [58], [67] |
| sll0821 | phytochrome-like protein | PM | [67] |
| sll0822 | hypothetical protein | PS | [27], [28], [63], [66], [67] |
| sll0825 | polyA polymerase | PS | [66] |
| sll0827 | hypothetical protein | PM | [67] |
| sll0828 | putative amidase | PM | [67] |
| sll0829 | methyltransferase | M | [C], [67] |
| sll0830 | elongation factor EF-G | PS | [66], [67] |
| sll0834 | low affinity sulfate transporter | PM | [67] |
| sll0837 | hypothetical protein | PP, S | [55], [60], [64], [67] |
| sll0838 | orotidine 5' monophosphate decarboxylase | PS | [64], [66], [67] |
| sll0842 | neopullulanase | PM | [67] |
| sll0844 | tRNA (5-methylaminomethyl-2-thiouridylate)-methyltransferase | PS | [67] |
| sll0848 | chromosomal replication initiator protein DnaA | PS | [66] |
| sll0849 | PSII D2 protein, PsbD | M | [58] |
| sll0851 | PSII CP43 protein | M | [58], [67] |
| sll0853 | hypothetical protein | PS | [67] |
| sll0854 | hypothetical protein | PS | [66], [67] |
| sll0860 | hypothetical protein | PS | [67] |
| sll0861 | hypothetical protein | PS | [66], [67] |
| sll0872 | unknown protein | PM | [63], [65] |
| sll0873 | carboxynorspermidine decarboxylase | PM | [64], [67] |
| sll0875 | hypothetical protein | PM | [67] |
| sll0876 | holliday junction DNA helicase RuvA | PM | [67] |
| sll0877 | hypothetical protein | PS | [66], [67] |
| sll0886 | hypothetical protein | PM | [27], [28], [63], [67] |
| sll0887 | hypothetical protein | S | [61], [64], [66], [67] |
| sll0891 | malate dehydrogenase | C, S | [56], [60], [66], [67] |
| sll0895 | CysQ protein homolog | PS | [67] |
| sll0897 | DnaJ protein, heat shock protein 40, molecular chaperone | PS | [63], [66], [67] |
| sll0898 | hypothetical protein | PS | [67] |
| sll0899 | UDP-N-acetylglucosamine pyrophosphorylase | PS | [64], [67] |
| sll0900 | ATP phosphoribosyltransferase | PS | [66], [67] |
| sll0901 | phosphoribosylaminoimidazole carboxylase | PM | [67] |
| sll0902 | ornithine carbamoyltransferase | PM | [63], [64], [66], [67] |
| sll0910 | unknown protein | PS | [66] |
| sll0912 | ABC transporter ATP binding protein | PS | [66], [67] |
| sll0914 | unknown protein | PM | [67] |
| sll0915 | protease pqqE | O, PP | [3], [55], [56], [67] |
| sll0920 | phosphoenolpyruvate carboxylase | PS | [63], [65], [66], [67] |
| sll0921 | two-component response regulator NarL subfamily | PS | [63], [67] |
| sll0923 | exopolysaccharide export protein, EpsB | P | [4], [67] |
| sll0925 | hypothetical protein | PS | [67] |
| sll0927 | S-Adenosylmethionine synthetase (metX) | S | [62], [63], [66], [67] |
| sll0928 | allophycocyanin-B | PS | [63], [65], [66], [67] |
| sll0931 | hypothetical protein | PM | [67] |
| sll0933 | hypothetical protein | PM | [67] |
| sll0934 | carboxysome formation protein CcmA | PS | [63], [64], [66], [67] |
| sll0936 | putative oxidoreductase | PM | [66], [67] |
| sll0938 | aspartate transaminase | PM | [67] |
| sll0945 | glycogen synthase | PS | [63], [64], [65], [66], [67] |
| sll0947 | light-repressed protein, LrtA | M, P | [C], [6], [8], [10], [63], [64], [65], [66], [67] |
| sll0982 | hypothetical protein | T | [5], [66] |
| sll0983 | hypothetical protein | PS | [66] |
| sll0985 | unknown protein | PM | [66] |
| sll0992 | putative esterase | PS | [66] |
| sll0996 | hypothetical protein | PS | [66], [67] |
| sll0997 | hypothetical protein | T | [5], [67] |
| sll0998 | LysR transcriptional regulator | M | [C], [67] |
| sll1002 | hypothetical protein YCF22 | PM | [66], [67] |
| sll1004 | hypothetical protein | PM | [67] |
| sll1005 | MazG protein homolog | PS | [66] |
| sll1009 | unknown protein | PM | [63] |
| sll1017 | ammonium/methylammonium permease | PM | [67] |
| sll1018 | dihydroorotase | PS | [67] |
| sll1019 | hydroxyacylglutathione hydrolase | PS | [67] |
| sll1021 | hypothetical protein | P | [4], [8], [10], [63], [67] |
| sll1023 | succinyl-CoA synthetase beta chain | PM | [66], [67] |
| sll1027 | NADH-dependent glutamate synthase small subunit | PM | [67] |
| sll1028 | CO2 concentrating mechanism protein | M, P, S | [C], [6], [8], [27], [28], [60], [63], [64], [65], [66], [67] |
| sll1029 | CO2 concentrating mechanism protein | M, P, S, T | [C], [5], [8], [27], [28], [60], [63], [64], [65], [66], [67] |
| sll1030 | carbon dioxide concentrating mechanism protein CcmL, putative carboxysome assembly protein | S | [27], [28] |
| sll1031 | CcmM | M, O, P, S | [3], [4], [59], [62], [63], [65], [66], [67] |
| sll1032 | carbon dioxide concentrating mechanism protein CcmN, putative carboxysome assembly protein | PM | [67] |
| sll1033 | probable protein phosphatase | PM | [64], [67] |
| sll1035 | uracil phosphoribosyltransferase | PS | [27], [28], [63], [64], [65], [66], [67] |
| sll1036 | hypothetical protein | PM | [67] |
| sll1037 | unknown protein | PM | [66] |
| sll1039 | hypothetical protein | PS | [67] |
| sll1040 | unknown protein | PM | [66] |
| sll1041 | ATP-binding protein of ABC transporter | P | [8] |
| sll1043 | polyribonucleotide nucleotidyl transferase, α-subunit | C, M, S, T | [56], [59], [60], [61],[56], [62], [63], [64], [65], [66], [67] |
| sll1049 | hypothetical protein | PS | [67] |
| sll1051 | phycocyanin alpha-subunit phycocyanobilin lyase | PS | [67] |
| sll1053 | membrane fusion protein MtrC | P | [8], [67] |
| sll1056 | isomerase | S | [61], [62], [64], [67] |
| sll1058 | dihydrodipicolinate reductase | PM | [63], [64], [65], [66], [67] |
| sll1060 | hypothetical protein | PM | [67] |
| sll1064 | hypothetical protein | PM | [67] |
| sll1069 | 3-oxoacyl-[acyl-carrier-protein] synthase II | PM | [64], [66], [67] |
| sll1070 | transketolase | S, T | [5], [56], [60], [61], [62], [63], [64], [65], [66], [67] |
| sll1071 | hypothetical protein | PM | [67] |
| sll1072 | hypothetical protein | PS | [66] |
| sll1074 | leucyl-tRNA synthetase | PS | [66], [67] |
| sll1076 | cation-transporting ATPase PacL | PM | [66] |
| sll1077 | agmatinase | PM | [66] |
| sll1080 | ABC transporter; Nitrate/sulfonate/bicarbonate binding protein | P | [10], [67] |
| sll1081 | ABC transport system permease protein | PM | [66] |
| sll1084 | hypothetical protein | PS | [66], [67] |
| sll1085 | glycerol-3-phosphate dehydrogenase | PS | [67] |
| sll1087 | similar to sodium/glucose cotransporter | PM | [67] |
| sll1089 | hypothetical protein | PP | [55], [56] |
| sll1091 | 43 kD geranylgeranyl reductase bacteriochlorophyll synthetase | T | [5], [66], [67] |
| sll1092 | hypothetical protein | PS | [66] |
| sll1096 | 30S ribosomal protein S12 | PS | [67] |
| sll1097 | 30S ribosomal protein S7 | PS | [63], [65], [66], [67] |
| sll1098 | elongation factor EF-G 2 (fusB, fus) | S | [62], [64], [66], [67] |
| sll1099 | EF-Tu | M, P, S, T | [C], [5], [6], [8], [10], [59], [60], [61], [62], [63], [64], [65], [66], [67] |
| sll1101 | 30S ribosomal protein S10 | PS | [63], [66], [67] |
| sll1106 | hypothetical protein | M | [59], [63], [66], [67] |
| sll1107 | type IV pilus biogenesis protein PilI homolog | PM | [67] |
| sll1108 | stationary-phase survival protein SurE homolog | PM | [67] |
| sll1109 | hypothetical protein | PS | [63], [67] |
| sll1110 | peptide chain release factor 1 | PS | [64], [67] |
| sll1112 | 3-dehydroquinate dehydratase | PM | [66] |
| sll1118 | hypothetical protein | M, P | [C], [6], [8], [10] |
| sll1120 | chromosome segregation protein SMC1 | PM | [67] |
| sll1121 | hypothetical protein | PM | [66], [67] |
| sll1124 | two-component sensor histidine kinase, phytochrome-like protein | PM | [66] |
| sll1127 | 1,4-dihydroxy-2-naphthoate synthase | PM | [66], [67] |
| sll1129 | 2-hydroxy-6-oxohepta-2,4-dienoate hydrolase | PM | [67] |
| sll1130 | unknown protein | PS | [27], [28], [63], [65], [66], [67] |
| sll1135 | unknown protein | PM | [66], [67] |
| sll1138 | hypothetical protein | PS | [66] |
| sll1143 | DNA helicase | S | [61], [66], [67] |
| sll1144 | hypothetical protein | PS | [67] |
| sll1151 | unknown protein | PM | [66], [67] |
| sll1159 | probable bacterioferritin comigratory protein | PS | [66], [67] |
| sll1161 | probable adenylate cyclase | PM | [66] |
| sll1165 | mismatch repair protein muts | S | [61], [67] |
| sll1172 | threonine synthase | PS | [63], [65], [66], [67] |
| sll1173 | hypothetical protein | PS | [67] |
| sll1178 | nodulation protein, probable carbamoyl transferase | P | [4], [66], [67] |
| sll1180 | toxin secretion ABC transporter ATP-binding protein, HlyB | P | [4], [67] |
| sll1181 | HlyD | P | [10], [66], [67] |
| sll1184 | Heme oxygenase 1 | M | [6], [59], [66], [67] |
| sll1185 | Coproporphyrinogen III oxidase, aerobic (oxygen-dependent) | S | [62], [63], [64], [65], [66], [67] |
| sll1188 | hypothetical protein | PS | [63], [65], [67] |
| sll1194 | PSII subunit PsbU | M | [C], [6], [27], [28], [63], [64], [65], [66], [67] |
| sll1196 | phosphofructokinase | M | [C], [64], [66], [67] |
| sll1198 | tRNA (guanine-N1)-methyltransferase | PS | [67] |
| sll1202 | iron(III) dicitrate-binding protein of ABC transporter | PP | [56] |
| sll1206 | ferric aerobactin receptor, FhuA homolog | PM | [66] |
| sll1209 | DNA ligase | PS | [67] |
| sll1212 | GDP-mannose 4,6-dehydratase | PS | [63], [64], [65], [66], [67] |
| sll1213 | GDP-fucose synthetase | PS | [63], [64], [65], [66], [67] |
| sll1214 | hypothetical protein YCF59 | PS | [67] |
| sll1218 | hypothetical protein YCF39 | PM | [67] |
| sll1220 | putative diaphorase subunit of the bidirectional hydrogenase | PS | [67] |
| sll1221 | diaphorase subunit of the bidirectional hydrogenase | PM | [66], [67] |
| sll1223 | diaphorase subunit of the bidirectional hydrogenase | PS | [67] |
| sll1224 | hydrogenase subunit of the bidirectional hydrogenase | PM | [67] |
| sll1226 | hydrogenase subunit of the bidirectional hydrogenase | PS | [63], [64], [66], [67] |
| sll1229 | two-component hybrid sensor and regulator | PS | [66] |
| sll1231 | mannosyltransferase | PM | [67] |
| sll1233 | hypothetical protein | PM | [67] |
| sll1234 | S-Adenosylhomocysteine hydrolase (ahcY) | S | [60], [61], [62], [63], [64], [66], [67] |
| sll1239 | unknown protein | PS | [63], [67] |
| sll1242 | hypothetical protein | PM | [66], [67] |
| sll1244 | 50S ribosomal protein L9 | PS | [27], [28], [63], [65], [67] |
| sll1249 | pantothenate synthetase/cytidylate kinase | PS | [67] |
| sll1252 | hypothetical protein | PS | [67] |
| sll1258 | dCTP deaminase | PS | [64], [66], [67] |
| sll1260 | 30S ribosomal protein S2 | M | [C], [59], [63], [64], [65], [66], [67] |
| sll1261 | elongation factor TS (tsf) | M, S | [C], [27], [28], [60], [61], [62], [63], [64], [65], [66], [67] |
| sll1262 | hypothetical protein | PS | [67] |
| sll1265 | unknown protein | PM | [67] |
| sll1270 | periplasmic substrate-binding and integral membrane protein of the ABC-type Bgt permease for basic amino acids and glutamine BgtB | PM | [66], [67] |
| sll1271 | probable porin; major outer membrane protein | PM | [67] |
| sll1272 | unknown protein | PS | [67] |
| sll1273 | unknown protein | PS | [66], [67] |
| sll1274 | hypothetical protein | PS | [67] |
| sll1275 | pyruvate kinase 2 (pyk2) | S | [62], [63], [65], [66], [67] |
| sll1276 | ATP-binding protein of ABC transporter | PM | [67] |
| sll1280 | hypothetical protein | PM | [67] |
| sll1282 | riboflavin synthase beta subunit | PM | [63], [64], [67] |
| sll1283 | similar to stage II sporulation protein D | PM | [66], [67] |
| sll1284 | serine esterase | M | [6], [27], [28], [63], [67] |
| sll1285 | hypothetical protein | PM | [66] |
| sll1286 | transcriptional regulator | PM | [67] |
| sll1289 | hypothetical protein | PS | [67] |
| sll1290 | probable ribonuclease II | PM | [66], [67] |
| sll1294 | methyl-accepting chemotaxis protein | P | [4], [67] |
| sll1296 | two-component hybrid sensor and regulator | PM | [27], [28], [66], [67] |
| sll1298 | putative carboxymethylenebutenolidase | PM | [63], [67] |
| sll1299 | acetate kinase | PS | [67] |
| sll1300 | putative methyltransferase | PS | [67] |
| sll1304 | unknown protein | PS | [64], [67] |
| sll1305 | probable hydrolase | PS | [63], [65], [66], [67] |
| sll1306 | hypothetical protein | M, PP | [C], [6], [55], [64], [67] |
| sll1307 | hypothetical protein | M, O, PP | [C], [3], [27], [28], [55], [56], [66], [67] |
| sll1308 | probable oxidoreductase | PS | [63], [67] |
| sll1314 | periplasmic protein, putative C4-dicarboxylase binding protein, | PP | [55], [64], [66], [67] |
| sll1315 | unknown protein | PM | [67] |
| sll1316 | cytochrome b6f-complex iron–sulfur subunit, PetC1 | M, T | [C], [5], [6], [27], [28], [67] |
| sll1317 | cytochrome f | M | [58], [67] |
| sll1322 | ATP synthase A chain of CF(0) | PM | [67] |
| sll1323 | ATP synthase subunit b’, AtpG | M, P, T | [C], [5], [8], [10], [58], [67] |
| sll1324 | ATP synthase subunit b, AtpF | M, P, T | [C], [5], [8], [10], [58], [67] |
| sll1325 | ATP synthase subunit | M | [C], [58], [67] |
| sll1326 | ATP synthase alpha subunit, AtpA | M, P, S, T | [5], [6], [8], [10], [58], [59], [60], [61], [62], [63], [64], [65], [66], [67] |
| sll1327 | ATP synthase subunit | M | [58], [66], [67] |
| sll1329 | ATP synthase, β-subunit | M | [59], [67] |
| sll1330 | two-component system response regulator OmpR subfamily | PS | [67] |
| sll1334 | two-component sensor histidine kinase | PS | [66] |
| sll1336 | hypothetical protein | PM | [63], [64], [67] |
| sll1338 | hypothetical protein | M, O | [C], [3], [67] |
| sll1341 | bacterioferritin | PS | [63], [65], [66], [67] |
| sll1342 | glyceraldehyde-3-phosphate dehydrogenase (GAPDH2) | M, S, T | [5], [27], [28], [56], [59], [60], [62], [63], [64], [65], [66], [67] |
| sll1343 | aminopeptidase | PS | [64], [67] |
| sll1349 | phosphoglycolate phosphatase | PM | [67] |
| sll1350 | hypothetical protein | PM | [67] |
| sll1354 | single-strand-DNA-specific exonuclease RecJ | PM | [67] |
| sll1356 | glycogen phosphorylase | PS | [63], [65], [66], [67] |
| sll1358 | oxalate decarboxylate | M, O, PP | [C], [3], [55], [56], [64], [67] |
| sll1360 | DNA polymerase III subunit gamma/tau | PM | [66] |
| sll1362 | isoleucyl-tRNA synthetase | PM | [63], [66], [67] |
| sll1363 | ketol-acid-reductoisomerase (ilvC) | M, P, S, T | [C], [5], [8], [60], [61], [62], [63], [64], [65], [66], [67] |
| sll1365 | unknown protein | PM | [67] |
| sll1366 | putative SNF2 helicase | PS | [67] |
| sll1367 | hypothetical protein | PS | [67] |
| sll1370 | mannose-1-phosphate guanylyltransferase | PM | [67] |
| sll1371 | putative ABC transporter substrate-binding protein | PS | [63], [67] |
| sll1374 | probable sugar transporter | PM | [66] |
| sll1376 | hypothetical protein | PM | [67] |
| sll1377 | probable glycosyltransferase | PM | [66], [67] |
| sll1378 | hypothetical protein | PP | [55], [67] |
| sll1380 | hypothetical protein | PP | [55], [63], [66], [67] |
| sll1383 | probable myo-inositol-1(or 4)-monophosphatase | PS | [64], [67] |
| sll1384 | similar to DnaJ protein | PS | [67] |
| sll1386 | hypothetical protein | PS | [66] |
| sll1390 | hypothetical protein | PM | [67] |
| sll1393 | glycogen (starch) synthase | PS | [63], [64], [65], [66], [67] |
| sll1394 | peptide methionine sulfoxide reductase | PS | [67] |
| sll1395 | dTDP-6-deoxy-L-mannose-dehydrogenase | PS | [67] |
| sll1396 | unknown protein | PS | [67] |
| sll1398 | photosystem II reaction center 13 kDa protein (psb28) | M, S, T | [C], [5], [6], [27], [28], [62], [63], [65], [66] |
| sll1399 | hypothetical protein | PS | [67] |
| sll1401 | unknown protein | PS | [66] |
| sll1404 | biopolymer transport protein, ExbB/TolQ | P | [4] |
| sll1405 | exbD protein, TolR | P | [8], [66] |
| sll1406 | ferrichrome-iron receptor FhuA | O | [3], [67] |
| sll1411 | hypothetical protein | PS | [66] |
| sll1414 | hypothetical protein | PM | [63], [65], [67] |
| sll1415 | hypothetical protein | PS | [67] |
| sll1418 | photosystem II oxygen-evolving complex 23K protein PsbP homolo | M | [C], [67] |
| sll1423 | global nitrogen regulator | M | [6], [66], [67] |
| sll1424 | hypothetical protein | S | [61], [66] |
| sll1425 | proline-tRNA ligase | PM | [63], [64], [66], [67] |
| sll1427 | protease | PM | [67] |
| sll1430 | adenine phosphoribosyltransferase | PS | [67] |
| sll1433 | hypothetical protein | PM | [66], [67] |
| sll1434 | penicillin-binding protein | PM | [66], [67] |
| sll1435 | glutamyl-tRNA(Gln) amidotransferase subunit B | PS | [66], [67] |
| sll1439 | unknown protein | PS | [66] |
| sll1440 | pyridoxamine 5'-phosphate oxidase | PS | [63], [64], [67] |
| sll1441 | acyl-lipid desaturase (omega-3) | PM | [67] |
| sll1443 | CTP synthetase | PS | [67] |
| sll1444 | 3-isopropylmalate dehydratase small subunit | PS | [27], [28], [67] |
| sll1450 | ABC transporter; Nitrate/nitrite binding protein, NrtA | M, P | [C], [4], [10], [63], [64], [66], [67] |
| sll1451 | nitrate/nitrite transport system permease protein | PM | [67] |
| sll1452 | nitrate/nitrite transport system ATP-binding protein | PM | [64], [67] |
| sll1453 | nitrate/nitrite transporter, ATP-binding subunit (NrtD) | M | [59], [66], [67] |
| sll1456 | unknown protein | PS | [67] |
| sll1457 | probable glycosyltransferase | PM | [67] |
| sll1459 | stationary-phase survival protein SurE homolog | PS | [67] |
| sll1463 | cell division protein, FtsH | T | [5], [63], [65], [66], [67] |
| sll1464 | hypothetical protein | PS | [64] |
| sll1469 | hypothetical protein | PS | [67] |
| sll1470 | 3-isopropylmalate dehydratase large subunit | PM | [66], [67] |
| sll1471 | phycobilisome rod-core linker polypeptide (LRC) | M, P, T | [4], [5], [59], [63], [66], [67] |
| sll1473 | a part of phytochrome-like sensor histidine kinase gene (disrupted by insertion of IS) | PM | [67] |
| sll1475 | a part of phytochrome-like sensor histidine kinase gene (disrupted by insertion of IS) | PS | [67] |
| sll1477 | hypothetical protein | PM | [66], [67] |
| sll1479 | 6-phosphogluconolactonase | PS | [27], [28], [63], [64], [66], [67] |
| sll1481 | ABC-transporter membrane fusion protein | PM | [67] |
| sll1483 | cell surface lipoprotein H | PP | [27], [28], [55], [56], [67] |
| sll1484 | type 2 NADH dehydrogenase, NdbC | M, P | [4], [59], [66], [67] |
| sll1489 | circadian phase modifier CpmA homolog | PM | [66], [67] |
| sll1491 | periplasmic WD-repeat protein, beta transducin-like protein | PP | [55], [56], [64], [67] |
| sll1495 | hypothetical protein | PM | [67] |
| sll1496 | mannose-1-phosphate guanyltransferase | PM | [67] |
| sll1498 | carbamoyl-phosphate synthase small chain | PM | [67] |
| sll1499 | ferredoxin-dependent glutamate synthase | PM | [63], [65], [66], [67] |
| sll1502 | NADH-dependent glutamate synthase large subunit | PM | [63], [65], [66], [67] |
| sll1505 | hypothetical protein | PS | [66] |
| sll1507 | hypothetical protein | PP | [55] |
| sll1508 | UDP-3-0-acyl N-acetylglcosamine deacetylase | PS | [67] |
| sll1510 | unknown protein | PM | [66] |
| sll1513 | c-type cytochrome synthesis protein | PM | [67] |
| sll1514 | 16.6 kDa small heat shock molecular chaperone (hspA, hsp17) | S | [62], [66] |
| sll1516 | hypothetical protein | PS | [67] |
| sll1520 | DNA repair protein RecN | PS | [67] |
| sll1521 | flavoprotein | PM | [63], [64], [66], [67] |
| sll1524 | hypothetical protein | PS | [67] |
| sll1525 | phosphoribulokinase | C, M, S, T | [5],[56], [27], [28], [56], [59], [60], [61], [63], [64], [65], [66], [67] |
| sll1526 | hypothetical protein | PM | [63], [65], [66], [67] |
| sll1527 | unknown protein | PS | [66], [67] |
| sll1528 | unknown protein | PM | [66], [67] |
| sll1530 | unknown protein | PS | [63], [66], [67] |
| sll1531 | unknown protein | PM | [67] |
| sll1532 | hypothetical protein | PP | [55], [56], [67] |
| sll1533 | twitching mobility protein | M | [C], [66], [67] |
| sll1534 | probable glycosyltransferase | PS | [66] |
| sll1535 | putative sugar transferase | PM | [67] |
| sll1536 | molybdopterin biosynthesis moeB protein | S | [60], [61], [63], [64], [65], [66], [67] |
| sll1537 | similar to mutator MutT protein | PS | [63], [66], [67] |
| sll1538 | similar to beta-hexosaminidase a precursor | PS | [66], [67] |
| sll1541 | hypothetical protein | PS | [67] |
| sll1542 | hypothetical protein | PS | [67] |
| sll1544 | two-component response regulator NarL subfamily | PS | [66] |
| sll1545 | GST | M | [27], [28], [59], [64], [66], [67] |
| sll1546 | exopolyphosphatase | PS | [67] |
| sll1549 | phosphoglycerate mutaseH | PP | [55], [64], [67] |
| sll1550 | probable porin; major outer membrane protein | PM | [67] |
| sll1553 | phenylalanyl-tRNA synthase | S | [61], [63], [64], [66], [67] |
| sll1555 | two-component hybrid sensor and regulator | PS | [67] |
| sll1556 | isopentenyl-dephosphate delta-isomerase | PM | [66], [67] |
| sll1557 | succinyl-CoA synthetase alpha chain | PM | [67] |
| sll1558 | mannose-1-phosphate guanyltransferase | PM | [67] |
| sll1559 | soluble hydrogenase 42kD subunit | S | [60], [63], [65], [66], [67] |
| sll1561 | proline oxidase | PM | [63], [66], [67] |
| sll1563 | unknown protein | PS | [66], [67] |
| sll1564 | putative lyase | PS | [67] |
| sll1566 | glucosylglycerolphosphate synthase | PS | [64], [66], [67] |
| sll1568 | fibrillin | PM | [67] |
| sll1570 | unknown protein | PM | [67] |
| sll1571 | hypothetical protein | PM | [67] |
| sll1572 | DNA polymerase III alpha subunit | PS | [66], [67] |
| sll1577 | phycocyanin β subunit, CpcB | M, P, S, T | [4], [5], [6], [10], [27], [28], [56], [58], [59], [60], [61], [63], [64], [65], [66], [67] |
| sll1578 | phycocyanin α subunit, CpcA | M, P, S, T | [C], [5], [6], [8], [10], [27], [28], [58], [61], [63], [64], [65], [66], [67] |
| sll1579 | phycocyanin, CpcC | P | [4], [63], [65], [66], [67] |
| sll1580 | phycocyanin ass. linker protein, CpcC2 | M, P | [C], [4], [27], [28], [63], [64], [65], [66], [67] |
| sll1581 | GumB protein | M, O | [C], [3], [67] |
| sll1582 | hypothetical protein | S | [61], [66], [67] |
| sll1583 | DNA ligase (lig, ligA) | M, S, T | [C], [5], [62], [63], [65], [67] |
| sll1586 | unknown protein | PM | [66] |
| sll1590 | two-component sensor histidine kinase | PM | [63], [65] |
| sll1594 | ndhF3 operon transcriptional regulator, LysR family protein | PS | [67] |
| sll1595 | circadian clock protein KaiC homolog | PS | [66], [67] |
| sll1605 | (3R)-hydroxymyristol acyl carrier protein dehydrase | PM | [67] |
| sll1606 | hypothetical protein | PM | [66] |
| sll1608 | hypothetical protein | PM | [67] |
| sll1609 | hypothetical protein | PS | [66] |
| sll1612 | folylpolyglutamate synthase | PS | [66], [67] |
| sll1614 | cation-transporting P-type ATPase | PM | [66], [67] |
| sll1615 | thiophen and furan oxidation protein | PS | [67] |
| sll1620 | hypothetical protein | PM | [27], [28], [66] |
| sll1621 | type 2 peroxiredoxin | S | [27], [28], [60], [61], [63], [64], [65], [66], [67] |
| sll1623 | ABC transporter ATP-binding protein | PS | [67] |
| sll1624 | two-component response regulator | PS | [66], [67] |
| sll1625 | succinate dehydrogenase iron- sulphur protein subunit | PS | [67] |
| sll1626 | SOS function regulatory protein LexA repressor (lexA) | M, S, T | [C], [5], [6], [60], [62], [63], [64], [65], [66], [67] |
| sll1628 | hypothetical protein | P | [10], [67] |
| sll1630 | hypothetical protein | M | [C] |
| sll1633 | cell division protein FtsZ | PM | [63], [64], [66], [67] |
| sll1634 | hypothetical protein | PM | [67] |
| sll1635 | Thy1 protein homolog | PS | [67] |
| sll1636 | ferripyochelin binding protein | PS | [67] |
| sll1638 | hypothetical protein | M, O, P, T | [C], [3], [5], [8], [10], [67] |
| sll1640 | hypothetical protein | PS | [67] |
| sll1641 | glutamate decarboxylase | PS | [64], [66], [67] |
| sll1653 | 2-phytyl-1,4-benzoquinone methyltransferase | PS | [67] |
| sll1654 | hypothetical protein | PS | [63], [65], [66], [67] |
| sll1655 | similar to biotin [acetyl-CoA-carboxylase] ligase | PM | [66], [67] |
| sll1656 | hypothetical protein | PM | [67] |
| sll1662 | probable prephenate dehydratase | PS | [64], [67] |
| sll1663 | phycocyanin alpha phycocyanobilin lyase related protein | PS | [63], [64], [65], [66], [67] |
| sll1664 | probable glycosyl transferase | PS | [67] |
| sll1665 | hypothetical protein (Synechocystis only) | P | [4], [67] |
| sll1667 | mitochondrial outer membrane 72-kDa protein | PP | [55], [67] |
| sll1669 | shikimate kinase | PM | [67] |
| sll1672 | two-component hybrid sensor and regulator | PM | [66], [67] |
| sll1673 | two-component response regulator | PS | [67] |
| sll1675 | hypothetical protein | PS | [27], [28], [64] |
| sll1676 | 4-alpha-glucanotransferase | PS | [64], [66], [67] |
| sll1677 | similar to spore maturation protein B | PM | [67] |
| sll1679 | HhoA protease | M, P, PP, S | [C], [10], [55], [62], [66], [67] |
| sll1682 | alanine dehydrogenase | PS | [63], [64], [66], [67] |
| sll1687 | unknown protein | PM | [66] |
| sll1688 | threonine synthase | PS | [67] |
| sll1689 | group2 RNA polymerase sigma factor SigE | PS | [67] |
| sll1693 | hypothetical protein | S | [62], [64], [67] |
| sll1694 | Pilin, PilA1 | P | [4], [27], [28], [63], [65], [66], [67] |
| sll1696 | hypothetical protein | P | [10], [67] |
| sll1697 | hypothetical protein | T | [5] |
| sll1699 | ABC transporter; Oligopeptide binding protein | P | [8], [10], [66], [67] |
| sll1703 | protease IV | PM | [63] |
| sll1709 | 3-ketoacyl-acyl carrier protein reductase | PS | [64], [67] |
| sll1712 | DNA binding protein HU | PS | [27], [28], [63], [65], [67] |
| sll1713 | histidinol-phosphate aminotransferase | PM | [66], [67] |
| sll1721 | pyruvate dehydrogenase component E1, a-subunit | M | [59], [63], [64], [65], [66], [67] |
| sll1723 | probable glycosyltransferase | PS | [66] |
| sll1725 | ATP-binding protein of ABC transporter | PM | [66] |
| sll1732 | NADH dehydrogenase subunit, NdhF3 | M | [58] |
| sll1733 | NADH dehydrogenase subunit 4, NdhD3 | M | [58], [67] |
| sll1734 | protein involved in high affinity CO2 uptake, CupA | M, T | [5], [58], [67] |
| sll1735 | protein homologous to secreted protein MPB70 | M | [58], [67] |
| sll1739 | unknown protein | PS | [67] |
| sll1740 | 50S ribosomal protein L19 | PS | [63], [66] |
| sll1742 | transcription antitermination protein NusG | M | [59], [67] |
| sll1743 | 50S ribosomal protein L11 | PS | [63], [65], [66], [67] |
| sll1744 | 50S ribosomal protein L1 | PS | [63], [65], [66], [67] |
| sll1745 | ribosomal protein L10 | M | [C], [63], [67] |
| sll1746 | 50S ribosomal protein l12 | M, S | [C], [6], [27], [28], [60], [63], [64], [65], [66], [67] |
| sll1747 | chorismate synthase (aroC) | S | [62], [63], [64], [66], [67] |
| sll1750 | urease alpha subunit | PM | [63], [64], [65], [66], [67] |
| sll1752 | hypothetical protein | PS | [66] |
| sll1757 | hypothetical protein | M, P | [4], [58], [66], [67] |
| sll1760 | homoserine kinase | PM | [67] |
| sll1762 | amino-acid ABC transporter binding proteinH | M, PP | [C], [27], [28], [55], [63], [64], [65], [66], [67] |
| sll1766 | hypothetical protein | PM | [67] |
| sll1767 | 30S ribosomal protein S6 | M | [C], [67] |
| sll1768 | probable oligopeptides ABC transporter permease protein | PM | [67] |
| sll1769 | hypothetical protein | T | [5], [27], [28] |
| sll1770 | hypothetical protein | PM | [67] |
| sll1771 | protein serin-threonin phosphatase | PS | [67] |
| sll1772 | DNA mismatch repair protein MutS | PM | [66], [67] |
| sll1774 | hypothetical protein | PM | [66], [67] |
| sll1775 | hypothetical protein | PM | [66], [67] |
| sll1776 | deoxyribose-phosphate aldolase | PS | [64], [66], [67] |
| sll1784 | hypothetical protein | PP | [27], [28], [55], [56], [63], [64], [65], [67] |
| sll1785 | hypothetical protein | PP | [27], [28], [55], [63], [65], [66], [67] |
| sll1786 | putative deoxyribonuclease, tatD homolog | PS | [66], [67] |
| sll1787 | RNA polymerase beta subunit | PM | [63], [64], [65], [66], [67] |
| sll1789 | RNA polymerase beta prime submit | S | [61], [63], [65], [66], [67] |
| sll1799 | 50S ribosomal protein L3 | M | [59], [66], [67] |
| sll1800 | 50S ribosomal protein L4 | PS | [66], [67] |
| sll1801 | 50S ribosomal protein L23 | PS | [66], [67] |
| sll1802 | 50S ribosomal protein L2 | PS | [63], [65], [66], [67] |
| sll1803 | 50S ribosomal protein L22 | PS | [67] |
| sll1804 | 30S ribosomal protein S3 | M | [59], [63], [65], [66], [67] |
| sll1805 | 50S ribosomal protein L16 | PS | [67] |
| sll1806 | 50S ribosomal protein L14 | PS | [27], [28], [66], [67] |
| sll1807 | 50S ribosomal protein L24 | PS | [63], [65], [67] |
| sll1808 | 50S ribosomal protein L5 | M | [C], [66], [67] |
| sll1809 | 30S ribosomal protein S8 | PM | [66], [67] |
| sll1810 | 50S ribosomal protein L6 (rplF, rpl6) | S | [62], [63], [66], [67] |
| sll1811 | 50S ribosomal protein L18 | PS | [66], [67] |
| sll1812 | 30S ribosomal protein S5 | PS | [63], [65], [66], [67] |
| sll1813 | 50S ribosomal protein L15 | PS | [63], [67] |
| sll1814 | preprotein translocase SecY subunit | PM | [67] |
| sll1815 | adenylate kinase (adk) | M, S | [C], [6], [27], [28], [61], [62], [63], [64], [65], [66], [67] |
| sll1816 | 30S ribosomal protein S13 | PS | [63], [65], [66], [67] |
| sll1817 | 30S ribosomal protein S11 | PS | [66], [67] |
| sll1818 | RNA polymerase, a-subunit | M, S | [59], [61], [62], [63], [64], [65], [66], [67] |
| sll1819 | 50S ribosomal protein L17 | PS | [67] |
| sll1820 | tRNA pseudouridine synthase 1 | PS | [66], [67] |
| sll1821 | 50S ribosomal protein L13 | PS | [63], [66], [67] |
| sll1822 | 30S ribosomal protein S9 | PS | [63], [66], [67] |
| sll1823 | adenylosuccinate synthetase | PM | [64], [67] |
| sll1825 | aklaviketone reductase | S | [27], [28], [60], [64], [66], [67] |
| sll1830 | unknown protein | PS | [63], [64], [65], [66], [67] |
| sll1833 | penicillin-binding protein | PM | [66], [67] |
| sll1835 | hypothetical protein | M, O, P, PP, S | [C], [3], [6], [8], [10], [55], [56], [61], [67] |
| sll1837 | hypothetical protein | PP | [55] |
| sll1841 | pyruvate dehydrogenase component E2 | M, S, T | [56], [59], [62], [63], [64], [65], [66], [67] |
| sll1848 | putative acyltransferas | PS | [67] |
| sll1852 | nucleoside diphosphate kinase | PM | [27], [28], [63], [64], [65], [66], [67] |
| sll1854 | exodeoxyribonuclease III | PS | [67] |
| sll1858 | unknown protein | PM | [63], [66] |
| sll1862 | hypothetical protein | M | [C] |
| sll1863 | unknown protein | PS | [64] |
| sll1864 | probable chloride channel protein | PM | [66] |
| sll1868 | DNA primase | PS | [66] |
| sll1870 | ATP-binding protein of ABC transporter | PS | [67] |
| sll1871 | sensory transduction histidine kinase | S | [61], [67] |
| sll1872 | transcriptional regulator | PM | [67] |
| sll1873 | unknown protein | PS | [27], [28], [63], [65], [66], [67] |
| sll1876 | coproporphyrinogen III oxidase, anaerobic (oxygen-independent) | PS | [66] |
| sll1878 | iron(III)-transport ATP-binding protein | PS | [67] |
| sll1879 | two-component response regulator | PS | [67] |
| sll1883 | arginine biosynthesis bifunctional protein ArgJ | PM | [27], [28], [63], [66], [67] |
| sll1884 | hypothetical protein | PS | [67] |
| sll1885 | unknown protein | PM | [67] |
| sll1888 | two-component sensor histidine kinase | PM | [67] |
| sll1891 | hypothetical protein | PP | [56], [64], [67] |
| sll1893 | cyclase | PS | [67] |
| sll1894 | riboflavin biosynthesis protein RibA | PM | [67] |
| sll1895 | hypothetical protein | PM | [66], [67] |
| sll1898 | hypothetical protein | PM | [67] |
| sll1902 | hypothetical protein | PS | [66] |
| sll1905 | two-component hybrid sensor and regulator | PM | [66], [67] |
| sll1906 | hypothetical protein | PM | [66], [67] |
| sll1908 | D-3-phosphoglycerate dehydrogenase (serA) | S | [27], [28], [60], [62], [63], [64], [65], [66], [67] |
| sll1909 | probable methyltransferase | PM | [67] |
| sll1910 | protein conferring resistance to acetazolamide Zam | PS | [66], [67] |
| sll1911 | hypothetical protein | PS | [67] |
| sll1913 | hypothetical protein | S | [62], [67] |
| sll1915 | carbonic anhydrase | M | [C], [67] |
| sll1920 | copper-transporting P-type ATPase PacS | PM | [67] |
| sll1925 | hypothetical protein | PM | [67] |
| sll1927 | ABC transporter ATP-binding protein | PS | [66], [67] |
| sll1929 | competence protein ComE | PM | [66] |
| sll1931 | serine hydroxymethyltransferase | PS | [63], [65], [67] |
| sll1932 | DnaK protein | PS | [66], [67] |
| sll1934 | hypothetical protein | PS | [66] |
| sll1940 | hypothetical protein | O | [3] |
| sll1941 | DNA gyrase subunit A | M | [C], [67] |
| sll1942 | unknown protein | PM | [67] |
| sll1945 | 1-deoxyxylulose-5-phosphate synthase | PM | [63], [64], [65], [66], [67] |
| sll1946 | hypothetical protein | PM | [67] |
| sll1949 | unknown protein | PM | [67] |
| sll1950 | unknown protein | PS | [67] |
| sll1951 | unknown protein | PM | [66], [67] |
| sll1958 | histidinol phosphate aminotransferase | PS | [63], [67] |
| sll1961 | hypothetical protein | S | [62], [66], [67] |
| sll1967 | probable RNA methyltransferase | PS | [67] |
| sll1979 | hypothetical protein | PS | [67] |
| sll1980 | thioredoxin, TrxA | T | [5], [67] |
| sll1981 | acetolactate synthase | PM | [63], [64], [65], [66], [67] |
| sll1987 | Catalase-peroxidase (KatG) | T | [56], [63], [64], [65], [66], [67] |
| sll1988 | 33 kDa chaperonin | PS | [66], [67] |
| sll1994 | porphobilinogen synthase (5-aminolevulinate dehydratase) | PS | [63], [64], [65], [67] |
| sll2001 | leucine aminopeptidase | PS | [63], [64], [65], [66], [67] |
| sll2002 | hypothetical protein | PS | [63], [67] |
| sll2003 | hypothetical protein | PM | [66], [67] |
| sll2005 | DNA gyrase B subunit, gyrB | PS | [63], [64], [66], [67] |
| sll2008 | processing protease | PS | [67] |
| sll2009 | processing protease | PS | [67] |
| sll2010 | UDP-N-acetylmuramoylalanine--D-glutamate ligase | PS | [66], [67] |
| sll2011 | hypothetical protein | PS | [67] |
| sll2014 | sugar fermentation stimulation protein | PS | [67] |
| sll5080 | non-heme chloroperoxidase | PS | [66] |
| sll7087 | unknown protein | PS | [66] |
| sll8004 | hypothetical protein | PS | [66] |
| slr0001 | hypothetical protein | S | [27], [28], [62], [63], [64], [65], [66], [67] |
| slr0006 | unknown protein | PM | [27], [28], [66], [67] |
| slr0007 | probable sugar-phosphate nucleotidyltransferase | PS | [67] |
| slr0009 | ribulose-bisphosphate carboxylase large chain (RbcL) | C, M, P, S | [4],[56], [59], [60], [61], [62], [63], [64], [65], [66], [67] |
| slr0012 | ribulose bisphosphate caboxylase small subunit (rbcS) | C, M, S, T | [C],[5], [6], [27], [28], [56], [60], [62], [63], [64], [65], [66], [67] |
| slr0013 | hypothetical protein | M, P | [C], [4], [8], [10], [66], [67] |
| slr0015 | lipid A disaccharide synthase | PM | [63], [67] |
| slr0016 | hypothetical protein | PM | [67] |
| slr0017 | UDP-N-acetylglucosamine 1-carboxyvinyltransferase | PM | [66], [67] |
| slr0018 | fumarase | PM | [66], [67] |
| slr0021 | protease | PM | [66], [67] |
| slr0023 | unknown protein | PS | [67] |
| slr0031 | hypothetical protein | PM | [64], [66], [67] |
| slr0032 | probable branched-chain amino acid aminotransferase | PS | [63], [64], [65], [66], [67] |
| slr0038 | hypothetical protein | M | [C], [63], [65], [66], [67] |
| slr0039 | hypothetical protein | PS | [66], [67] |
| slr0040 | aicarbonate transporter, CmpA | O, P, PP, T | [3], [4], [5], [8], [56] |
| slr0042 | probable porin; major outer membrane protein | PM | [63] |
| slr0049 | hypothetical protein | PM | [63], [64], [65], [67] |
| slr0050 | hypothetical protein YCF56 | PM | [67] |
| slr0051 | carbonic anhydrase | PP | [55], [56], [63], [67] |
| slr0055 | anthranilate synthase component II | PS | [63], [67] |
| slr0058 | hypothetical protein | PS | [63], [65] |
| slr0063 | pilus biogenesis protein homologous to general secretion pathway protein E | PS | [63], [65], [66], [67] |
| slr0064 | hypothetical protein | PS | [67] |
| slr0065 | hypothetical protein | PS | [27], [28], [67] |
| slr0067 | MRP protein homolog | PM | [67] |
| slr0070 | methionyl-tRNA formyltransferase | PS | [67] |
| slr0072 | glucose inhibited division protein B | PM | [67] |
| slr0073 | two-component sensor histidine kinase | PS | [64], [67] |
| slr0074 | ABC transporter subunit | PS | [64], [66], [67] |
| slr0075 | ABC transporter subunit | M | [6], [67] |
| slr0076 | hypothetical protein | PS | [67] |
| slr0077 | cysteine desulfurase | PS | [64], [67] |
| slr0079 | probable general secretion pathway protein E | PS | [66], [67] |
| slr0080 | ribonuclease H | PS | [67] |
| slr0082 | hypothetical protein | PS | [67] |
| slr0083 | RNA helicase Light | PS | [66], [67] |
| slr0086 | similar to DnaK protein | PS | [67] |
| slr0088 | beta-carotene ketolase | PS | [67] |
| slr0089 | gamma-tocopherol methyltransferase | PM | [67] |
| slr0096 | low affinity sulfate transporter | PM | [66] |
| slr0104 | hypothetical protein | S | [61] |
| slr0106 | unknown protein | PS | [66], [67] |
| slr0110 | hypothetical protein | PS | [63], [67] |
| slr0112 | unknown protein | PM | [67] |
| slr0114 | putative PP2C-type protein phosphatase | PM | [67] |
| slr0115 | response regulator for energy transfer from phycobilisomes to photosystems | PS | [67] |
| slr0116 | phycocyanobilin:ferredoxin oxidoreductase | PM | [67] |
| slr0118 | thiamine biosynthesis protein ThiC | PM | [64], [67] |
| slr0119 | hypothetical protein | PM | [66] |
| slr0120 | probable tRNA/rRNA methyltransferase | PS | [67] |
| slr0121 | hypothetical protein | PM | [66], [67] |
| slr0143 | WD-repeat protein, Hat protein, involved in the control of a high-affinity transport system for inorganic carbon | PM | [66], [67] |
| slr0144 | hypothetical protein | PS | [66], [67] |
| slr0145 | unknown protein | S | [27], [28] |
| slr0146 | hypothetical protein | PS | [63], [65] |
| slr0147 | hypothetical protein | PM | [63], [65], [66], [67] |
| slr0148 | hypothetical protein | PS | [67] |
| slr0149 | hypothetical protein | PS | [63], [67] |
| slr0151 | hypothetical protein | M, P | [C], [4], [6], [8], [10], [27], [28], [58], [67] |
| slr0152 | serine/threonine protein kinase | PM | [66] |
| slr0156 | C1pB protein | S | [61], [66], [67] |
| slr0161 | twitching motility protein PilT | M | [C], [59], [63], [65], [67] |
| slr0162 | a part of pilC, pilin biogenesis protein, required for twitching motility | PS | [67] |
| slr0163 | a part of pilC, pilin biogenesis protein, required for twitching motility | PM | [67] |
| slr0164 | ATP dependant Clp protease proteolytic subunit | S | [61], [63], [64], [65], [66], [67] |
| slr0165 | ATP-dependent Clp protease proteolytic subunit | M | [C], [64], [66], [67] |
| slr0169 | hypothetical protein | PM | [66], [67] |
| slr0171 | photosystem I assembly related protein Ycf37 | PM | [67] |
| slr0172 | IMP dehydrogenase | M | [6], [27], [28], [63], [65], [67] |
| slr0179 | hypothetical protein | PS | [66] |
| slr0184 | unknown protein | PS | [67] |
| slr0185 | orotate phosphoribosyltransferase (umpS) | S | [62], [63], [66], [67] |
| slr0186 | 2-isoproplylmalate synthase | S | [61], [64], [67] |
| slr0191 | amidase enhancer | PP | [55], [67] |
| slr0193 | RNA-binding protein | PS | [63], [65], [67] |
| slr0194 | ribose-5-phophate isomerase | P, S | [10], [61], [63], [64], [65], [67] |
| slr0197 | competence protein | PM | [66] |
| slr0199 | hypothetical protein | PS | [67] |
| slr0201 | heterodisulfide reductase subunit B | PS | [66], [67] |
| slr0207 | hypothetical protein | PS | [66], [67] |
| slr0208 | hypothetical protein | PS | [64] |
| slr0209 | unknown protein | PS | [66], [67] |
| slr0212 | 5-methyltetrahydrofolate--homocysteine methyltransferase | PM | [63], [66], [67] |
| slr0213 | GMP synthetase | PM | [64], [66], [67] |
| slr0220 | glycyl-tRNA synthase beta chain (glyS) | S | [62], [64], [66], [67] |
| slr0222 | two-component hybrid sensor and regulator | PS | [66] |
| slr0226 | unknown protein | PM | [66], [67] |
| slr0228 | cell division protein FtsH | PM | [63], [66], [67] |
| slr0229 | 3-hydroxyisobutyrate dehydrogenase | PM | [67] |
| slr0232 | hypothetical protein | PM | [66] |
| slr0236 | similar to glutathione S-transferase | PM | [66], [67] |
| slr0237 | glycogen operon protein GlgX homolog | PM | [64], [66], [67] |
| slr0238 | hypothetical protein | PP | [56] |
| slr0239 | precorrin-4 C11-methyltransferase | PM | [66], [67] |
| slr0242 | bacterioferritin comigratory protein homolog | PS | [27], [28], [67] |
| slr0244 | Usp1 | M | [C], [59], [63], [64], [65], [66], [67] |
| slr0250 | hypothetical protein | PM | [67] |
| slr0251 | ATP-binding protein of ABC transporter | PS | [66], [67] |
| slr0254 | hypothetical protein | PM | [67] |
| slr0257 | C-terminal protease | PP | [55], [56], [66], [67] |
| slr0260 | cob(I)alamin adenosyltransferase | PS | [66], [67] |
| slr0261 | NADH dehydrogenase subunit 7, NdhH | M, T | [C], [5], [58], [64], [67] |
| slr0280 | hypothetical protein | PM | [66], [67] |
| slr0288 | glutamate--ammonia ligase | PS | [63], [64], [65], [67] |
| slr0293 | glycine dehydrogenase | PM | [66], [67] |
| slr0298 | FraH protein homolog | PS | [67] |
| slr0301 | phosphoenolpyruvate synthase | PS | [63], [65], [66], [67] |
| slr0303 | hypothetical protein | PS | [67] |
| slr0311 | two-component sensor histidine kinase | PS | [63] |
| slr0314 | non-heme chloroperoxidase | PS | [66] |
| slr0315 | probable oxidoreductase | PM | [64], [67] |
| slr0317 | hypothetical protein | PS | [27], [28], [64], [67] |
| slr0321 | GTP-binding protein ERA homolog | PS | [66], [67] |
| slr0322 | two-component hybrid sensor and regulator | PS | [66], [67] |
| slr0323 | putative alpha-mannosidase | PS | [67] |
| slr0324 | probable oligopeptides ABC transporter permease protein | PM | [67] |
| slr0328 | low molecular weight phosphotyrosine protein phosphatase | PS | [66] |
| slr0329 | glucokinase | PM | [66], [67] |
| slr0331 | NADH dehydrogenase subunit 4 (involved in photosystem-1 cyclic electron flow) | PM | [67] |
| slr0335 | phycobilisome core-membrane linker polypeptide (LCM) | M, P, S | [4], [59], [61], [63], [65], [66], [67] |
| slr0338 | probable oxidoreductase | PM | [67] |
| slr0342 | cytochrome b6, PetB | M, T | [5], [58], [67] |
| slr0343 | cytochrome b6f complex subunit IV | M | [58] |
| slr0348 | hypothetical protein | PM | [66], [67] |
| slr0351 | hypothetical protein | PM | [67] |
| slr0354 | ATP-binding protein of ABC transporter | PS | [67] |
| slr0355 | hypothetical protein | PM | [66], [67] |
| slr0356 | hypothetical protein | O | [3] |
| slr0357 | histidyl-tRNA synthetase | PS | [64], [67] |
| slr0359 | hypothetical protein | S | [61], [66] |
| slr0362 | hypothetical protein | M, P | [C], [10], [66], [67] |
| slr0369 | cation/multidrug efflux system protein | P | [4], [67] |
| slr0370 | succinate-semialdehyde dehydrogenase (NADP+) | PM | [63], [64], [65], [66], [67] |
| slr0374 | ATPase of the AAA-family | M | [59], [63], [64], [65], [66], [67] |
| slr0377 | unknown protein | PM | [67] |
| slr0378 | similar to 7-beta-(4-carbaxybutanamido)cephalosporanic acid acylase | PM | [66] |
| slr0379 | thymidylate kinase | PS | [66], [67] |
| slr0380 | hypothetical protein | PM | [67] |
| slr0381 | lactoylglutathione lyase | PS | [27], [28], [64] |
| slr0384 | sulfoquinovosyldiacylglycerol biosynthesis protein SqdX | PM | [67] |
| slr0386 | unknown protein | PM | [67] |
| slr0387 | cysteine desulfurase NifS | PM | [66] |
| slr0394 | phosphoglycerate kinase | P, S, T | [4],[5], [56], [60], [61], [62], [63], [64], [65], [66], [67] |
| slr0397 | hypothetical protein | PS | [67] |
| slr0398 | unknown protein | PS | [63] |
| slr0399 | chaperon-like protein for quinone binding in photosystem II | PS | [67] |
| slr0400 | hypothetical protein | PM | [67] |
| slr0401 | periplasmic polyamine-binding protein of ABC transporter | PM | [67] |
| slr0402 | hypothetical protein | PS | [67] |
| slr0404 | hypothetical protein | PM | [67] |
| slr0406 | dihydroorotase | PS | [67] |
| slr0415 | Na+/H+ antiporter | PM | [66], [67] |
| slr0417 | DNA gyrase subunit A | PM | [63], [66], [67] |
| slr0423 | hypothetical protein | PS | [66] |
| slr0426 | GTP cyclohydrolase I | PS | [63], [64], [66], [67] |
| slr0427 | putative competence-damage protein | PS | [67] |
| slr0431 | hypothetical protein | M, O, P | [C], [3], [6], [8], [67] |
| slr0434 | elongation factor P | PS | [63], [65], [67] |
| slr0435 | biotin carboxyl carrier protein of acetyl-CoA carboxylase | PS | [66], [67] |
| slr0436 | carbon dioxide concentrating mechanism protein CcmO | PS | [67] |
| slr0439 | unknown protein | PM | [67] |
| slr0440 | hypothetical protein | PM | [67] |
| slr0442 | unknown protein | PM | [66], [67] |
| slr0444 | 3-phosphoshikimate 1-carboxyvinyltransferase | PM | [64], [67] |
| slr0447 | ABC transporter; Urea binding protein, UrtA | M, P, PP, S | [C], [4], [8], [10], [27], [28], [55], [62], [63], [64], [65], [66], [67] |
| slr0451 | putative helicase | PM | [67] |
| slr0452 | dihydroxyacid dehydratase (ilvD) | S | [61], [62], [63], [64], [65], [66], [67] |
| slr0453 | hypothetical protein | PM | [63], [64], [66], [67] |
| slr0454 | RND multidrug efflux transporter | PM | [66], [67] |
| slr0455 | hypothetical protein | M, P | [6], [10], [27], [28], [63], [65] |
| slr0457 | tRNA pseudouridine synthase B | PS | [67] |
| slr0467 | conserved component of ABC transporter for natural amino acids | PS | [67] |
| slr0469 | 30S ribosomal protein S4 | PS | [66], [67] |
| slr0476 | unknown protein | PS | [27], [28], [63], [66], [67] |
| slr0477 | phosphoribosylglycinamide formyltransferase | PS | [66], [67] |
| slr0480 | hypothetical protein YCF46 | PS | [66], [67] |
| slr0483 | hypothetical protein | P | [10], [63], [67] |
| slr0484 | two-component sensor histidine kinase | PM | [66], [67] |
| slr0500 | imidazoleglycerol-phosphate dehydratase | PM | [66], [67] |
| slr0502 | cobalamin synthesis protein cobW homolog | PS | [66], [67] |
| slr0503 | hypothetical protein YCF66 | PM | [67] |
| slr0506 | light-dependent NADPH-protochlorophyllide oxidoreductase | T | [5], [63], [65], [66], [67] |
| slr0510 | hypothetical protein | PM | [66] |
| slr0513 | ABC transporter; Iron binding protein, FutA1 | P, PP, S, T | [5], [8], [10], [55],[56], [61], [64], [66], [67] |
| slr0516 | hypothetical protein | P | [4], [67] |
| slr0519 | hypothetical protein | PS | [67] |
| slr0520 | phosphoribosyl formylglycinamidine synthase | PS | [27], [28], [64], [66], [67] |
| slr0521 | unknown protein | PM | [66] |
| slr0523 | similar to dethiobiotin synthetase | PM | [67] |
| slr0525 | Mg-protoporphyrin IX methyl transferase | PM | [67] |
| slr0528 | UDP-N-acetylmuramoylalanyl-D-glutamate--2, 6-diaminopimelate ligase | PM | [63], [66], [67] |
| slr0529 | ABC transporter; Glucosylglycerol binding protein | P | [10] |
| slr0533 | two-component sensor histidine kinase | PM | [67] |
| slr0534 | soluble lytic transglycosylase | S | [61], [67] |
| slr0535 | protease | PM | [67] |
| slr0536 | Uroporphyrinogen decarboxylase | S | [27], [28], [60], [63], [64], [66], [67] |
| slr0537 | putative sugar kinase | PS | [64], [67] |
| slr0542 | ATP-dependent protease ClpP | PM | [63], [64], [66], [67] |
| slr0543 | tryptophan synthase beta subunit | PM | [63], [65], [66], [67] |
| slr0545 | hypothetical protein | PS | [67] |
| slr0546 | indole-3-glycerol phosphate synthase | PS | [66], [67] |
| slr0549 | aspartate beta-semialdehyde dehydrogenese | PM | [64], [67] |
| slr0550 | dihydrodipicolinate synthase | PM | [67] |
| slr0551 | hypothetical protein | PS | [66], [67] |
| slr0552 | hypothetical protein | M, S | [C], [61], [62], [63], [64], [67] |
| slr0554 | hypothetical protein | PM | [66] |
| slr0556 | hypothetical protein | PS | [66] |
| slr0557 | valyl-tRNA synthetase | PM | [64], [66], [67] |
| slr0559 | periplasmic binding protein of ABC transporter for natural amino acids | PM | [63], [66], [67] |
| slr0565 | hypothetical protein | PM | [67] |
| slr0569 | unknown protein | PS | [67] |
| slr0575 | hypothetical protein | T | [5], [67] |
| slr0580 | aluminum resistance protein homolog | PS | [67] |
| slr0583 | similar to GDP-fucose synthetase | PM | [64], [67] |
| slr0585 | argininosuccinate synthetase (argG) | S | [60], [61], [62], [63], [64], [65], [66], [67] |
| slr0586 | hypothetical protein | PM | [67] |
| slr0589 | hypothetical protein | PM | [67] |
| slr0590 | hypothetical protein | PS | [67] |
| slr0591 | ribonucleoside-diphosphate reductase beta chain | PS | [67] |
| slr0592 | hypothetical protein | PS | [27], [28], [67] |
| slr0594 | hypothetical protein | PM | [67] |
| slr0597 | phosphoribosyl aminoimidazole formyltransferase | S | [61], [64], [66], [67] |
| slr0599 | serine/threonine kinase | PM | [67] |
| slr0600 | hypothetical protein-NADP-thioredoxin reductase | S | [62], [64], [66], [67] |
| slr0603 | DNA polymerase III alpha subunit | PS | [66] |
| slr0604 | GTP-binding protein | PS | [63], [66] |
| slr0605 | hypothetical protein | PS | [63], [64], [65], [66] |
| slr0606 | hypothetical protein | PM | [63], [66] |
| slr0611 | solanesyl diphosphate synthase | PS | [66], [67] |
| slr0615 | ATP-binding protein of ABC transporter | PM | [67] |
| slr0617 | unknown protein | PM | [67] |
| slr0618 | cobyric acid synthase | PM | [67] |
| slr0619 | hypothetical protein | PS | [66], [67] |
| slr0623 | thioredoxin TrxA | S, T | [5], [27], [28], [56], [60], [63], [64], [65], [66], [67] |
| slr0624 | hypothetical protein | S | [62], [67] |
| slr0626 | probable glycosyltransferase | PS | [67] |
| slr0628 | 30S ribosomal protein S14 | PS | [66] |
| slr0633 | thiamine biosynthesis protein ThiG | PM | [67] |
| slr0635 | hypothetical protein | PS | [64], [67] |
| slr0637 | hypothetical protein | PM | [67] |
| slr0638 | glycyl-tRNA synthetase alpha chain | PS | [63], [66], [67] |
| slr0643 | hypothetical protein | PM | [67] |
| slr0645 | hypothetical protein | PS | [67] |
| slr0649 | methionyl-tRNA synthetase | PS | [64], [67] |
| slr0650 | hypothetical protein | PS | [67] |
| slr0652 | phosphoribosylformimino-5-aminoimidazole carboxamide ribotide | S | [60], [63], [65], [66], [67] |
| slr0653 | principal RNA polymerase sigma factor SigA | PS | [67] |
| slr0654 | unknown protein | PM | [67] |
| slr0657 | aspartate kinase (lysC) | S | [61], [62], [64], [66], [67] |
| slr0658 | hypothetical protein | M | [59], [64], [67] |
| slr0659 | oligopeptidase A (prlC, opdA) | S | [62], [63], [64], [66], [67] |
| slr0661 | pyrroline-5-carboxylate reductase | PM | [63], [67] |
| slr0662 | arginine decarboxylase | PS | [66], [67] |
| slr0665 | aconitate hydratase | PM | [63], [65], [66], [67] |
| slr0667 | unknown protein | PS | [67] |
| slr0668 | unknown protein | PS | [67] |
| slr0670 | Usp2 | M | [C], [59], [63], [64], [65], [66], [67] |
| slr0676 | adenylylsulfate kinase | PS | [27], [28], [63], [64], [66], [67] |
| slr0677 | exbB protein, TolQ | P | [4], [8], [10], [67] |
| slr0678 | biopolymer transport ExbD like protein | PM | [67] |
| slr0680 | hypothetical protein | PS | [67] |
| slr0681 | Probable sodium/calcium exchanger protein | PP | [56] |
| slr0682 | histidinol dehydrogenase | PM | [66] |
| slr0689 | hypothetical protein | PM | [63], [67] |
| slr0692 | hypothetical protein YCF45 | PS | [66], [67] |
| slr0695 | hypothetical protein | M, O, P | [3], [8], [58], [67] |
| slr0697 | 5-oxoprolinase homolog | PM | [66], [67] |
| slr0702 | unknown protein | PM | [66] |
| slr0707 | DNA polymerase I | PM | [67] |
| slr0708 | hypothetical protein | PP | [55], [67] |
| slr0709 | hypothetical protein | PS | [66] |
| slr0710 | glutamate dehydrogenase (NADP+) | PM | [63], [67] |
| slr0711 | hypothetical protein | PS | [64] |
| slr0713 | tRNA-guanine transglycosylase | PS | [66] |
| slr0719 | unknown protein | PS | [66], [67] |
| slr0721 | malic enzyme | PS | [63], [64], [66], [67] |
| slr0723 | hypothetical protein | PS | [67] |
| slr0727 | unknown protein | PS | [67] |
| slr0729 | hypothetical protein | M, T | [C], [5], [6], [63], [65], [66] |
| slr0731 | hypothetical protein | M | [C], [64], [66], [67] |
| slr0737 | PSI subunit PsaD | M, P, T | [C], [5], [6], [8], [27], [28], [58], [59], [63], [65], [67] |
| slr0738 | anthranilate synthetase alpha-subunit | PS | [63], [67] |
| slr0739 | geranylgeranyl pyrophosphate synthase | PM | [67] |
| slr0741 | transcriptional regulator | PS | [66] |
| slr0743 | transcription termination factor NusA | M, S | [59], [62], [63], [64], [66], [67] |
| slr0744 | translation initiation factor IF-2 | PM | [63], [66], [67] |
| slr0746 | glucosylglycerolphosphate phosphatase | PS | [64], [67] |
| slr0748 | hypothetical protein | PM | [67] |
| slr0749 | light-independent protochlorophyllide reductase iron protein subunit ChlL | PM | [67] |
| slr0750 | light-independent protochlorophyllide reductase subunit ChlN | PS | [66] |
| slr0752 | enolase (eno) | C, S, T | [5], [56], [60], [61], [62], [63], [64], [65], [66], [67] |
| slr0756 | circadian clock protein KaiA homolog | PS | [63], [66], [67] |
| slr0757 | circadian clock protein KaiB homolog | PS | [63], [65] |
| slr0758 | circadian clock protein KaiC homolog | PS | [63], [66], [67] |
| slr0765 | mechanosensitive ion channel, MscS | P | [4], [67] |
| slr0769 | hypothetical protein | PM | [67] |
| slr0772 | light-independent protochlorophyllide reductase subunit ChlB | PS | [67] |
| slr0773 | hypothetical protein | PS | [67] |
| slr0774 | protein-export membrane protein SecD | PM | [67] |
| slr0775 | protein-export membrane protein SecF | PM | [67] |
| slr0776 | UDP-3-o-[3-hydroxymyristoyl] glucosamine n-acyltransferase | PM | [66], [67] |
| slr0779 | hypothetical protein | PM | [66] |
| slr0782 | putative flavin-containing monoamine oxidase | PM | [66], [67] |
| slr0783 | triosephosphate isomerase (tpiA, tpi) | S | [62], [64], [66], [67] |
| slr0786 | methionine aminopeptidase | PS | [66] |
| slr0794 | cation efflux system protein involved in nickel and cobalt tolerance | PM | [66] |
| slr0798 | zinc-transporting P-type ATPase (zinc efflux pump), ZiaA | P | [4] |
| slr0804 | probable D-alanyl-D-alanine carboxypeptidase | PM | [67] |
| slr0806 | hypothetical protein | PM | [67] |
| slr0809 | dTDP-glucose 4,6-dehydratase | PS | [63], [64], [65], [66], [67] |
| slr0818 | hypothetical protein | PM | [67] |
| slr0821 | hypothetical protein | PS | [63], [64], [65] |
| slr0822 | cation-transporting P-type ATPase PacL | PM | [66] |
| slr0823 | PSI assembly protein, Ycf3 | M, P | [C], [10], [67] |
| slr0825 | probable peptidase | PM | [64], [67] |
| slr0827 | alanine racemase | PS | [67] |
| slr0829 | unknown protein | PM | [67] |
| slr0835 | MoxR protein homolog | PS | [67] |
| slr0836 | dTDP-glucose 4,6-dehydratase | PM | [66], [67] |
| slr0838 | phosphoribosyl formylglycinamidine cyclo-ligase | PM | [63], [66], [67] |
| slr0839 | ferrochelatase | PM | [66], [67] |
| slr0841 | hypothetical protein | PP | [55], [56], [66], [67] |
| slr0842 | hypothetical protein | PM | [67] |
| slr0844 | NADH dehydrogenase subunit 5 | PM | [67] |
| slr0845 | hypothetical protein | PS | [66], [67] |
| slr0848 | hypothetical protein | M, P, S | [C], [4], [61], [63], [67] |
| slr0854 | DNA photolyase | PS | [67] |
| slr0861 | glycinamide ribonucleotide transformylase | PS | [66], [67] |
| slr0862 | probable sugar kinase | PM | [67] |
| slr0863 | hypothetical protein | S | [62], [64], [66], [67] |
| slr0864 | ATP-binding protein of ABC transporter | PS | [67] |
| slr0865 | hypothetical protein | PS | [67] |
| slr0869 | hypothetical protein | PS | [66], [67] |
| slr0872 | hypothetical protein | P | [8], [10] |
| slr0875 | large conductance mechanosensitive channel MscL | P | [8], [67] |
| slr0876 | hypothetical protein | PM | [63], [67] |
| slr0877 | glutamyl-tRNA(Gln) amidotransferase subunit A | PM | [64], [66], [67] |
| slr0879 | glycine decarboxylase complex H-protein | PS | [63] |
| slr0880 | similar to fibronectin binding protein | PS | [66] |
| slr0882 | hypothetical protein YCF84 | PM | [67] |
| slr0884 | glyceraldehyde 3-phosphate dehydrogenase1 | S | [60], [63], [64], [66], [67] |
| slr0886 | 3-oxoacyl-[acyl-carrier protein] reductase | PS | [63], [64], [66], [67] |
| slr0891 | N-acetylmuramoyl-L-alanini-amidase | M | [C], [64], [67] |
| slr0897 | probable endoglucanase | PM | [67] |
| slr0898 | ferredoxin--nitrite reductase | PS | [63], [64], [65], [66], [67] |
| slr0899 | cyanate lyase | PS | [67] |
| slr0900 | molybdopterin biosynthesis MoeA protein | PS | [66] |
| slr0901 | molybdopterin biosynthesis protein A | PS | [66] |
| slr0902 | molybdenum cofactor biosynthesis protein C, fused to molybdopterin-guanine dinucleotide biosynthesis protein MobA | PS | [67] |
| slr0906 | PSII CP47 protein | M | [58], [63], [67] |
| slr0907 | unknown protein | PM | [66], [67] |
| slr0909 | unknown protein | PS | [64], [67] |
| slr0912 | unknown protein | PS | [64], [67] |
| slr0914 | unknown protein | PM | [67] |
| slr0922 | peptidyl-tRNA hydrolase | PS | [66], [67] |
| slr0923 | hypothetical protein | S | [61], [63], [64], [65], [66], [67] |
| slr0924 | hypothetical protein | PP | [55], [64], [66], [67] |
| slr0925 | single-stranded DNA-binding protein | PS | [27], [28], [63], [65] |
| slr0927 | PSII reaction center D2 protein, PsbD2 | PM | [67] |
| slr0929 | hypothetical protein | M | [C], [6], [67] |
| slr0930 | hypothetical protein | PS | [67] |
| slr0937 | unknown protein | PS | [66], [67] |
| slr0938 | probable UDP-N-acetylmuramyl tripeptide synthetase | PS | [67] |
| slr0940 | zeta-carotene desaturase | PM | [63], [67] |
| slr0941 | hypothetical protein | PS | [66] |
| slr0942 | alcohol dehydrogenase [NADP+] | PS | [67] |
| slr0943 | fructose-bisphosphate aldolase, class I | PS | [27], [28], [63], [64], [66], [67] |
| slr0947 | response regulator for energy transfer from phycobilisomes to photosystems | S, T | [5], [61], [62], [63], [64], [65], [67] |
| slr0948 | hypothetical protein | PM | [67] |
| slr0951 | 4-diphosphocytidyl-2C-methyl-D-erythritol synthase | PS | [67] |
| slr0952 | fructose-1,6-bisphosphatase | PM | [63], [64], [66], [67] |
| slr0954 | hypothetical protein | PS | [67] |
| slr0955 | probable tRNA/rRNA methyltransferase | PS | [67] |
| slr0958 | cysteinyl-tRNA synthetase | PM | [67] |
| slr0959 | hypothetical protein | PM | [67] |
| slr0962 | unknown protein | PM | [67] |
| slr0963 | ferredoxin-sulphite reductase | M, P | [4], [59], [63], [65], [66], [67] |
| slr0965 | DNA polymerase III beta subunit | S | [61], [66], [67] |
| slr0966 | tryptophan synthase alpha chain | PS | [67] |
| slr0969 | precorrin methylase | PM | [67] |
| slr0974 | initiation factor IF-3 | PS | [67] |
| slr0975 | hypothetical protein | PM | [66] |
| slr0982 | probable polysaccharide ABC transporter ATP binding subunit | PM | [67] |
| slr0983 | glucose-1-phosphate cytidylyltransferase | PS | [66], [67] |
| slr0984 | CDP-glucose 4,6-dehydratase | PS | [63], [67] |
| slr0985 | dTDP-4-dehydrorhamnose 3,5-epimerase | PS | [67] |
| slr0989 | hypothetical protein | PS | [67] |
| slr0990 | hypothetical protein | PS | [67] |
| slr0992 | probable tRNA/rRNA methyltransferase | PS | [64] |
| slr0994 | lipoate-protein ligase B | PS | [66] |
| slr1020 | sulfolipid biosynthesis protein; (sqdB) | S | [62], [63], [64], [65], [66], [67] |
| slr1022 | N-acetylornithine aminotransferase | PM | [64], [67] |
| slr1024 | fibrillin | PS | [67] |
| slr1028 | unknown protein | PS | [66] |
| slr1030 | magnesium protoporphyrin IX chelatase subunit I | PS | [64], [67] |
| slr1031 | tyrosyl tRNA synthetase | PS | [64], [67] |
| slr1033 | unknown protein | PM | [66], [67] |
| slr1034 | hypothetical protein | M, T | [C], [5], [27], [28], [63] |
| slr1044 | methyl accepting chemotaxis protein | P, S | [4], [61], [67] |
| slr1045 | hypothetical protein YCF63 | PM | [67] |
| slr1047 | hypothetical protein | PM | [66] |
| slr1048 | hypothetical protein | PM | [67] |
| slr1051 | enoyl-[acyl-carrier-protein] reductase | PM | [63], [64], [65], [66], [67] |
| slr1053 | hypothetical protein | M | [C] |
| slr1055 | magnesium protoporphyrin IX chelatase subunit H | PM | [66], [67] |
| slr1056 | unknown protein | PM | [66], [67] |
| slr1063 | plobable glycosyltransferase | PM | [66], [67] |
| slr1064 | plobable glycosyltransferase | PS | [67] |
| slr1065 | plobable glycosyltransferase | PS | [67] |
| slr1066 | unknown protein | PS | [66], [67] |
| slr1067 | UDP-glucose 4-epimerase | PS | [67] |
| slr1072 | GDP-D-mannose dehydratase | PS | [67] |
| slr1073 | unknown protein | PS | [66] |
| slr1076 | plobable glycosyltransferase | PS | [67] |
| slr1084 | unknown protein | PM | [67] |
| slr1085 | plobable glycosyltransferase | PM | [63] |
| slr1090 | GTP-binding protein | PS | [67] |
| slr1096 | dihydrolipoamide dehydrogenase | S | [60], [63], [64], [65], [66], [67] |
| slr1097 | hypothetical protein | PS | [67] |
| slr1098 | hypothetical protein | PS | [67] |
| slr1100 | hypothetical protein | PS | [63] |
| slr1102 | hypothetical protein | PM | [66], [67] |
| slr1103 | hypothetical protein | PS | [64], [66], [67] |
| slr1104 | hypothetical protein | PS | [67] |
| slr1105 | GTP-binding protein TypA/BipA homolog | PS | [64], [67] |
| slr1106 | prohibitin | P | [8], [10], [67] |
| slr1109 | similar to ankyrin | PM | [67] |
| slr1117 | hypothetical protein | PS | [67] |
| slr1122 | hypothetical protein | PS | [67] |
| slr1123 | guanylate kinase | PS | [66], [67] |
| slr1124 | phosphoglycerate mutase | PS | [64], [67] |
| slr1128 | hypothetical protein | M, P | [4], [8], [58], [66], [67] |
| slr1129 | ribonuclease E | PS | [66], [67] |
| slr1133 | argininosuccinate lyase | T | [56], [63], [64], [66], [67] |
| slr1135 | methylase | S | [61], [66], [67] |
| slr1139 | thioredoxin | PS | [27], [28], [66], [67] |
| slr1140 | DegT/DnrJ/EryC1/StrS family protein | PM | [63], [64], [67] |
| slr1143 | hypothetical protein | PM | [67] |
| slr1145 | Monocomponent sodium-dependent glutamate permease GltS | PM | [66] |
| slr1149 | ATP-binding protein of ABC transporter | PM | [66], [67] |
| slr1159 | glycinamide ribonucleotide synthetase | PM | [67] |
| slr1160 | hypothetical protein | PP | [55], [64], [67] |
| slr1161 | hypothetical protein | PS | [63], [64], [65], [66], [67] |
| slr1164 | ribonucleotide reductase subunit alpha | PS | [66], [67] |
| slr1165 | sulfate adenylyltransferase | PS | [64], [66], [67] |
| slr1166 | UDP-glucose:tetrahydrobiopterin glucosyltransferase | PS | [67] |
| slr1167 | glycerol dehydrogenase | PM | [67] |
| slr1169 | unknown protein | PM | [67] |
| slr1171 | glutathione peroxidase-like NADPH peroxidase,glutathione peroxidase | PM | [63], [64], [66], [67] |
| slr1173 | hypothetical protein | PM | [66], [67] |
| slr1176 | glucose-1-phosphate adenylyltransferase | PS | [63], [64], [65], [66], [67] |
| slr1178 | hypothetical protein | PM | [67] |
| slr1179 | hypothetical protein | PS | [66], [67] |
| slr1181 | photosystem II D1 protein | PM | [67] |
| slr1186 | hypothetical protein | PM | [67] |
| slr1192 | zinc-containing alcohol dehydrogenase | S | [60], [66], [67] |
| slr1194 | hypothetical protein | PS | [66], [67] |
| slr1195 | hypothetical protein | PS | [63] |
| slr1196 | hypothetical protein | PP | [55], [64], [67] |
| slr1198 | 1-Cys peroxiredoxin | M, S | [27], [28], [59], [60], [61], [63], [64], [65], [66], [67] |
| slr1201 | urea transport system permease protein | PM | [67] |
| slr1202 | permease protein of sugar ABC transporter | PM | [67] |
| slr1204 | protease HtrA (M-34 as start) | O | [3] |
| slr1205 | similar to chlorobenzene dioxygenase, ferredoxin component | S | [27], [28] |
| slr1206 | hypothetical protein | PS | [67] |
| slr1207 | hypothetical protein | PM | [67] |
| slr1208 | probable oxidoreductase | PM | [66], [67] |
| slr1211 | cobalt-chelatase subunit CobN | PS | [67] |
| slr1212 | similar to two-component sensor histidine kinase | PM | [66] |
| slr1216 | Mg2+ transport protein | PM | [67] |
| slr1220 | hypothetical protein | P | [8], [10], [63], [64], [67] |
| slr1223 | hypothetical protein | PM | [64], [66], [67] |
| slr1224 | ATP-binding protein of sugar ABC transporter | PS | [67] |
| slr1225 | serine/threonine kinase | PM | [67] |
| slr1226 | phosphoribosyl aminoidazole succinocarboxamide synthetase | PS | [67] |
| slr1227 | Toc75 | M, O | [C], [3], [6], [66], [67] |
| slr1228 | peptide-chain-release factor 3 | PS | [67] |
| slr1233 | succinate dehydrogenase flavoprotein subunit | PS | [63], [66], [67] |
| slr1234 | protein kinase C inhibitor | PS | [27], [28], [63] |
| slr1235 | hypothetical protein | PS | [67] |
| slr1237 | cytosine deaminase | PS | [66] |
| slr1238 | glutathione synthetase | PS | [64], [66], [67] |
| slr1239 | pyridine nucleotide transhydrogenase alpha subunit | PM | [66], [67] |
| slr1240 | unknown protein | PS | [67] |
| slr1243 | unknown protein | PS | [66], [67] |
| slr1247 | phosphate-binding periplasmic protein precursor (PBP) | PM | [67] |
| slr1250 | phosphate transport ATP-binding protein PstB homolog | PM | [66] |
| slr1251 | peptidyl-prolyl cis-trans isomerase (cyp, rot1) | S | [27], [28], [62], [63], [64], [65], [66], [67] |
| slr1254 | phytoene desaturase | M, T | [5], [6], [67] |
| slr1255 | phytoene synthase | PS | [67] |
| slr1257 | unknown protein | PM | [66] |
| slr1258 | hypothetical protein | P | [8], [10], [67] |
| slr1259 | hypothetical protein | S | [27], [28], [62], [67] |
| slr1261 | hypothetical protein | PM | [67] |
| slr1263 | hypothetical protein | PM | [67] |
| slr1265 | RNA polymerase gamma-subunit | PS | [63], [65], [66], [67] |
| slr1267 | cell division protein FtsW | PM | [67] |
| slr1269 | gamma-glutamyltranspeptidase | PM | [66], [67] |
| slr1270 | TolC | M, O, P, PP | [C], [3], [6], [8], [55], [64], [67] |
| slr1272 | S-layer protein | M, O | [C], [3], [67] |
| slr1273 | hypothetical protein | PM | [27], [28], [63], [65], [66] |
| slr1274 | PilM | M, P | [C], [8], [10], [67] |
| slr1275 | PilN | M, P | [C], [8], [10], [67] |
| slr1276 | PilO | M, P | [C], [8], [67] |
| slr1277 | PilQ | M, O | [C], [3], [6], [67] |
| slr1280 | NADH dehydrogenase subunit, NdhK | M, T | [C], [5], [58], [67] |
| slr1281 | NADH dehydrogenase subunit I, NdhJ | M, T | [C], [5], [58], [67] |
| slr1287 | hypothetical protein | PM | [67] |
| slr1289 | isocitrate dehydrogenase (NADP+) | PM | [63], [65], [66], [67] |
| slr1295 | ABC transporter; Iron binding protein, FutA1 | M, O, P, T | [C], [3], [4], [5], [8], [10], [64], [66], [67] |
| slr1298 | unknown protein | PM | [66] |
| slr1299 | UDP-glucose dehydrogenase | PM | [66], [67] |
| slr1301 | hypothetical protein | PS | [64], [66], [67] |
| slr1302 | protein involved in constitutive low affinity CO2 uptake | PS | [67] |
| slr1305 | two-component response regulator | PS | [67] |
| slr1306 | hypothetical protein | PM | [67] |
| slr1311 | PSII D1 protein, PsbA2 | M, T | [5], [58], [63], [67] |
| slr1312 | arginine decarboxylase | PS | [66], [67] |
| slr1319 | iron(III) dicitrate transport system permease protein, FecB | P | [4], [8], [67] |
| slr1322 | putative modulator of DNA gyrase; TldD | PM | [63], [64], [66], [67] |
| slr1324 | two-component hybrid sensor and regulator | PS | [66] |
| slr1325 | GTP pyrophosphokinase | PS | [67] |
| slr1329 | ATP synthase beta subunit, AtpB | M, P, S, T | [C], [5], [6], [10], [27], [28], [58], [60], [61], [62], [63], [64], [65], [66], [67] |
| slr1330 | ATP synthase epsilon subunit, AtpE | M, P, S, T | [C], [5], [6], [10], [27], [28], [58], [60], [64] |
| slr1331 | processing protease | PP | [55], [64], [67] |
| slr1334 | phosphoglucomutase/phosphomannomutase | PS | [66], [67] |
| slr1338 | hypothetical protein | PS | [63], [66], [67] |
| slr1342 | hypothetical protein | PS | [63], [64], [66], [67] |
| slr1347 | β-type carbonic anhydrase | M | [C], [59], [66], [67] |
| slr1348 | serine-O-acetyl transferase | M | [59] |
| slr1349 | glucose-6-phosphate isomerase | PM | [63], [64], [65], [66], [67] |
| slr1350 | ATPase subunit epsilon | M | [6] |
| slr1351 | UDP-N-acetylmuramoylalanyl-D-glutamyl-2 6-diaminopimelate--D-alanyl-D-alanine ligase | PS | [66], [67] |
| slr1353 | hypothetical protein | PS | [63], [67] |
| slr1356 | 30S ribosomal protein S1 (rps1, rps1a) | M, S | [C], [6], [60], [61], [62], [63], [64], [65], [66], [67] |
| slr1363 | hypothetical protein | PS | [27], [28], [63], [67] |
| slr1364 | biotin synthetase | PS | [67] |
| slr1367 | glycogen phosphorylase | S, T | [56], [61], [63], [64], [66], [67] |
| slr1368 | precorrin decarbocylase | PS | [67] |
| slr1369 | phosphatidate cytidylyltransferase | PM | [67] |
| slr1377 | leader peptidase, LepB | P | [8], [10], [67] |
| slr1378 | hypothetical protein | PS | [66], [67] |
| slr1385 | unknown protein | PS | [66], [67] |
| slr1390 | protease, FtsH2 | P | [4], [66], [67] |
| slr1393 | phytochrome-like protein, two-component sensor histidine kinase | PS | [66], [67] |
| slr1395 | hypothetical protein | PS | [67] |
| slr1397 | unknown protein | PS | [67] |
| slr1398 | unknown protein | PM | [67] |
| slr1403 | unknown protein | PM | [66] |
| slr1406 | hypothetical protein | O, PP | [3], [27], [28], [55] |
| slr1407 | unknown protein | PP | [56], [67] |
| slr1409 | periplasmic WD-repeat protein | PP | [27], [28], [55], [67] |
| slr1410 | periplasmic WD-repeat protein | PP | [55], [56], [64], [66], [67] |
| slr1411 | hypothetical protein | PM | [67] |
| slr1413 | hypothetical protein | PM | [66] |
| slr1415 | hypothetical protein | PM | [67] |
| slr1416 | MorR protein | M, P | [C], [10], [66], [67] |
| slr1418 | dihydroorotate dehydrogenase | PM | [67] |
| slr1423 | UDP-N-acetylmuramate-alanine ligase | PM | [67] |
| slr1424 | UDP-N-acetylenolpyruvoylglucosamine reductase | PM | [63], [66], [67] |
| slr1426 | recombination protein RecR | PS | [67] |
| slr1428 | hypothetical protein | PM | [67] |
| slr1431 | hypothetical protein | PM | [67] |
| slr1434 | pyridine nucleotide transhydrogenase beta subunit | PM | [67] |
| slr1435 | PmbA protein homolog | PM | [64], [66], [67] |
| slr1437 | unknown protein | PS | [63] |
| slr1438 | hypothetical protein | PS | [63] |
| slr1448 | fructokinase | PM | [66], [67] |
| slr1449 | hypothetical protein | PM | [66] |
| slr1452 | sulfate transport system substrate-binding protein | PM | [66], [67] |
| slr1459 | phycobilisome core component | PS | [63], [65], [66], [67] |
| slr1461 | hypothetical protein | PM | [67] |
| slr1462 | hypothetical protein | PM | [66] |
| slr1463 | elongation factor EF-G 1 | C, S | [56], [60], [61], [62], [63], [64], [65], [66], [67] |
| slr1467 | precorrin isomerase | M | [59], [67] |
| slr1471 | hypothetical protein | PM | [67] |
| slr1474 | hypothetical protein | PS | [67] |
| slr1476 | aspartate carbamoyltransferase | PS | [66], [67] |
| slr1478 | hypothetical protein | PM | [67] |
| slr1485 | phosphatidylinositol phosphate kinaseH | PP | [55], [64], [66], [67] |
| slr1490 | ferrichrome-iron receptor | PM | [66] |
| slr1501 | probable acetyltransferase | PS | [67] |
| slr1503 | hypothetical protein | PM | [67] |
| slr1505 | unknown protein | PS | [67] |
| slr1506 | hypothetical protein | M, O, P | [C], [3], [8], [27], [28], [66], [67] |
| slr1507 | hypothetical protein | PS | [67] |
| slr1510 | fatty acid/phospholipids synthesis protein PlsX | T | [5], [66], [67] |
| slr1511 | 3-oxoacyl-[acyl-carrier-protein] synthase III | PM | [67] |
| slr1512 | sodium-dependent bicarbonate transporter, SbtA | M, P | [4], [58], [67] |
| slr1513 | hypothetical protein | P, T | [4], [5], [8], [63] |
| slr1516 | superoxide dismutase | S | [27], [28], [60], [63], [64], [65], [66], [67] |
| slr1517 | 3-isopropyl malate dehydrogenase | S | [60], [61], [63], [66], [67] |
| slr1519 | hypothetical protein | PS | [67] |
| slr1521 | GTP-binding protein | PM | [67] |
| slr1531 | signal recognition particle protein | PM | [67] |
| slr1534 | hypothetical protein | PS | [66], [67] |
| slr1535 | hypothetical protein | PS | [64], [67] |
| slr1536 | ATP-dependent DNA helicase RecQ | PM | [66] |
| slr1537 | unknown protein | PS | [64], [67] |
| slr1540 | hypothetical protein | S | [62], [64], [66], [67] |
| slr1541 | hypothetical protein | PS | [67] |
| slr1542 | 2-C-methyl-D-erythritol 2,4-cyclodiphosphate synthase | PS | [66], [67] |
| slr1550 | Lysyl-tRNA synthetase | T | [56], [64], [67] |
| slr1552 | unknown protein | PM | [66], [67] |
| slr1556 | 2-hydroxyacid dehydrogenase homolog | PM | [67] |
| slr1557 | hypothetical protein | PS | [63], [67] |
| slr1559 | shikimate 5-dehydrogenase | PM | [67] |
| slr1560 | histidyl tRNA synthetase | PS | [67] |
| slr1562 | glutaredoxin | PS | [66] |
| slr1563 | hypothetical protein | PS | [66], [67] |
| slr1566 | hypothetical protein | PM | [67] |
| slr1570 | hypothetical protein | PM | [67] |
| slr1571 | unknown protein | PM | [67] |
| slr1573 | hypothetical protein | PM | [67] |
| slr1575 | probable potassium efflux system | PM | [66] |
| slr1577 | hypothetical protein | M | [C], [67] |
| slr1579 | hypothetical protein | PM | [67] |
| slr1588 | two-component transcription regulator | PM | [67] |
| slr1590 | hypothetical protein | P, S | [10], [61], [63], [66], [67] |
| slr1594 | two-component response regulator PatA subfamily | PS | [66] |
| slr1596 | a protein in the cytoplasmic membrane involved in light-induced proton extrusion. | PM | [66], [67] |
| slr1597 | chromosome partitioning ATPase, ParA family | PM | [67] |
| slr1598 | lipoic acid synthetase | PM | [67] |
| slr1603 | hypothetical protein | PM | [67] |
| slr1604 | FtsH | M, P | [4], [59], [66], [67] |
| slr1608 | glucose dehydrogenase b | PP | [55] |
| slr1609 | long-chain-fatty-acid CoA ligase, FadD | P | [4], [67] |
| slr1610 | putative C-3 methyl transferase | PS | [67] |
| slr1611 | hypothetical protein | PS | [66] |
| slr1612 | hypothetical protein | PS | [67] |
| slr1613 | hypothetical protein | PS | [67] |
| slr1615 | perosamine synthetase | PM | [66], [67] |
| slr1616 | unknown protein | PM | [66], [67] |
| slr1617 | similar to UDP-glucose 4-epimerase | PS | [67] |
| slr1618 | unknown protein | PS | [67] |
| slr1619 | hypothetical protein | PM | [64], [66], [67] |
| slr1622 | soluble inorganic pyrophosphatase | PS | [27], [28], [63], [64], [65], [66], [67] |
| slr1623 | hypothetical protein | M, T | [C], [5], [27], [28], [67] |
| slr1624 | hypothetical protein | P | [8], [66], [67] |
| slr1626 | dihydroneopterin aldolase | PS | [63], [66], [67] |
| slr1628 | hypothetical protein | PS | [66] |
| slr1639 | SsrA-binding protein | PS | [66] |
| slr1641 | ClpB1 | M, S | [59], [62], [63], [65], [66], [67] |
| slr1643 | ferredoxin-NADP oxidoreductase (petH) | S, T | [5], [60], [62], [63], [64], [65], [66], [67] |
| slr1644 | hypothetical protein | PM | [67] |
| slr1645 | photosystem II 11 kD protein | PM | [67] |
| slr1647 | hypothetical protein | PM | [67] |
| slr1648 | hypothetical protein | PM | [67] |
| slr1649 | hypothetical protein | PP | [56], [63], [64], [65], [66], [67] |
| slr1651 | ABC transporter ATP-binding protein | PM | [66] |
| slr1655 | PSI subunit PsaL | M | [58], [67] |
| slr1656 | UDP-N-acetylglucosamine--N-acetylmuramyl-(pentapeptide) pyrophosphoryl -undecaprenol N-acetylglucosamine transferase | PM | [67] |
| slr1657 | hypothetical protein | PS | [67] |
| slr1658 | unknown protein | PS | [67] |
| slr1659 | hypothetical protein | PM | [66] |
| slr1661 | hypothetical protein | PM | [67] |
| slr1665 | diaminopimelate epimerase | PM | [64], [67] |
| slr1666 | pleiotropic regulatory protein homolog | PS | [67] |
| slr1668 | hypothetical protein | M, PP | [6], [27], [28], [55], [63], [67] |
| slr1672 | glycerol kinase | PS | [66] |
| slr1678 | 50S ribosomal protein L21 | PS | [63], [64], [67] |
| slr1679 | hypothetical protein | PS | [67] |
| slr1681 | unknown protein | PM | [66] |
| slr1686 | hypothetical protein | PS | [63], [67] |
| slr1687 | hypothetical protein | PS | [67] |
| slr1689 | formamidopyrimidine-DNA glycosylase | PS | [67] |
| slr1691 | glutamine-dependent NAD(+) synthetase | PS | [67] |
| slr1692 | hypothetical protein | PS | [67] |
| slr1693 | two-component response regulator PatA subfamily | PS | [67] |
| slr1694 | expression activator appA homolog | PS | [67] |
| slr1697 | serine/threonine kinase | PM | [67] |
| slr1699 | hypothetical protein | PM | [67] |
| slr1702 | hypothetical protein | M | [59], [67] |
| slr1703 | seryl-tRNA synthetase | PS | [64], [66], [67] |
| slr1710 | penicillin-binding protein | PM | [67] |
| slr1712 | hypothetical protein | PM | [66], [67] |
| slr1717 | hypothetical protein | PS | [67] |
| slr1718 | hypothetical protein | PS | [67] |
| slr1719 | protein DraG (draG) | S | [27], [28], [60], [62], [63], [64], [65], [66], [67] |
| slr1720 | Aspartyl-tRNA synthetase | M, S | [59], [61], [62], [64], [66], [67] |
| slr1721 | hypothetical protein | P | [4], [67] |
| slr1722 | IMP dehydrogenase | S | [60], [61], [63], [64], [65], [66], [67] |
| slr1727 | Na+/H+ antiporter | PM | [67] |
| slr1729 | potassium-transporting P-type ATPase B chain | PM | [66], [67] |
| slr1730 | potassium-transporting ATPase C chain, KdpC | P | [8] |
| slr1732 | hypothetical protein | PS | [27], [28], [64] |
| slr1734 | putative oxppcycle protein Opac | S | [61], [63], [64], [65], [66], [67] |
| slr1735 | ATP-binding subunit of the ABC-type Bgt permease for basic amino acids and glutamine | PS | [67] |
| slr1739 | PSII 13 kDa protein homolog | T | [5], [66] |
| slr1740 | ABC transporter; Oligopeptide binding protein | P | [10], [66], [67] |
| slr1742 | probable cobyric acid synthase | PS | [67] |
| slr1743 | type 2 NADH dehydrogenase NdbB | PS | [64], [67] |
| slr1744 | N-Acetylmuramoyl-l-alanine amidase | PP | [55], [67] |
| slr1748 | probable phosphoglycerate mutase | PS | [64] |
| slr1751 | carboxyl-terminal protease | O, P, PP, S, T | [3], [5], [8], [55],[56], [61], [64], [67] |
| slr1753 | hypothetical protein | O | [3], [63], [66], [67] |
| slr1755 | NAD+ dependent glycerol-3-phosphate dehydrogenase | PM | [67] |
| slr1756 | glutamate-ammonia ligase, glutamine synthetase type I (glnA) | S | [60], [61], [62], [63], [64], [65], [66], [67] |
| slr1759 | two-component hybrid sensor and regulator | PM | [66] |
| slr1761 | Fkbp-type peptidyl-prolyl cis±trans isomerase | PP | [27], [28], [55], [63], [64], [67] |
| slr1762 | hypothetical protein | PS | [67] |
| slr1763 | probable methyltransferase | PS | [63], [67] |
| slr1768 | prohibitin | P | [8], [10], [67] |
| slr1772 | choloylglycine hydrolaseH | PP | [55], [67] |
| slr1777 | magnesium protoporphyrin IX chelatase subunit D | PM | [66], [67] |
| slr1779 | pyridoxal phosphate biosynthetic protein PdxJ | PS | [63], [64], [67] |
| slr1780 | hypothetical protein YCF54 | PS | [63], [64], [65], [66], [67] |
| slr1783 | response regulator Rre1 | M | [59], [64], [66], [67] |
| slr1784 | biliverdin reductase | PS | [66], [67] |
| slr1788 | unknown protein | PS | [66] |
| slr1790 | hypothetical protein | PM | [67] |
| slr1791 | phosphoadenosine phosphosulfate reductase | PM | [67] |
| slr1793 | transaldolase | S | [60], [63], [64], [65], [66], [67] |
| slr1794 | hypothetical protein - probable anion transporting ATPase | S | [62], [64], [66], [67] |
| slr1795 | peptide methionine sulfoxide reductase | PM | [67] |
| slr1796 | hypothetical protein | T | [5] |
| slr1799 | hypothetical protein | PM | [67] |
| slr1800 | hypothetical protein | PS | [67] |
| slr1808 | transfer RNA-Gln reductase | PS | [66], [67] |
| slr1814 | hypothetical protein | PS | [67] |
| slr1815 | hypothetical protein | PS | [67] |
| slr1816 | hypothetical protein | PS | [67] |
| slr1819 | hypothetical protein | PS | [67] |
| slr1821 | hypothetical protein | PM | [66], [67] |
| slr1828 | ferredoxin, petF-like protein | PM | [63] |
| slr1829 | putative poly(3-hydroxyalkanoate) synthase component | PS | [67] |
| slr1834 | PSI subunit PsaA | M | [58], [63], [65], [66], [67] |
| slr1835 | PSI subunit PsaB | M, T | [5], [58], [63], [67] |
| slr1837 | two-component system response regulator OmpR subfamily | PS | [67] |
| slr1839 | carbon dioxide concentrating mechanism protein CcmK | M | [C], [27], [28], [63], [64], [65], [66] |
| slr1841 | putative porin | M, O, P | [C], [3], [4], [8], [10], [64], [66], [67] |
| slr1842 | cysteine synthase | PM | [27], [28], [64], [67] |
| slr1843 | glucose 6-phosphate dehydrogenase | PS | [63], [66], [67] |
| slr1844 | excinuclease ABC subunit A | PS | [67] |
| slr1848 | histidinol dehydrogenase | S | [60], [63], [66], [67] |
| slr1849 | probable mercuric reductase | PM | [66] |
| slr1852 | hypothetical protein | PP, S | [56], [60], [61], [63], [64], [65], [66], [67] |
| slr1853 | carboxymuconolactone decarboxylase | PS | [63] |
| slr1854 | hypothetical protein | S | [61], [63], [64], [66], [67] |
| slr1855 | hypothetical protein | S, T | [56], [61], [63], [64], [65], [66], [67] |
| slr1856 | phosphoprotein substrate of icfG gene cluster | PS | [63] |
| slr1857 | isoamylase | PM | [64], [66], [67] |
| slr1859 | anti-sigma f factor antagonist | PS | [63], [65] |
| slr1860 | carbon metabolisms regulatory protein IcfG | PM | [67] |
| slr1863 | unknown protein | PS | [66] |
| slr1867 | anthranilate phosphoribosyltransferase | PM | [67] |
| slr1874 | D-alanine--D-alanine ligase | PM | [66], [67] |
| slr1875 | hypothetical protein | PM | [67] |
| slr1877 | 2-hydroxyhepta-2,4-diene-1,7-dioate isomerase | PS | [67] |
| slr1878 | phycocyanin alpha-subunit phycocyanobilin lyase | PS | [67] |
| slr1880 | hypothetical protein | PS | [67] |
| slr1881 | high affinity branched-chain amino acid transport ATP-binding | P | [8], [67] |
| slr1882 | riboflavin biosynthesis protein RibF | PS | [66], [67] |
| slr1884 | tryptophanyl-tRNA synthetase | PS | [67] |
| slr1887 | porphobilinogen deaminase (hydroxymethylbilane synthase, preuroporphyrinogen synthase) | PS | [27], [28], [64], [66], [67] |
| slr1888 | 4-hydroxybutyrate coenzyme A transferase. | PS | [67] |
| slr1890 | bacterioferritin | PS | [63], [65], [66], [67] |
| slr1894 | probable DNA-binding stress protein | PS | [27], [28], [63], [64], [65], [66], [67] |
| slr1897 | ABC transporter; Sugar binding protein, SrrA | P | [8], [10], [66], [67] |
| slr1898 | N-acetylglutamate kinase | PS | [66], [67] |
| slr1899 | urease accessory protein F | S | [27], [28] |
| slr1900 | hypothetical protein | PS | [64], [67] |
| slr1901 | ATP-binding protein of ABC transporter | PM | [67] |
| slr1906 | hypothetical protein | PS | [67] |
| slr1908 | putative porin | M, O, P | [C], [3], [4], [8], [10], [64], [67] |
| slr1909 | NarL subfamily | M | [C], [27], [28], [67] |
| slr1916 | probable esterase | PM | [67] |
| slr1918 | hypothetical protein | PM | [67] |
| slr1919 | hypothetical protein | PM | [67] |
| slr1920 | unknown protein | PM | [67] |
| slr1923 | hypothetical protein | PM | [67] |
| slr1924 | d-Alanyl-d-alanine carboxypeptidase | PP | [55], [63], [66], [67] |
| slr1925 | cobalamin biosynthesis protein CobD | PM | [63] |
| slr1933 | dTDP-4-dehydrorhamnose 3,5-epimerase | PS | [64], [67] |
| slr1934 | pyruvate dehydrogenase component E1, a-subunit | M, S, T | [5], [59], [62], [63], [64], [65], [66], [67] |
| slr1938 | putative translation initiation factor EIF-2b subunit 1 | PM | [64], [66], [67] |
| slr1939 | unknown protein | PM | [66], [67] |
| slr1940 | Collagen-like proteinH | PP | [55], [63], [67] |
| slr1942 | circadian clock protein KaiC homolog | PS | [66], [67] |
| slr1943 | putative glycosyltransferase | P | [4] |
| slr1944 | hypothetical protein | PP | [55], [56], [66], [67] |
| slr1945 | 2,3-bisphosphoglycerate-independent phosphoglycerate mutase | PS | [64], [66], [67] |
| slr1949 | hypothetical protein | T | [5], [67] |
| slr1950 | copper-transporting P-type ATPase CtaA | PM | [67] |
| slr1951 | hypothetical protein | PS | [67] |
| slr1956 | unknown protein | PP | [56] |
| slr1958 | unknown protein | PS | [67] |
| slr1959 | unknown protein | PS | [66], [67] |
| slr1962 | probable extracellular solute-binding protein | PP | [56], [67] |
| slr1963 | water-soluble carotenoid protein | S, T | [5], [60], [61], [62], [63], [64], [65], [66], [67] |
| slr1968 | hypothetical protein | S | [61], [66], [67] |
| slr1969 | two-component sensor histidine kinase | PS | [67] |
| slr1970 | hypothetical protein | PS | [66], [67] |
| slr1974 | GTP binding protein | PS | [67] |
| slr1975 | N-acylglucosamine 2-epimerase | PS | [67] |
| slr1978 | hypothetical protein | PM | [67] |
| slr1983 | two-component hybrid sensor and regulator | PM | [67] |
| slr1984 | 30S ribosomal protein S1 (rps1, rps1a) | S | [62], [63], [64], [65], [66], [67] |
| slr1986 | allophycocyanin beta subunit | M, S | [C], [6], [27], [28], [61], [63], [64], [65], [66], [67] |
| slr1992 | glutathione peroxidase-like NADPH peroxidase | PS | [27], [28], [63], [64], [65], [66], [67] |
| slr1993 | PHA-specific beta-ketothiolase | PM | [64], [66], [67] |
| slr1994 | PHA-specific acetoacetyl-CoA reductase (phaB, fabG2) | S | [60], [62], [64], [66], [67] |
| slr1998 | hypothetical protein | PS | [66], [67] |
| slr2000 | hypothetical protein | P | [10], [67] |
| slr2001 | cyanophycinase | PM | [63], [64], [66], [67] |
| slr2002 | cyanophycin synthetase | PM | [63], [65], [66], [67] |
| slr2004 | hypothetical protein | PP | [55], [67] |
| slr2005 | hypothetical protein | PP | [55], [64], [66], [67] |
| slr2011 | hypothetical protein | PM | [67] |
| slr2015 | type 4 pilin-like protein, essential for motility | PM | [67] |
| slr2017 | type 4 pilin-like protein, essential for motility | PM | [67] |
| slr2018 | unknown protein | PM | [66], [67] |
| slr2019 | ATP-binding protein of ABC transporter | PM | [66], [67] |
| slr2023 | malonyl coenzyme A-acyl carrier protein transacylase | PS | [67] |
| slr2024 | CheY superfamily | M, P, S | [C], [6], [10], [62], [63], [67] |
| slr2025 | hypothetical protein | PS | [63], [64], [65], [67] |
| slr2032 | hypothetical protein YCF23 | PM | [27], [28], [66], [67] |
| slr2034 | putative homolog of plant HCF136 | M | [C], [67] |
| slr2035 | glutamate 5-kinase | PS | [67] |
| slr2044 | zinc transport system ATP-binding protein | PS | [67] |
| slr2047 | PhoH like protein | PM | [67] |
| slr2048 | UDP-N-acetylglucosamine transferaseH | PP | [55], [64], [67] |
| slr2049 | hypothetical protein YCF58 | PS | [67] |
| slr2051 | phycobilisome rod-core linker polypeptide (cpcG1) | S, T | [5], [27], [28], [62], [63], [65], [66], [67] |
| slr2053 | putative hydrolase | PM | [67] |
| slr2058 | DNA topoisomerase | S | [61], [66], [67] |
| slr2060 | hypothetical protein | PS | [67] |
| slr2067 | allophycocyanin alpha subunit | M, P, S | [C], [10], [27], [28], [58], [61], [63], [64], [65], [66], [67] |
| slr2070 | hypothetical protein | PS | [64], [67] |
| slr2072 | L-threonine deaminase | PM | [64], [67] |
| slr2073 | hypothetical protein ycf50 | M | [59], [67] |
| slr2075 | 10 kDa chaperone (groES) | C, S | [27], [28], [56], [60], [62], [63], [64], [65], [66], [67] |
| slr2076 | 60 kDa chaperonin 1 (GroEL 1) | C, M, S, T | [5],[56], [27], [28], [59], [60], [61], [62], [63], [64], [65], [66], [67] |
| slr2079 | putative glutaminase | PM | [67] |
| slr2081 | prephenate dehydrogenase | PM | [67] |
| slr2087 | c-type cytochrome biogenesis protein Ccs1 | PM | [66], [67] |
| slr2088 | acetohydroxy acid synthase | PM | [63], [64], [66], [67] |
| slr2089 | squalene-hopene-cyclase | PS | [63], [65], [67] |
| slr2094 | fructose-1,6-/sedoheptulose-1,7-bisphosphatase (fbpI, glpX) | C, S | [27], [28], [56], [60], [61], [62], [63], [64], [65], [66], [67] |
| slr2098 | two-component hybrid sensor and regulator | PM | [66] |
| slr2100 | two-component response regulator | PS | [67] |
| slr2101 | hypothetical protein | PM | [27], [28], [63] |
| slr2102 | cell division protein FtsY | PS | [64], [67] |
| slr2104 | two-component hybrid sensor and regulator | PM | [66] |
| slr2105 | hypothetical protein | P | [4], [67] |
| slr2115 | unknown protein | PS | [66] |
| slr2116 | probable glycosyltransferase | PS | [67] |
| slr2122 | hypothetical protein | PS | [67] |
| slr2123 | similar to D-3-phosphoglycerate dehydrogenase | PS | [66], [67] |
| slr2130 | 3-dehydroquinate synthase | PM | [63], [64], [67] |
| slr2131 | Cation/multidrug efflux system protein | P | [4], [66], [67] |
| slr2132 | phosphotransacetylase | PM | [66], [67] |
| slr2136 | GCPE | S | [61], [64], [66], [67] |
| slr2141 | hypothetical protein | PS | [67] |
| slr2143 | L-cysteine/cystine lyase | PS | [66], [67] |
| slr2144 | hypothetical protein | PP | [55], [64], [67] |
| slr6071 | hypothetical protein | P | [4] |
| slr7094 | hypothetical protein | PS | [66] |
| sml0006 | 50S ribosomal protein L36 | PS | [67] |
| ssl0020 | ferredoxin I, essential for growth | PS | [27], [28], [67] |
| ssl0352 | hypothetical protein | M | [6], [27], [28], [63] |
| ssl0563 | PSI subunit PsaC | M, P, S, T | [C], [5], [8], [27], [28], [62] |
| ssl0707 | nitrogen regulatory protein P-II, GlnB | M, P, S | [C], [6], [8], [10], [27], [28], [60], [63], [64], [65], [66], [67] |
| ssl0787 | unknown protein | PS | [67] |
| ssl0788 | hypothetical protein | PM | [67] |
| ssl1690 | hypothetical protein | M, T | [C], [5], [6], [27], [28] |
| ssl1972 | hypothetical protein | PS | [27], [28], [63] |
| ssl2009 | hypothetical protein | T | [5] |
| ssl2064 | hypothetical protein | PS | [63] |
| ssl2084 | acyl carrier protein | PS | [63], [65], [67] |
| ssl2296 | pterin-4a-carbinolamine dehydratase | PS | [27], [28], [63], [65] |
| ssl2501 | hypothetical protein | M, T | [5], [6] |
| ssl2595 | hypothetical protein | PS | [66] |
| ssl2598 | photosystem II PsbH protein | PM | [63] |
| ssl2781 | hypothetical protein | M | [6], [27], [28], [63] |
| ssl2874 | hypothetical protein | PS | [66] |
| ssl2982 | probable DNA-directed RNA polymerase omega subunit | PS | [63] |
| ssl2999 | hypothetical protein | PS | [66] |
| ssl3093 | phycocyanin associated linker protein | M | [6], [27], [28], [63], [65], [66], [67] |
| ssl3364 | CP12 polypeptide | PS | [67] |
| ssl3436 | 50S ribosomal protein L29 | PS | [27], [28], [63], [65] |
| ssl3441 | initiation factor IF-1 | PS | [63] |
| ssl3445 | 50S ribosomal protein L31 | PS | [63], [66] |
| ssr0330 | ferredoxin-thioredoxin reductase, variable chain | PS | [27], [28], [63], [66] |
| ssr0482 | 30S ribosomal protein S16 | PS | [66] |
| ssr1399 | 30S ribosomal protein S18 | PS | [63] |
| ssr1480 | putative RNA-binding protein | PS | [27], [28], [63], [64] |
| ssr1528 | hypothetical protein | PS | [27], [28], [63], [65] |
| ssr1600 | similar to anti-sigma f factor antagonist | PS | [27], [28], [63], [65], [66], [67] |
| ssr1698 | hypothetical protein | PS | [27], [28], [66] |
| ssr1853 | unknown protein | PM | [63] |
| ssr2061 | glutaredoxin | S | [27], [28] |
| ssr2422 | hypothetical protein | T | [5], [66] |
| ssr2553 | unknown protein | PS | [66] |
| ssr2755 | hypothetical protein | PS | [66] |
| ssr2787 | unknown protein | PS | [66] |
| ssr2799 | 50S ribosomal protein L27 | PS | [66], [67] |
| ssr2831 | PSI subunit PsaE | M, T | [C], [5], [6], [27], [28], [63], [66], [67] |
| ssr2857 | mercuric transport protein periplasmic component precursor | PS | [66] |
| ssr2998 | hypothetical protein | M | [C], [27], [28], [63], [65] |
| ssr3122 | hypothetical protein | PS | [66] |
| ssr3383 | phycobilisome small core linker polypeptide | PS | [63], [65], [66], [67] |
| ssr3451 | cytochrome b559 subunit | M | [58], [67] |
| ssr3532 | hypothetical protein | M | [6] |
| ^†^In this column, C, M, O, P, PP, S, T, PM and PS represent the cytoplasm, membrane, outer membrane, plasma membrane, periplasma, soluble, thylakoid membrane, predicted membrane, and predicted soluble respectively | | | |
| ^‡^In this column, [C] represents the current work. | | | |
