# Supplementary material for: Identification and bioinformatic analysis of the membrane proteins of synechocystis sp. PCC 6803
Source: Proteome Sci. 2009 Mar 25;7:11. doi: 10.1186/1477-5956-7-11 (PMC2666656; doi:10.1186/1477-5956-7-11)
Supplement: Additional file 5 — The proteins of synechocystis sp. PCC 6803 not identified yet by large-scale proteomic approaches. Additional file 5 is a MS word table containing 1,935 proteins not identified based on the references mentioned in the text. [file 1477-5956-7-11-S5.doc]

| **Additional file 5. The proteins of *Synechocystis* sp. PCC 6803 not identified yet by large-scale proteomic approaches** | | | |
| --- | --- | --- | --- |
| **ORF** | **Gene Product** | **Gene Category** | **Gene Sub-category** |
| sgl0001 | hypothetical protein | Hypothetical | N/A |
| sgl0002 | hypothetical protein | Hypothetical | N/A |
| sll0007 | hypothetical protein | Hypothetical | N/A |
| sll0008 | unknown protein | Unknown | N/A |
| sll0010 | unknown protein | Unknown | N/A |
| sll0012 | putative transposase [ISY523f: 2482725 - 2483595] | Other categories | Transposon-related functions |
| sll0021 | probable exonuclease | DNA replication, restriction, modification, recombination, and repair | N/A |
| sll0022 | unknown protein | Unknown | N/A |
| sll0024 | unknown protein | Unknown | N/A |
| sll0026 | NADH dehydrogenase subunit 5 (involved in constitutive, low affinity CO2 uptake) | Photosynthesis and respiration | NADH dehydrogenase |
| sll0027 | NADH dehydrogenase subunit 4 (involved in constitutive, low affinity CO2 uptake) | Photosynthesis and respiration | NADH dehydrogenase |
| sll0030 | cmp operon transcriptional regulator, LysR family protein | Regulatory functions | N/A |
| sll0031 | hypothetical protein | Hypothetical | N/A |
| sll0039 | positive phototaxis protein, two-component response regulator CheY subfamily | Regulatory functions | N/A |
| sll0040 | positive phototaxis protein, homologous to chemotaxis protein CheW | Cellular processes | Chemotaxis |
| sll0042 | methyl-accepting chemotaxis protein for positive phototaxis | Cellular processes | Chemotaxis |
| sll0047 | hypothetical protein YCF12 | Hypothetical | N/A |
| sll0060 | hypothetical protein | Hypothetical | N/A |
| sll0062 | hypothetical protein | Hypothetical | N/A |
| sll0063 | hypothetical protein | Hypothetical | N/A |
| sll0072 | hypothetical protein | Hypothetical | N/A |
| sll0082 | hypothetical protein | Hypothetical | N/A |
| sll0084 | putative phosphatase | Amino acid biosynthesis | Aromatic amino acid family |
| sll0088 | hypothetical protein | Hypothetical | N/A |
| sll0092 | putative transposase [ISY391c: 2997600 - 2998989] | Other categories | Transposon-related functions |
| sll0094 | two-component sensor histidine kinase | Regulatory functions | N/A |
| sll0095 | hypothetical protein | Hypothetical | N/A |
| sll0099 | precorrin-6y C5, 15-methyltransferase (decarboxylating) | Biosynthesis of cofactors, prosthetic groups, and carriers | Cobalamin, heme, phycobilin and porphyrin |
| sll0101 | unknown protein | Unknown | N/A |
| sll0107 | KHG/KDPG aldolase | Energy metabolism | Amino acids and amines |
| sll0109 | chorismate mutase | Amino acid biosynthesis | Aromatic amino acid family |
| sll0140 | unknown protein | Unknown | N/A |
| sll0146 | Integral membrane protein of the ABC-type, Nat permease for neutral amino acids | Transport and binding proteins | N/A |
| sll0156 | unknown protein | Unknown | N/A |
| sll0157 | hypothetical protein | Hypothetical | N/A |
| sll0161 | putative transposase [ISY523p: 2328180 - 2329050] | Other categories | Transposon-related functions |
| sll0168 | hypothetical protein | Hypothetical | N/A |
| sll0174 | hypothetical protein | Hypothetical | N/A |
| sll0176 | hypothetical protein | Hypothetical | N/A |
| sll0177 | hypothetical protein | Hypothetical | N/A |
| sll0181 | unknown protein | Unknown | N/A |
| sll0183 | hypothetical protein | Hypothetical | N/A |
| sll0189 | hypothetical protein | Hypothetical | N/A |
| sll0191 | unknown protein | Unknown | N/A |
| sll0192 | hypothetical protein | Hypothetical | N/A |
| sll0194 | putative sec-independent protein translocase | Cellular processes | Protein and peptide secretion |
| sll0198 | hypothetical protein | Hypothetical | N/A |
| sll0200 | putative transposase [ISY100s: 2524547 - 2525492] | Other categories | Transposon-related functions |
| sll0201 | putative transposase [ISY100s: 2524547 - 2525492] | Other categories | Transposon-related functions |
| sll0205 | hypothetical protein | Hypothetical | N/A |
| sll0217 | flavoprotein | Other categories | Other |
| sll0218 | hypothetical protein | Hypothetical | N/A |
| sll0219 | flavoprotein | Other categories | Other |
| sll0221 | bacterioferritin comigratory protein | Transport and binding proteins | N/A |
| sll0222 | putative purple acid phosphatase | Other categories | Other |
| sll0223 | NADH dehydrogenase subunit 2 | Photosynthesis and respiration | NADH dehydrogenase |
| sll0225 | unknown protein | Unknown | N/A |
| sll0237 | unknown protein | Unknown | N/A |
| sll0238 | unknown protein | Unknown | N/A |
| sll0241 | unknown protein | Unknown | N/A |
| sll0243 | unknown protein | Unknown | N/A |
| sll0249 | hypothetical protein | Hypothetical | N/A |
| sll0253 | hypothetical protein | Hypothetical | N/A |
| sll0257 | hypothetical protein | Hypothetical | N/A |
| sll0261 | hypothetical protein | Hypothetical | N/A |
| sll0263 | unknown protein | Unknown | N/A |
| sll0264 | probable dioxygenase Rieske iron-sulfur component | Other categories | Other |
| sll0265 | unknown protein | Unknown | N/A |
| sll0266 | unknown protein | Unknown | N/A |
| sll0268 | hypothetical protein | Hypothetical | N/A |
| sll0269 | hypothetical protein | Hypothetical | N/A |
| sll0280 | unknown protein | Unknown | N/A |
| sll0281 | unknown protein | Unknown | N/A |
| sll0282 | unknown protein | Unknown | N/A |
| sll0284 | hypothetical protein | Hypothetical | N/A |
| sll0286 | hypothetical protein YCF52 | Hypothetical | N/A |
| sll0288 | septum site-determining protein MinC | Cellular processes | Cell division |
| sll0294 | hypothetical protein | Hypothetical | N/A |
| sll0295 | hypothetical protein | Hypothetical | N/A |
| sll0296 | hypothetical protein | Hypothetical | N/A |
| sll0297 | hypothetical protein | Hypothetical | N/A |
| sll0298 | hypothetical protein | Hypothetical | N/A |
| sll0300 | riboflavin synthase alpha chain | Biosynthesis of cofactors, prosthetic groups, and carriers | Riboflavin |
| sll0309 | unknown protein | Unknown | N/A |
| sll0310 | hypothetical protein | Hypothetical | N/A |
| sll0315 | putative transposase [ISY203i: 2443391 - 2443924, join 2444874 - 2445513] | Other categories | Transposon-related functions |
| sll0317 | putative transposase [ISY203i: 2443391 - 2443924, join 2444874 - 2445513] | Other categories | Transposon-related functions |
| sll0321 | unknown protein | Unknown | N/A |
| sll0327 | unknown protein | Unknown | N/A |
| sll0328 | unknown protein | Unknown | N/A |
| sll0330 | sepiapterine reductase | Fatty acid, phospholipid and sterol metabolism | N/A |
| sll0354 | hypothetical protein | Hypothetical | N/A |
| sll0355 | hypothetical protein | Hypothetical | N/A |
| sll0360 | hypothetical protein | Hypothetical | N/A |
| sll0361 | hypothetical protein | Hypothetical | N/A |
| sll0364 | hypothetical protein | Hypothetical | N/A |
| sll0369 | unknown protein | Unknown | N/A |
| sll0371 | unknown protein | Unknown | N/A |
| sll0372 | hypothetical protein | Hypothetical | N/A |
| sll0375 | unknown protein | Unknown | N/A |
| sll0382 | hypothetical protein | Hypothetical | N/A |
| sll0383 | cobalamin biosynthesis protein M | Biosynthesis of cofactors, prosthetic groups, and carriers | Cobalamin, heme, phycobilin and porphyrin |
| sll0385 | ATP-binding protein of ABC transporter | Transport and binding proteins | N/A |
| sll0394 | unknown protein | Unknown | N/A |
| sll0397 | hypothetical protein | Hypothetical | N/A |
| sll0398 | deoxyguanosinetriphosphate triphosphohydrolase | Purines, pyrimidines, nucleosides, and nucleotides | Purine ribonucleotide biosynthesis |
| sll0400 | hypothetical protein | Hypothetical | N/A |
| sll0403 | unknown protein | Unknown | N/A |
| sll0406 | unknown protein | Unknown | N/A |
| sll0409 | similar to O-succinylbenzoate-CoA synthase | Biosynthesis of cofactors, prosthetic groups, and carriers | Menaquinone and ubiquinone |
| sll0419 | unknown protein | Unknown | N/A |
| sll0423 | hypothetical protein | Hypothetical | N/A |
| sll0426 | unknown protein | Unknown | N/A |
| sll0428 | unknown protein | Unknown | N/A |
| sll0431 | putative transposase [ISY100h: 3512289 - 3513235] | Other categories | Transposon-related functions |
| sll0436 | hypothetical protein | Hypothetical | N/A |
| sll0442 | hypothetical protein | Hypothetical | N/A |
| sll0444 | unknown protein | Unknown | N/A |
| sll0447 | unknown protein | Unknown | N/A |
| sll0448 | unknown protein | Unknown | N/A |
| sll0449 | unknown protein | Unknown | N/A |
| sll0450 | cytochrome b subunit of nitric oxide reductase | Amino acid biosynthesis | Glutamate family / Nitrogen assimilation |
| sll0451 | hypothetical protein | Hypothetical | N/A |
| sll0462 | hypothetical protein | Hypothetical | N/A |
| sll0473 | unknown protein | Unknown | N/A |
| sll0478 | unknown protein | Unknown | N/A |
| sll0479 | unknown protein | Unknown | N/A |
| sll0481 | unknown protein | Unknown | N/A |
| sll0484 | ATP-binding protein of ABC transporter | Transport and binding proteins | N/A |
| sll0485 | two-component response regulator NarL subfamily | Regulatory functions | N/A |
| sll0486 | circadian clock protein KaiB homolog | Other categories | Other |
| sll0488 | hypothetical protein | Hypothetical | N/A |
| sll0494 | unknown protein | Unknown | N/A |
| sll0496 | hypothetical protein | Hypothetical | N/A |
| sll0498 | hypothetical protein | Hypothetical | N/A |
| sll0508 | unknown protein | Unknown | N/A |
| sll0517 | putative RNA binding protein | Translation | Nucleoproteins |
| sll0522 | NADH dehydrogenase subunit 4L | Photosynthesis and respiration | NADH dehydrogenase |
| sll0525 | hypothetical protein | Hypothetical | N/A |
| sll0532 | hypothetical protein | Hypothetical | N/A |
| sll0536 | probable potassium channel protein | Transport and binding proteins | N/A |
| sll0537 | ammonium/methylammonium permease | Transport and binding proteins | N/A |
| sll0543 | hypothetical protein | Hypothetical | N/A |
| sll0544 | hypothetical protein | Hypothetical | N/A |
| sll0546 | probable translation initiation factor | Translation | Protein modification and translation factors |
| sll0547 | unknown protein | Unknown | N/A |
| sll0549 | hypothetical protein | Hypothetical | N/A |
| sll0552 | unknown protein | Unknown | N/A |
| sll0564 | hypothetical protein | Hypothetical | N/A |
| sll0577 | hypothetical protein | Hypothetical | N/A |
| sll0584 | hypothetical protein YCF36 | Hypothetical | N/A |
| sll0586 | hypothetical protein | Hypothetical | N/A |
| sll0590 | unknown protein | Unknown | N/A |
| sll0595 | unknown protein | Unknown | N/A |
| sll0597 | hypothetical protein | Hypothetical | N/A |
| sll0603 | menaquinone biosynthesis protein MenD | Biosynthesis of cofactors, prosthetic groups, and carriers | Menaquinone and ubiquinone |
| sll0608 | hypothetical protein YCF49 | Hypothetical | N/A |
| sll0609 | hypothetical protein | Hypothetical | N/A |
| sll0611 | hypothetical protein | Hypothetical | N/A |
| sll0613 | holliday junction DNA helicase RuvB | DNA replication, restriction, modification, recombination, and repair | N/A |
| sll0614 | unknown protein | Unknown | N/A |
| sll0621 | putative c-type cytochrome biogenesis protein CcdA | Other categories | Other |
| sll0623 | unknown protein | Unknown | N/A |
| sll0624 | unknown protein | Unknown | N/A |
| sll0629 | alternative photosystem I reaction center subunit X | Photosynthesis and respiration | Photosystem I |
| sll0640 | probable sodium/sulfate symporter | Transport and binding proteins | N/A |
| sll0641 | unknown protein | Unknown | N/A |
| sll0643 | urease accessory protein G | Central intermediary metabolism | Other |
| sll0647 | unknown protein | Unknown | N/A |
| sll0648 | probable glycosyltransferase | Other categories | Drug and analog sensitivity |
| sll0649 | two-component response regulator OmpR subfamily | Regulatory functions | N/A |
| sll0650 | putative transposase [ISY100j: 421739 - 422684] | Other categories | Transposon-related functions |
| sll0651 | putative transposase [ISY100j: 421739 - 422684] | Other categories | Transposon-related functions |
| sll0656 | unknown protein | Unknown | N/A |
| sll0658 | hypothetical protein | Hypothetical | N/A |
| sll0661 | hypothetical protein YCF35 | Hypothetical | N/A |
| sll0662 | 4Fe-4S type iron-sulfur protein | Hypothetical | N/A |
| sll0665 | putative transposase [ISY523r: 3109761 - 3110626] | Other categories | Transposon-related functions |
| sll0666 | putative transposase [ISY523r: 3109761 - 3110626] | Other categories | Transposon-related functions |
| sll0667 | putative transposase [ISY352e: 2921301 - 2921595, join 3108631 - 3109754] | Other categories | Transposon-related functions |
| sll0668 | putative transposase [ISY352e: 2921301 - 2921595, join 3108631 - 3109754] | Other categories | Transposon-related functions |
| sll0669 | unknown protein | Unknown | N/A |
| sll0670 | hypothetical protein | Hypothetical | N/A |
| sll0671 | probable cation transporter | Transport and binding proteins | N/A |
| sll0676 | hypothetical protein | Hypothetical | N/A |
| sll0677 | putative transposase [ISY523h: 3093889 - 3094759] | Other categories | Transposon-related functions |
| sll0678 | hypothetical protein | Hypothetical | N/A |
| sll0682 | phosphate transport system permease protein PstA homolog | Transport and binding proteins | N/A |
| sll0685 | hypothetical protein | Hypothetical | N/A |
| sll0686 | probable cytochrome c-type biogenesis protein | Other categories | Other |
| sll0687 | RNA polymerase ECF-type (group 3) sigma factor | Transcription | RNA synthesis, modification, and DNA transcription |
| sll0688 | unknown protein | Unknown | N/A |
| sll0690 | probable transcription regulator | Regulatory functions | N/A |
| sll0691 | hypothetical protein | Hypothetical | N/A |
| sll0696 | hypothetical protein | Hypothetical | N/A |
| sll0699 | putative transposase [ISY100i: 123475 - 124420] | Other categories | Transposon-related functions |
| sll0700 | putative transposase [ISY100i: 123475 - 124420] | Other categories | Transposon-related functions |
| sll0702 | unknown protein | Unknown | N/A |
| sll0704 | cysteine desulfurase | Amino acid biosynthesis | Glutamate family / Nitrogen assimilation |
| sll0710 | unknown protein | Unknown | N/A |
| sll0720 | RTX toxin activating protein homolog | Cellular processes | Cell killing |
| sll0722 | unknown protein | Unknown | N/A |
| sll0727 | hypothetical protein | Hypothetical | N/A |
| sll0732 | hypothetical protein | Hypothetical | N/A |
| sll0733 | unknown protein | Unknown | N/A |
| sll0736 | hypothetical protein | Hypothetical | N/A |
| sll0742 | hypothetical protein | Hypothetical | N/A |
| sll0743 | hypothetical protein | Hypothetical | N/A |
| sll0744 | hypothetical protein | Hypothetical | N/A |
| sll0760 | hypothetical protein YCF38 | Hypothetical | N/A |
| sll0761 | unknown protein | Unknown | N/A |
| sll0762 | unknown protein | Unknown | N/A |
| sll0763 | hypothetical protein | Hypothetical | N/A |
| sll0775 | unknown protein | Unknown | N/A |
| sll0776 | serine/threonine kinase | Regulatory functions | N/A |
| sll0777 | putative carboxypeptidase | Other categories | Other |
| sll0778 | ABC transporter, ATP-binding protein | Transport and binding proteins | N/A |
| sll0780 | unknown protein | Unknown | N/A |
| sll0782 | transcriptional regulator | Regulatory functions | N/A |
| sll0784 | nitrilase | Amino acid biosynthesis | Glutamate family / Nitrogen assimilation |
| sll0785 | unknown protein | Unknown | N/A |
| sll0786 | unknown protein | Unknown | N/A |
| sll0787 | hypothetical protein | Hypothetical | N/A |
| sll0788 | hypothetical protein | Hypothetical | N/A |
| sll0789 | two-component response regulator OmpR subfamily | Regulatory functions | N/A |
| sll0793 | hypothetical protein | Hypothetical | N/A |
| sll0797 | redox-responsive and/or Ni(II)-responsive regulator, two-component response regulator OmpR subfamily | Regulatory functions | N/A |
| sll0798 | Ni(II)-sensor and/or redox sensor, two-component sensor histidine kinase | Regulatory functions | N/A |
| sll0800 | hypothetical protein | Hypothetical | N/A |
| sll0802 | hypothetical protein | Hypothetical | N/A |
| sll0803 | hypothetical protein | Hypothetical | N/A |
| sll0804 | hypothetical protein | Hypothetical | N/A |
| sll0808 | putative transposase [ISY508a: 1710788 - 1711753] | Other categories | Transposon-related functions |
| sll0809 | hypothetical protein | Hypothetical | N/A |
| sll0810 | unknown protein | Unknown | N/A |
| sll0811 | unknown protein | Unknown | N/A |
| sll0812 | hypothetical protein | Hypothetical | N/A |
| sll0818 | tetrapyrrole methylase family protein | Other categories | Other |
| sll0823 | probable succinate dehydrogenase iron-sulfur protein | Energy metabolism | TCA cycle |
| sll0832 | hypothetical protein | Hypothetical | N/A |
| sll0833 | probable oligopeptides ABC transporter permease protein | Transport and binding proteins | N/A |
| sll0839 | hypothetical protein | Hypothetical | N/A |
| sll0843 | unknown protein | Unknown | N/A |
| sll0846 | hypothetical protein | Hypothetical | N/A |
| sll0847 | unknown protein | Unknown | N/A |
| sll0855 | putative channel transporter | Transport and binding proteins | N/A |
| sll0856 | RNA polymerase ECF-type (group 3) sigma-E factor | Transcription | RNA synthesis, modification, and DNA transcription |
| sll0857 | unknown protein | Unknown | N/A |
| sll0858 | hypothetical protein | Hypothetical | N/A |
| sll0862 | hypothetical protein | Hypothetical | N/A |
| sll0863 | hypothetical protein | Hypothetical | N/A |
| sll0864 | hypothetical protein | Hypothetical | N/A |
| sll0865 | excinuclease ABC subunit C | DNA replication, restriction, modification, recombination, and repair | N/A |
| sll0867 | hypothetical protein | Hypothetical | N/A |
| sll0868 | lipoic acid synthetase | Biosynthesis of cofactors, prosthetic groups, and carriers | Lipoate |
| sll0869 | Leu/Phe-tRNA-protein transferase | Translation | Protein modification and translation factors |
| sll0871 | hypothetical protein | Hypothetical | N/A |
| sll0888 | hypothetical protein | Hypothetical | N/A |
| sll0892 | aspartate 1-decarboxylase | Biosynthesis of cofactors, prosthetic groups, and carriers | Pantothenate |
| sll0896 | holliday juction resolvase RuvC | DNA replication, restriction, modification, recombination, and repair | N/A |
| sll0905 | hypothetical protein | Hypothetical | N/A |
| sll0909 | unknown protein | Unknown | N/A |
| sll0911 | unknown protein | Unknown | N/A |
| sll0913 | hypothetical protein | Hypothetical | N/A |
| sll0916 | precorrin isomerase, precorrin-8X methylmutase | Biosynthesis of cofactors, prosthetic groups, and carriers | Cobalamin, heme, phycobilin and porphyrin |
| sll0922 | unknown protein | Unknown | N/A |
| sll0924 | hypothetical protein | Hypothetical | N/A |
| sll0926 | hypothetical protein | Hypothetical | N/A |
| sll0930 | unknown protein | Unknown | N/A |
| sll0932 | hypothetical protein | Hypothetical | N/A |
| sll0939 | hypothetical protein | Hypothetical | N/A |
| sll0943 | unknown protein | Unknown | N/A |
| sll0944 | hypothetical protein | Hypothetical | N/A |
| sll0980 | unknown protein | Unknown | N/A |
| sll0981 | unknown protein | Unknown | N/A |
| sll0984 | hypothetical protein | Hypothetical | N/A |
| sll0986 | putative transposase [ISY120f(partial copy): 664387 - 664775] | Other categories | Transposon-related functions |
| sll0990 | glutathione-dependent formaldehyde dehydrogenase | Other categories | Other |
| sll0993 | potassium channel | Transport and binding proteins | N/A |
| sll0994 | hypothetical protein | Hypothetical | N/A |
| sll0995 | hypothetical protein | Hypothetical | N/A |
| sll1001 | ATP-binding protein of ABC transporter | Transport and binding proteins | N/A |
| sll1003 | two-component sensor histidine kinase | Regulatory functions | N/A |
| sll1006 | unknown protein | Unknown | N/A |
| sll1011 | hypothetical protein | Hypothetical | N/A |
| sll1020 | plobable glycosyltransferase | Other categories | Other |
| sll1022 | hypothetical protein | Hypothetical | N/A |
| sll1024 | hypothetical protein | Hypothetical | N/A |
| sll1025 | hypothetical protein | Hypothetical | N/A |
| sll1045 | mutator MutT protein | DNA replication, restriction, modification, recombination, and repair | N/A |
| sll1052 | hypothetical protein | Hypothetical | N/A |
| sll1054 | hypothetical protein | Hypothetical | N/A |
| sll1057 | thioredoxin M | Biosynthesis of cofactors, prosthetic groups, and carriers | Thioredoxin, glutaredoxin, and glutathione |
| sll1059 | adenylate kinase | Purines, pyrimidines, nucleosides, and nucleotides | Purine ribonucleotide biosynthesis |
| sll1061 | unknown protein | Unknown | N/A |
| sll1062 | unknown protein | Unknown | N/A |
| sll1063 | hypothetical protein | Hypothetical | N/A |
| sll1068 | unknown protein | Unknown | N/A |
| sll1078 | putative hydrogenase expression/formation protein HypA | Other categories | Hydrogenase |
| sll1079 | putative hydrogenase expression/formation protein HypB | Other categories | Hydrogenase |
| sll1082 | ABC transport system ATP-binding protein | Transport and binding proteins | N/A |
| sll1086 | unknown protein | Unknown | N/A |
| sll1094 | putative transposase: | Other categories | Transposon-related functions |
| sll1095 | hypothetical protein | Hypothetical | N/A |
| sll1102 | integral membrane protein (small) of a TRAP-type permease that mediates sodium-dependent glutamate transport GtrA | Transport and binding proteins | N/A |
| sll1103 | integral membrane protein (large) of a TRAP-type permease that mediates sodium-dependent glutamate transport GtrB | Transport and binding proteins | N/A |
| sll1104 | periplasmic substrate-binding protein of a TRAP-type permease that mediates sodium-dependent glutamate transport GtrC | Transport and binding proteins | N/A |
| sll1119 | hypothetical protein | Hypothetical | N/A |
| sll1123 | hypothetical protein | Hypothetical | N/A |
| sll1131 | unknown protein | Unknown | N/A |
| sll1132 | unknown protein | Unknown | N/A |
| sll1142 | hypothetical protein | Hypothetical | N/A |
| sll1147 | glutathione S-transferase | Biosynthesis of cofactors, prosthetic groups, and carriers | Thioredoxin, glutaredoxin, and glutathione |
| sll1150 | hypothetical protein | Hypothetical | N/A |
| sll1154 | putative antibiotic efflux protein | Other categories | Drug and analog sensitivity |
| sll1155 | hypothetical protein | Hypothetical | N/A |
| sll1156 | putative transposase [ISY120b: 1385747 - 1386548] | Other categories | Transposon-related functions |
| sll1157 | putative transposase [ISY120b: 1385747 - 1386548] | Other categories | Transposon-related functions |
| sll1158 | hypothetical protein | Hypothetical | N/A |
| sll1160 | hypothetical protein | Hypothetical | N/A |
| sll1162 | hypothetical protein | Hypothetical | N/A |
| sll1163 | unknown protein | Unknown | N/A |
| sll1164 | hypothetical protein | Hypothetical | N/A |
| sll1166 | hypothetical protein | Hypothetical | N/A |
| sll1167 | unknown protein | Unknown | N/A |
| sll1169 | hypothetical protein | Hypothetical | N/A |
| sll1170 | unknown protein | Unknown | N/A |
| sll1174 | unknown protein | Unknown | N/A |
| sll1182 | cytochrome b6f complex alternative iron-sulfur subunit (Rieske iron sulfur protein) | Photosynthesis and respiration | Cytochrome b6/f complex |
| sll1186 | hypothetical protein | Hypothetical | N/A |
| sll1187 | prolipoprotein diacylglyceryl transferase | Cell envelope | Membranes, lipoproteins, and porins |
| sll1189 | glycolate oxidase subunit GlcE | Energy metabolism | Glycolate pathway |
| sll1191 | hypothetical protein | Hypothetical | N/A |
| sll1192 | hypothetical protein | Hypothetical | N/A |
| sll1193 | hypothetical protein | Hypothetical | N/A |
| sll1200 | hypothetical protein | Hypothetical | N/A |
| sll1201 | hypothetical protein | Hypothetical | N/A |
| sll1203 | hypothetical protein | Hypothetical | N/A |
| sll1204 | similar to macrolide efflux protein | Transport and binding proteins | N/A |
| sll1205 | transcriptional regulator | Regulatory functions | N/A |
| sll1217 | unknown protein | Unknown | N/A |
| sll1219 | hypothetical protein | Hypothetical | N/A |
| sll1222 | hypothetical protein | Hypothetical | N/A |
| sll1225 | unknown protein | Unknown | N/A |
| sll1228 | two-component hybrid sensor and regulator | Regulatory functions | N/A |
| sll1232 | hypothetical protein | Hypothetical | N/A |
| sll1236 | unknown protein | Unknown | N/A |
| sll1237 | N(5)-glutamine methyltransferase | Biosynthesis of cofactors, prosthetic groups, and carriers | Cobalamin, heme, phycobilin and porphyrin |
| sll1240 | unknown protein | Unknown | N/A |
| sll1241 | unknown protein | Unknown | N/A |
| sll1245 | cytochrome cM | Photosynthesis and respiration | Soluble electron carriers |
| sll1247 | hypothetical protein | Hypothetical | N/A |
| sll1250 | hypothetical protein | Hypothetical | N/A |
| sll1251 | hypothetical protein | Hypothetical | N/A |
| sll1253 | similar to polyA polymerase | Other categories | Other |
| sll1254 | hypothetical protein | Hypothetical | N/A |
| sll1255 | putative transposase [ISY203c: 1728942 - 1730115] | Other categories | Transposon-related functions |
| sll1256 | putative transposase [ISY100p: 1725405 - 1726350] | Other categories | Transposon-related functions |
| sll1257 | putative transposase [ISY100p: 1725405 - 1726350] | Other categories | Transposon-related functions |
| sll1263 | cation efflux system protein | Transport and binding proteins | N/A |
| sll1267 | unknown protein | Unknown | N/A |
| sll1268 | unknown protein | Unknown | N/A |
| sll1277 | RecF protein | DNA replication, restriction, modification, recombination, and repair | N/A |
| sll1281 | photosystem II PsbZ protein | Hypothetical | N/A |
| sll1291 | two-component response regulator PatA subfamily | Regulatory functions | N/A |
| sll1292 | two-component response regulator CheY subfamily | Regulatory functions | N/A |
| sll1293 | unknown protein | Unknown | N/A |
| sll1297 | probable dioxygenase, Rieske iron-sulfur component | Other categories | Other |
| sll1318 | hypothetical protein | Hypothetical | N/A |
| sll1319 | hypothetical protein | Hypothetical | N/A |
| sll1321 | hypothetical protein | Photosynthesis and respiration | ATP synthase |
| sll1333 | unknown protein | Unknown | N/A |
| sll1340 | hypothetical protein | Hypothetical | N/A |
| sll1344 | unknown protein | Unknown | N/A |
| sll1348 | hypothetical protein | Hypothetical | N/A |
| sll1352 | unknown protein | Unknown | N/A |
| sll1353 | two-component sensor histidine kinase | Regulatory functions | N/A |
| sll1355 | hypothetical protein | Hypothetical | N/A |
| sll1359 | unknown protein | Unknown | N/A |
| sll1369 | putative peptidase | Other categories | Other |
| sll1372 | hypothetical protein | Hypothetical | N/A |
| sll1373 | unknown protein | Unknown | N/A |
| sll1381 | hypothetical protein | Hypothetical | N/A |
| sll1382 | ferredoxin, petF-like protein | Photosynthesis and respiration | Soluble electron carriers |
| sll1387 | serine/threonine protein phosphatase PppA | Regulatory functions | N/A |
| sll1388 | hypothetical protein | Hypothetical | N/A |
| sll1389 | hypothetical protein | Hypothetical | N/A |
| sll1392 | transcriptional regulator | Regulatory functions | N/A |
| sll1397 | putative transposase [ISY100a: 52234 - 53180] | Other categories | Transposon-related functions |
| sll1400 | hypothetical protein | Hypothetical | N/A |
| sll1407 | probable methyltransferase | Other categories | Other |
| sll1408 | transcriptional regulator | Regulatory functions | N/A |
| sll1409 | ferrichrome-iron receptor | Transport and binding proteins | N/A |
| sll1426 | unknown protein | Unknown | N/A |
| sll1428 | probable sodium-dependent transporter | Transport and binding proteins | N/A |
| sll1429 | unknown protein | Unknown | N/A |
| sll1432 | putative hydrogenase expression/formation protein HypB | Other categories | Hydrogenase |
| sll1436 | putative transposase [ISY100q: 1901359 - 1902304] | Other categories | Transposon-related functions |
| sll1437 | putative transposase [ISY100q: 1901359 - 1902304] | Other categories | Transposon-related functions |
| sll1442 | hypothetical protein | Hypothetical | N/A |
| sll1446 | hypothetical protein | Hypothetical | N/A |
| sll1447 | hypothetical protein | Hypothetical | N/A |
| sll1454 | ferredoxin-nitrate reductase | Amino acid biosynthesis | Glutamate family / Nitrogen assimilation |
| sll1455 | hypothetical protein | Hypothetical | N/A |
| sll1461 | hypothetical protein | Hypothetical | N/A |
| sll1462 | putative hydrogenase expression/formation protein HypE | Other categories | Hydrogenase |
| sll1466 | probable glycosyltransferase | Other categories | Other |
| sll1468 | beta-carotene hydroxylase | Biosynthesis of cofactors, prosthetic groups, and carriers | Carotenoid |
| sll1472 | unknown protein | Unknown | N/A |
| sll1474 | putative transposase [ISY203g: 3400332 - 3401505] | Other categories | Transposon-related functions |
| sll1476 | unknown protein | Unknown | N/A |
| sll1482 | ABC transporter permease protein | Transport and binding proteins | N/A |
| sll1485 | hypothetical protein | Hypothetical | N/A |
| sll1486 | hypothetical protein | Hypothetical | N/A |
| sll1488 | hypothetical protein | Hypothetical | N/A |
| sll1500 | hypothetical protein | Hypothetical | N/A |
| sll1501 | cobyrinic acid a,c-diamide synthase | Biosynthesis of cofactors, prosthetic groups, and carriers | Cobalamin, heme, phycobilin and porphyrin |
| sll1503 | unknown protein | Unknown | N/A |
| sll1504 | hypothetical protein | Hypothetical | N/A |
| sll1509 | hypothetical protein YCF20 | Hypothetical | N/A |
| sll1511 | unknown protein | Unknown | N/A |
| sll1512 | hypothetical protein | Hypothetical | N/A |
| sll1515 | glutamine synthetase inactivating factor IF17 | Unknown | N/A |
| sll1522 | CDP-diacylglycerol--glycerol-3-phosphate 3-phosphatidyltransferase | Fatty acid, phospholipid and sterol metabolism | N/A |
| sll1540 | dolichyl-phosphate-mannose synthase | Central intermediary metabolism | Polysaccharides and glycoproteins |
| sll1543 | hypothetical protein | Hypothetical | N/A |
| sll1547 | hypothetical protein | Hypothetical | N/A |
| sll1552 | unknown protein | Unknown | N/A |
| sll1560 | putative transposase [ISY203d: 1970882 - 1972055] | Other categories | Transposon-related functions |
| sll1562 | unknown protein | Unknown | N/A |
| sll1573 | hypothetical protein | Hypothetical | N/A |
| sll1574 | a part of spkA | Regulatory functions | N/A |
| sll1575 | a part of spkA | Regulatory functions | N/A |
| sll1584 | ferredoxin like protein | Photosynthesis and respiration | Soluble electron carriers |
| sll1592 | two-component response regulator NarL subfamily | Regulatory functions | N/A |
| sll1596 | circadian clock protein KaiB homolog | Other categories | Other |
| sll1598 | Mn transporter MntC | Transport and binding proteins | N/A |
| sll1599 | manganese transport system ATP-binding protein MntA | Transport and binding proteins | N/A |
| sll1600 | manganese transport system membrane protein MntB | Transport and binding proteins | N/A |
| sll1601 | hypothetical protein | Hypothetical | N/A |
| sll1611 | unknown protein | Unknown | N/A |
| sll1613 | unknown protein | Unknown | N/A |
| sll1618 | hypothetical protein | Hypothetical | N/A |
| sll1629 | bacterial cryptochrome | DNA replication, restriction, modification, recombination, and repair | N/A |
| sll1631 | putative cytidine and deoxycytidylate deaminase | Purines, pyrimidines, nucleosides, and nucleotides | Pyrimidine ribonucleotide biosynthesis |
| sll1632 | hypothetical protein | Hypothetical | N/A |
| sll1639 | urease accessory protein D | Central intermediary metabolism | Other |
| sll1642 | hypothetical protein | Hypothetical | N/A |
| sll1643 | hypothetical protein | Hypothetical | N/A |
| sll1647 | probable phosphinothricin N-acetyltransferase | Other categories | Other |
| sll1651 | hypothetical protein | Hypothetical | N/A |
| sll1652 | hypothetical protein | Hypothetical | N/A |
| sll1658 | hypothetical protein | Hypothetical | N/A |
| sll1659 | hypothetical protein | Hypothetical | N/A |
| sll1660 | hypothetical protein | Hypothetical | N/A |
| sll1666 | DnaJ-like protein | Cellular processes | Chaperones |
| sll1670 | heat-inducible transcription repressor HrcA homolog | Regulatory functions | N/A |
| sll1671 | hypothetical protein | Hypothetical | N/A |
| sll1678 | similar to spore maturation protein A | Other categories | Adaptations and atypical conditions |
| sll1680 | hypothetical protein | Hypothetical | N/A |
| sll1681 | unknown protein | Unknown | N/A |
| sll1683 | lysine decarboxylase | Energy metabolism | Amino acids and amines |
| sll1685 | protein involved in light-induced Na+-dependent proton extrusion | Biosynthesis of cofactors, prosthetic groups, and carriers | Cobalamin, heme, phycobilin and porphyrin |
| sll1686 | hypothetical protein | Hypothetical | N/A |
| sll1691 | hypothetical protein | Hypothetical | N/A |
| sll1692 | hypothetical protein | Hypothetical | N/A |
| sll1695 | pilin polypeptide PilA2 | Cellular processes | Chemotaxis |
| sll1698 | hypothetical protein | Hypothetical | N/A |
| sll1702 | hypothetical protein YCF51 | Hypothetical | N/A |
| sll1704 | probable short chain dehydrogenase | Other categories | Other |
| sll1708 | two-component response regulator NarL subfamily | Regulatory functions | N/A |
| sll1710 | putative transposase [ISY523b: 1275354 - 1276224] | Other categories | Transposon-related functions |
| sll1714 | unknown protein | Unknown | N/A |
| sll1715 | hypothetical protein | Hypothetical | N/A |
| sll1716 | putative transposase [ISY523a: 967549 - 968419] | Other categories | Transposon-related functions |
| sll1717 | unknown protein | Unknown | N/A |
| sll1722 | hypothetical protein | Hypothetical | N/A |
| sll1724 | probable glycosyltransferase | Cell envelope | Surface polysaccharides, lipopolysaccharides and antigens |
| sll1726 | hypothetical protein | Hypothetical | N/A |
| sll1730 | unknown protein | Unknown | N/A |
| sll1736 | hypothetical protein | Hypothetical | N/A |
| sll1737 | hypothetical protein YCF60 | Hypothetical | N/A |
| sll1738 | hypothetical protein | Hypothetical | N/A |
| sll1749 | hypothetical protein | Hypothetical | N/A |
| sll1751 | hypothetical protein | Hypothetical | N/A |
| sll1755 | unknown protein | Unknown | N/A |
| sll1758 | MrsA protein homolog | Other categories | Other |
| sll1761 | unknown protein | Unknown | N/A |
| sll1763 | unknown protein | Unknown | N/A |
| sll1764 | unknown protein | Unknown | N/A |
| sll1765 | unknown protein | Unknown | N/A |
| sll1773 | hypothetical protein | Hypothetical | N/A |
| sll1780 | putative transposase [ISY203b: 1200306 - 1201479] | Other categories | Transposon-related functions |
| sll1783 | hypothetical protein | Hypothetical | N/A |
| sll1791 | putative transposase [ISY802a: 852462 - 853369] | Other categories | Transposon-related functions |
| sll1792 | putative transposase [ISY802a: 852462 - 853369] | Other categories | Transposon-related functions |
| sll1796 | cytochrome c553 | Photosynthesis and respiration | Soluble electron carriers |
| sll1797 | hypothetical protein YCF21 | Hypothetical | N/A |
| sll1824 | 50S ribosomal protein L25 | Translation | Ribosomal proteins |
| sll1831 | glycolate oxidase subunit, (Fe-S)protein | Energy metabolism | Glycolate pathway |
| sll1832 | hypothetical protein | Hypothetical | N/A |
| sll1834 | hypothetical protein | Hypothetical | N/A |
| sll1845 | hypothetical protein | Hypothetical | N/A |
| sll1849 | probable dioxygenase Rieske iron-sulfur component | Other categories | Other |
| sll1851 | unknown protein | Unknown | N/A |
| sll1853 | unknown protein | Unknown | N/A |
| sll1860 | putative transposase [ISY523d: 2226601 - 2227471] | Other categories | Transposon-related functions |
| sll1861 | putative transposase [ISY523o(partial copy): 2225804 - 2226597] | Other categories | Transposon-related functions |
| sll1865 | peptide chain release factor 2 | Translation | Protein modification and translation factors |
| sll1866 | hypothetical protein | Hypothetical | N/A |
| sll1867 | photosystem II D1 protein | Photosynthesis and respiration | Photosystem II |
| sll1869 | probable dioxygenase, Rieske iron-sulfur component | Other categories | Other |
| sll1874 | hypothetical protein | Hypothetical | N/A |
| sll1875 | heme oxygenase | Biosynthesis of cofactors, prosthetic groups, and carriers | Cobalamin, heme, phycobilin and porphyrin |
| sll1880 | hypothetical protein | Hypothetical | N/A |
| sll1882 | unknown protein | Unknown | N/A |
| sll1886 | hypothetical protein | Hypothetical | N/A |
| sll1890 | cobalt-chelatase subunit CobN-like protein | Biosynthesis of cofactors, prosthetic groups, and carriers | Cobalamin, heme, phycobilin and porphyrin |
| sll1892 | unknown protein | Unknown | N/A |
| sll1897 | hypothetical protein | Hypothetical | N/A |
| sll1899 | cytochrome c oxidase folding protein | Photosynthesis and respiration | Respiratory terminal oxidases |
| sll1900 | acetyltransferase | Other categories | Other |
| sll1912 | hypothetical protein | Hypothetical | N/A |
| sll1916 | hypothetical protein | Hypothetical | N/A |
| sll1917 | coproporphyrinogen III oxidase, anaerobic (oxygen-independent) | Biosynthesis of cofactors, prosthetic groups, and carriers | Cobalamin, heme, phycobilin and porphyrin |
| sll1921 | hypothetical protein | Hypothetical | N/A |
| sll1924 | cAMP receptor protein sycrp1 homolog | Regulatory functions | N/A |
| sll1926 | hypothetical protein | Hypothetical | N/A |
| sll1930 | putative transposase [ISY100k: 605515 - 606460] | Other categories | Transposon-related functions |
| sll1933 | DnaJ protein, heat shock protein 40, molecular chaperone | Cellular processes | Chaperones |
| sll1937 | ferric uptake regulation protein | Regulatory functions | N/A |
| sll1938 | hypothetical protein | Hypothetical | N/A |
| sll1939 | unknown protein | Unknown | N/A |
| sll1954 | unknown protein | Unknown | N/A |
| sll1956 | hypothetical protein | Hypothetical | N/A |
| sll1957 | transcriptional regulator | Regulatory functions | N/A |
| sll1959 | probable inositol monophosphatase | Other categories | Other |
| sll1960 | hypothetical protein | Hypothetical | N/A |
| sll1965 | hypothetical protein | Hypothetical | N/A |
| sll1968 | photomixotrophic growth related protein, PmgA | Other categories | Other |
| sll1969 | hypothetical protein | Hypothetical | N/A |
| sll1971 | probable hexosyltransferase | Other categories | Other |
| sll1973 | hypothetical protein | Hypothetical | N/A |
| sll1982 | putative transposase [ISY352c: 1553414 - 1553903, join 1554854 - 1555790] | Other categories | Transposon-related functions |
| sll1983 | putative transposase [ISY100n: 1553903 - 1554848] | Other categories | Transposon-related functions |
| sll1984 | putative transposase [ISY100n: 1553903 - 1554848] | Other categories | Transposon-related functions |
| sll1985 | putative transposase [ISY352c: 1553414 - 1553903, join 1554854 - 1555790] | Other categories | Transposon-related functions |
| sll1995 | hypothetical protein | Hypothetical | N/A |
| sll1997 | putative transposase [ISY203h: 1623060 - 1623693, join 1624643 - 1625182] | Other categories | Transposon-related functions |
| sll1998 | putative transposase [ISY100d: 1623697 - 1624643] | Other categories | Transposon-related functions |
| sll1999 | putative transposase [ISY203h: 1623060 - 1623693, join 1624643 - 1625182] | Other categories | Transposon-related functions |
| sll2006 | hypothetical protein | Hypothetical | N/A |
| sll2007 | hypothetical protein | Hypothetical | N/A |
| sll2012 | group2 RNA polymerase sigma factor SigD | Transcription | RNA synthesis, modification, and DNA transcription |
| sll2013 | hypothetical protein | Hypothetical | N/A |
| sll2015 | hypothetical protein | Hypothetical | N/A |
| sll5002 | unknown protein | Unknown | N/A |
| sll5003 | hypothetical protein | Hypothetical | N/A |
| sll5004 | hypothetical protein | Hypothetical | N/A |
| sll5006 | unknown protein | Unknown | N/A |
| sll5014 | similar to maturase: | Other categories | Transposon-related functions |
| sll5026 | hypothetical protein | Hypothetical | N/A |
| sll5028 | hypothetical protein | Hypothetical | N/A |
| sll5030 | hypothetical protein | Hypothetical | N/A |
| sll5032 | hypothetical protein | Hypothetical | N/A |
| sll5033 | hypothetical protein | Hypothetical | N/A |
| sll5034 | hypothetical protein | Hypothetical | N/A |
| sll5035 | transcriptional regulatory protein ArsR family | Regulatory functions | N/A |
| sll5036 | sulfide-quinone reductase | Other categories | Other |
| sll5041 | putative transposase [ISY523u: 38789 - 39659] | Other categories | Transposon-related functions |
| sll5042 | probable sulfotransferase | Other categories | Other |
| sll5043 | probable glycosyltransferase | Other categories | Other |
| sll5044 | unknown protein | Unknown | N/A |
| sll5046 | unknown protein | Unknown | N/A |
| sll5047 | unknown protein | Unknown | N/A |
| sll5048 | probable glycosyltransferase | Other categories | Other |
| sll5049 | similar to polysaccharide biosynthesis protein | Other categories | Other |
| sll5050 | probable glycosyltransferase | Other categories | Other |
| sll5052 | similar to exopolysaccharide export protein | Other categories | Other |
| sll5057 | probable glycosyltransferase | Other categories | Other |
| sll5059 | two-component response regulator | Regulatory functions | N/A |
| sll5060 | two-component hybrid sensor & regulator | Regulatory functions | N/A |
| sll5061 | hypothetical protein | Hypothetical | N/A |
| sll5062 | unknown protein | Unknown | N/A |
| sll5063 | unknown protein | Unknown | N/A |
| sll5066 | probable plasmid partitioning protein, ParA family | Other categories | Other |
| sll5067 | hypothetical protein | Hypothetical | N/A |
| sll5069 | unknown protein | Unknown | N/A |
| sll5072 | hypothetical protein | Hypothetical | N/A |
| sll5075 | hypothetical protein | Hypothetical | N/A |
| sll5076 | hypothetical protein | Hypothetical | N/A |
| sll5079 | probable short chain dehydrogenase | Other categories | Other |
| sll5081 | hypothetical protein | Hypothetical | N/A |
| sll5083 | unknown protein | Unknown | N/A |
| sll5084 | probable plasmid partitioning protein, ParB | Other categories | Other |
| sll5086 | transcriptional regulatory protein GntR family | Regulatory functions | N/A |
| sll5089 | unknown protein | Unknown | N/A |
| sll5090 | unknown protein | Unknown | N/A |
| sll5094 | plasmid stabilization protein | Other categories | Other |
| sll5097 | hypothetical protein | Hypothetical | N/A |
| sll5104 | arsenate reductase | Cellular processes | Detoxification |
| sll5107 | cytidine deaminase | Purines, pyrimidines, nucleosides, and nucleotides | Interconversions and salvage of nucleosides and nucleotides |
| sll5109 | unknown protein | Unknown | N/A |
| sll5122 | SOS mutagenesis and repair, UmuC protein homolog | DNA replication, restriction, modification, recombination, and repair | N/A |
| sll5123 | SOS mutagenesis and repair, UmuD protein homolog | DNA replication, restriction, modification, recombination, and repair | N/A |
| sll5128 | unknown protein | Unknown | N/A |
| sll5130 | hypothetical protein | Hypothetical | N/A |
| sll5131 | putative transposase [ISY203j: 117275 - 118448] | Other categories | Transposon-related functions |
| sll5132 | hypothetical protein | Hypothetical | N/A |
| sll6010 | unknown protein | Unknown | N/A |
| sll6017 | hypothetical protein | Hypothetical | N/A |
| sll6036 | chromosome partitioning protein, ParA family | Other categories | Other |
| sll6052 | hypothetical protein | Hypothetical | N/A |
| sll6053 | hypothetical protein | Hypothetical | N/A |
| sll6054 | hypothetical protein | Hypothetical | N/A |
| sll6055 | hypothetical protein | Hypothetical | N/A |
| sll6059 | putative integrase/recombinase: | Other categories | Transposon-related functions |
| sll6060 | probable acetyltransferase | Other categories | Other |
| sll6069 | unknown protein | Unknown | N/A |
| sll6076 | hypothetical protein | Hypothetical | N/A |
| sll6093 | chromosome partitioning protein, ParA family | Other categories | Other |
| sll6098 | hypothetical protein | Hypothetical | N/A |
| sll6109 | putative integrase/recombinase: | Other categories | Transposon-related functions |
| sll7001 | putative transposase [ISY391e(partial copy): 166 - 1298] | Other categories | Transposon-related functions |
| sll7002 | putative transposase [ISY391e(partial copy): 166 - 1298] | Other categories | Transposon-related functions |
| sll7003 | plasmid stability protein | Other categories | Other |
| sll7006 | unknown protein | Unknown | N/A |
| sll7009 | unknown protein | Unknown | N/A |
| sll7027 | unknown protein | Unknown | N/A |
| sll7028 | hypothetical protein | Hypothetical | N/A |
| sll7029 | hypothetical protein | Hypothetical | N/A |
| sll7030 | hypothetical protein | Hypothetical | N/A |
| sll7031 | hypothetical protein | Hypothetical | N/A |
| sll7033 | hypothetical protein | Hypothetical | N/A |
| sll7034 | hypothetical protein | Hypothetical | N/A |
| sll7043 | unknown protein | Unknown | N/A |
| sll7044 | plasmid partition protein ParA homolog | Other categories | Other |
| sll7047 | hypothetical protein | Hypothetical | N/A |
| sll7050 | unknown protein | Unknown | N/A |
| sll7055 | unknown protein | Unknown | N/A |
| sll7056 | unknown protein | Unknown | N/A |
| sll7062 | unknown protein | Unknown | N/A |
| sll7063 | unknown protein | Unknown | N/A |
| sll7064 | unknown protein | Unknown | N/A |
| sll7065 | unknown protein | Unknown | N/A |
| sll7066 | unknown protein | Unknown | N/A |
| sll7067 | unknown protein | Unknown | N/A |
| sll7069 | hypothetical protein | Hypothetical | N/A |
| sll7070 | unknown protein | Unknown | N/A |
| sll7075 | unknown protein | Unknown | N/A |
| sll7077 | hypothetical protein | Hypothetical | N/A |
| sll7078 | unknown protein | Unknown | N/A |
| sll7085 | unknown protein | Unknown | N/A |
| sll7086 | unknown protein | Unknown | N/A |
| sll7089 | unknown protein | Unknown | N/A |
| sll7090 | unknown protein | Unknown | N/A |
| sll7103 | exodeoxyribonuclease V, alpha chain | DNA replication, restriction, modification, recombination, and repair | N/A |
| sll7106 | exodeoxyribonuclease V, alpha chain | DNA replication, restriction, modification, recombination, and repair | N/A |
| sll8001 | hypothetical protein | Hypothetical | N/A |
| sll8002 | hypothetical protein | Hypothetical | N/A |
| sll8006 | type I restriction-modification system, S subunit | DNA replication, restriction, modification, recombination, and repair | N/A |
| sll8007 | unknown protein | Unknown | N/A |
| sll8009 | type I restriction-modification system, M subunit | DNA replication, restriction, modification, recombination, and repair | N/A |
| sll8011 | unknown protein | Unknown | N/A |
| sll8012 | unknown protein | Unknown | N/A |
| sll8017 | unknown protein | Unknown | N/A |
| sll8018 | hypothetical protein | Hypothetical | N/A |
| sll8019 | hypothetical protein | Hypothetical | N/A |
| sll8020 | hypothetical protein | Hypothetical | N/A |
| sll8025 | hypothetical protein | Hypothetical | N/A |
| sll8027 | hypothetical protein | Hypothetical | N/A |
| sll8031 | NADH dehydrogenase subunit NdhK | Photosynthesis and respiration | NADH dehydrogenase |
| sll8032 | hypothetical protein | Hypothetical | N/A |
| sll8033 | unknown protein | Unknown | N/A |
| sll8034 | 2-nitropropane dioxygenase | Other categories | Other |
| sll8035 | hypothetical protein | Hypothetical | N/A |
| sll8040 | unknown protein | Unknown | N/A |
| sll8042 | putative transposase [ISY100y: 38542 - 39487] | Other categories | Transposon-related functions |
| sll8043 | putative transposase [ISY100y: 38542 - 39487] | Other categories | Transposon-related functions |
| sll8048 | hypothetical protein | Hypothetical | N/A |
| sll8049 | type I site-specific deoxyribonuclease chain R | DNA replication, restriction, modification, recombination, and repair | N/A |
| sll9006 | unknown protein [ORF-E] | Unknown | N/A |
| slr0008 | carboxyl-terminal processing protease | Translation | Degradation of proteins, peptides, and glycopeptides |
| slr0011 | possible Rubisco chaperonin | Hypothetical | N/A |
| slr0014 | Mg2+ transport ATPase | Transport and binding proteins | N/A |
| slr0019 | unknown protein | Unknown | N/A |
| slr0020 | DNA recombinase | DNA replication, restriction, modification, recombination, and repair | N/A |
| slr0022 | hypothetical protein | Hypothetical | N/A |
| slr0033 | glutamyl-tRNA(Gln) amidotransferase subunit C | Translation | Aminoacyl tRNA synthetases and tRNA modification |
| slr0036 | aspartate aminotransferase | Amino acid biosynthesis | Aspartate family |
| slr0041 | bicarbonate transport system permease protein | Transport and binding proteins | N/A |
| slr0043 | bicarbonate transport system ATP-binding protein | Transport and binding proteins | N/A |
| slr0044 | bicarbonate transport system ATP-binding protein | Transport and binding proteins | N/A |
| slr0053 | hypothetical protein | Hypothetical | N/A |
| slr0054 | diacylglycerol kinase | Fatty acid, phospholipid and sterol metabolism | N/A |
| slr0056 | chlorophyll a synthase | Biosynthesis of cofactors, prosthetic groups, and carriers | Cobalamin, heme, phycobilin and porphyrin |
| slr0059 | unknown protein | Unknown | N/A |
| slr0060 | unknown protein | Unknown | N/A |
| slr0061 | unknown protein | Unknown | N/A |
| slr0066 | riboflavin biosynthesis protein RibD | Biosynthesis of cofactors, prosthetic groups, and carriers | Riboflavin |
| slr0069 | unknown protein | Unknown | N/A |
| slr0078 | putative 6-pyruvoyl tetrahydrobiopterin synthase | Other categories | Other |
| slr0081 | two-component response regulator OmpR subfamily | Regulatory functions | N/A |
| slr0084 | amidotransferase HisH | Amino acid biosynthesis | Aromatic amino acid family |
| slr0090 | probable 4-hydroxyphenylpyruvate dioxygenase | Energy metabolism | Amino acids and amines |
| slr0091 | aldehyde dehydrogenase | Energy metabolism | Pyruvate and acetyl-CoA metabolism |
| slr0092 | hypothetical protein | Hypothetical | N/A |
| slr0093 | DnaJ protein, heat shock protein 40, molecular chaperone | Cellular processes | Chaperones |
| slr0095 | O-methyltransferase | Other categories | Other |
| slr0099 | putative transposase [ISY352f(partial copy): 2968019 - 2969383] | Other categories | Transposon-related functions |
| slr0103 | unknown protein | Unknown | N/A |
| slr0105 | hypothetical protein | Hypothetical | N/A |
| slr0108 | unknown protein | Unknown | N/A |
| slr0109 | unknown protein | Unknown | N/A |
| slr0111 | unknown protein | Unknown | N/A |
| slr0142 | hypothetical protein | Hypothetical | N/A |
| slr0150 | ferredoxin, petF-like protein | Photosynthesis and respiration | Soluble electron carriers |
| slr0157 | unknown protein | Unknown | N/A |
| slr0166 | putative transposase [ISY523n: 2210271 - 2211142] | Other categories | Transposon-related functions |
| slr0168 | unknown protein | Unknown | N/A |
| slr0180 | putative transposase [ISY203f: 2326926 - 2328099] | Other categories | Transposon-related functions |
| slr0181 | hypothetical protein | Hypothetical | N/A |
| slr0192 | hypothetical protein | Hypothetical | N/A |
| slr0195 | hypothetical protein | Hypothetical | N/A |
| slr0196 | unknown protein | Unknown | N/A |
| slr0204 | hypothetical protein YCF83 | Hypothetical | N/A |
| slr0210 | two-component sensor histidine kinase | Regulatory functions | N/A |
| slr0211 | hypothetical protein | Hypothetical | N/A |
| slr0214 | cytosine-specific methyltransferase(5'-CGATCG-3') | DNA replication, restriction, modification, recombination, and repair | N/A |
| slr0216 | bifunctional cobalamin biosynthesis protein CobP | Biosynthesis of cofactors, prosthetic groups, and carriers | Cobalamin, heme, phycobilin and porphyrin |
| slr0217 | hypothetical protein | Hypothetical | N/A |
| slr0224 | similar to sterol C5-desaturase | Fatty acid, phospholipid and sterol metabolism | N/A |
| slr0230 | putative transposase [ISY100f: 2534034 - 2534980] | Other categories | Transposon-related functions |
| slr0231 | probable DNA-3-methyladenine glycosylase | Other categories | Other |
| slr0233 | thioredoxin M | Biosynthesis of cofactors, prosthetic groups, and carriers | Thioredoxin, glutaredoxin, and glutathione |
| slr0240 | transcriptional regulator | Regulatory functions | N/A |
| slr0241 | hypothetical protein | Hypothetical | N/A |
| slr0243 | hypothetical protein | Hypothetical | N/A |
| slr0245 | histone deacetylase family protein | Other categories | Other |
| slr0249 | hypothetical protein | Hypothetical | N/A |
| slr0252 | probable precorrin-6x reductase | Biosynthesis of cofactors, prosthetic groups, and carriers | Cobalamin, heme, phycobilin and porphyrin |
| slr0262 | unknown protein | Unknown | N/A |
| slr0263 | hypothetical protein | Hypothetical | N/A |
| slr0264 | hypothetical protein | Hypothetical | N/A |
| slr0265 | putative transposase [ISY523c: 1513158 - 1514023] | Other categories | Transposon-related functions |
| slr0267 | hypothetical protein | Hypothetical | N/A |
| slr0269 | hypothetical protein | Hypothetical | N/A |
| slr0270 | hypothetical protein | Hypothetical | N/A |
| slr0271 | unknown protein | Unknown | N/A |
| slr0272 | unknown protein | Unknown | N/A |
| slr0273 | unknown protein | Unknown | N/A |
| slr0284 | hypothetical protein | Hypothetical | N/A |
| slr0285 | hypothetical protein | Hypothetical | N/A |
| slr0286 | protein involved in functional assembly of photosystem II | Unknown | N/A |
| slr0287 | hypothetical protein | Hypothetical | N/A |
| slr0291 | hypothetical protein | Hypothetical | N/A |
| slr0292 | hypothetical protein | Hypothetical | N/A |
| slr0294 | unknown protein | Unknown | N/A |
| slr0299 | hypothetical protein | Hypothetical | N/A |
| slr0300 | hypothetical protein | Hypothetical | N/A |
| slr0302 | unknown protein | Unknown | N/A |
| slr0304 | hypothetical protein | Hypothetical | N/A |
| slr0305 | hypothetical protein | Hypothetical | N/A |
| slr0306 | unknown protein | Unknown | N/A |
| slr0309 | probable methyltransferase | Other categories | Other |
| slr0312 | two-component response regulator NarL subfamily | Regulatory functions | N/A |
| slr0313 | hypothetical protein | Hypothetical | N/A |
| slr0316 | hypothetical protein | Hypothetical | N/A |
| slr0318 | unknown protein | Unknown | N/A |
| slr0319 | beta-lactamase | Other categories | Drug and analog sensitivity |
| slr0320 | hypothetical protein | Hypothetical | N/A |
| slr0325 | hypothetical protein | Hypothetical | N/A |
| slr0326 | hypothetical protein | Hypothetical | N/A |
| slr0327 | iron(III) ABC transporter, permease protein | Transport and binding proteins | N/A |
| slr0333 | unknown protein | Unknown | N/A |
| slr0334 | unknown protein | Unknown | N/A |
| slr0337 | hypothetical protein | Hypothetical | N/A |
| slr0341 | unknown protein | Unknown | N/A |
| slr0344 | probable glycosyltransferase | Cell envelope | Surface polysaccharides, lipopolysaccharides and antigens |
| slr0345 | unknown protein | Unknown | N/A |
| slr0346 | ribonuclease III | Transcription | Degradation of RNA |
| slr0347 | probable permease protein of ABC transporter | Transport and binding proteins | N/A |
| slr0350 | putative transposase [ISY523e: 2441031 - 2441901] | Other categories | Transposon-related functions |
| slr0352 | putative transposase [ISY100e: 2443927 - 2444873] | Other categories | Transposon-related functions |
| slr0353 | unknown protein | Unknown | N/A |
| slr0358 | unknown protein | Unknown | N/A |
| slr0360 | hypothetical protein | Hypothetical | N/A |
| slr0361 | probable ribosomal large subunit pseudouridine synthase B | Translation | Ribosomal proteins |
| slr0363 | hypothetical protein | Hypothetical | N/A |
| slr0364 | hypothetical protein | Hypothetical | N/A |
| slr0366 | unknown protein | Unknown | N/A |
| slr0368 | unknown protein | Unknown | N/A |
| slr0373 | hypothetical protein | Hypothetical | N/A |
| slr0376 | hypothetical protein | Hypothetical | N/A |
| slr0383 | hypothetical protein | Hypothetical | N/A |
| slr0388 | hypothetical protein | Hypothetical | N/A |
| slr0392 | unknown protein | Unknown | N/A |
| slr0393 | unknown protein | Unknown | N/A |
| slr0395 | nitrate assimilation transcriptional activator, LysR family protein | Amino acid biosynthesis | Glutamate family / Nitrogen assimilation |
| slr0407 | hypothetical protein | Hypothetical | N/A |
| slr0408 | unknown protein | Unknown | N/A |
| slr0416 | unknown protein | Unknown | N/A |
| slr0418 | putative transcripton factor DevT homolog | Regulatory functions | N/A |
| slr0420 | hypothetical protein | Hypothetical | N/A |
| slr0421 | unknown protein | Unknown | N/A |
| slr0438 | hypothetical protein | Hypothetical | N/A |
| slr0443 | hypothetical protein | Hypothetical | N/A |
| slr0445 | hypothetical protein | Hypothetical | N/A |
| slr0446 | DNA polymerase III delta' subunit | DNA replication, restriction, modification, recombination, and repair | N/A |
| slr0448 | DNA repair protein RadA | DNA replication, restriction, modification, recombination, and repair | N/A |
| slr0449 | probable transcriptional regulator | Regulatory functions | N/A |
| slr0456 | unknown protein | Unknown | N/A |
| slr0458 | unknown protein | Unknown | N/A |
| slr0459 | hypothetical protein | Hypothetical | N/A |
| slr0460 | putative transposase [ISY352g: 3511668 - 3512290, join 3513238 - 3514051] | Other categories | Transposon-related functions |
| slr0462 | putative transposase [ISY352g: 3511668 - 3512290, join 3513238 - 3514051] | Other categories | Transposon-related functions |
| slr0468 | unknown protein | Unknown | N/A |
| slr0473 | cyanobacterial phytochrome 1, two-component sensor histidine kinase | Regulatory functions | N/A |
| slr0474 | two-component response regulator CheY subfamily, regulator for phytochrome 1 (Cph1) | Regulatory functions | N/A |
| slr0479 | hypothetical protein | Hypothetical | N/A |
| slr0482 | unknown protein | Unknown | N/A |
| slr0487 | hypothetical protein | Hypothetical | N/A |
| slr0488 | virulence factor MviN homolog. | Cellular processes | Transformation |
| slr0489 | unknown protein | Unknown | N/A |
| slr0491 | hypothetical protein | Hypothetical | N/A |
| slr0492 | O-succinylbenzoic acid-CoA ligase | Biosynthesis of cofactors, prosthetic groups, and carriers | Menaquinone and ubiquinone |
| slr0493 | similar to mannose-1-phosphate guanylyltransferase | Energy metabolism | Sugars |
| slr0495 | HetI protein homolog | Cell envelope | Membranes, lipoproteins, and porins |
| slr0496 | unknown protein | Unknown | N/A |
| slr0498 | unknown protein | Unknown | N/A |
| slr0505 | hypothetical protein | Hypothetical | N/A |
| slr0509 | hypothetical protein | Hypothetical | N/A |
| slr0511 | putative transposase [ISY523g: 2921593 - 2922457] | Other categories | Transposon-related functions |
| slr0514 | unknown protein | Unknown | N/A |
| slr0517 | hypothetical protein | Hypothetical | N/A |
| slr0518 | similar to alpha-L-arabinofuranosidase B | Central intermediary metabolism | Polysaccharides and glycoproteins |
| slr0522 | unknown protein | Unknown | N/A |
| slr0526 | 3-methyl-2-oxobutanoate hydroxymethyltransferase | Biosynthesis of cofactors, prosthetic groups, and carriers | Pantothenate |
| slr0527 | transcription regulator ExsB homolog | Regulatory functions | N/A |
| slr0530 | glucosylglycerol transport system permease protein | Transport and binding proteins | N/A |
| slr0531 | glucosylglycerol transport system permease protein | Transport and binding proteins | N/A |
| slr0541 | probable amidotransferase | Other categories | Other |
| slr0544 | ATP-binding protein of ABC transporter | Transport and binding proteins | N/A |
| slr0553 | hypothetical protein | Hypothetical | N/A |
| slr0572 | unknown protein | Unknown | N/A |
| slr0573 | unknown protein | Unknown | N/A |
| slr0574 | cytochrome P450 | Fatty acid, phospholipid and sterol metabolism | N/A |
| slr0579 | unknown protein | Unknown | N/A |
| slr0581 | unknown protein | Unknown | N/A |
| slr0582 | unknown protein | Unknown | N/A |
| slr0587 | unknown protein | Unknown | N/A |
| slr0588 | hypothetical protein | Hypothetical | N/A |
| slr0593 | cAMP binding membrane protein | Unknown | N/A |
| slr0596 | hypothetical protein | Hypothetical | N/A |
| slr0598 | hypothetical protein | Hypothetical | N/A |
| slr0601 | unknown protein | Unknown | N/A |
| slr0602 | unknown protein | Unknown | N/A |
| slr0607 | hypothetical protein | Hypothetical | N/A |
| slr0608 | histidine biosynthesis bifunctional protein HisIE | Amino acid biosynthesis | Aromatic amino acid family |
| slr0609 | hypothetical protein | Hypothetical | N/A |
| slr0610 | hypothetical protein | Hypothetical | N/A |
| slr0612 | probable pseudouridine synthase | Translation | Aminoacyl tRNA synthetases and tRNA modification |
| slr0613 | hypothetical protein | Hypothetical | N/A |
| slr0616 | unknown protein | Unknown | N/A |
| slr0625 | hypothetical protein | Hypothetical | N/A |
| slr0630 | hypothetical protein | Hypothetical | N/A |
| slr0634 | unknown protein | Unknown | N/A |
| slr0636 | probable cobalamin [5'-phosphate] synthase | Biosynthesis of cofactors, prosthetic groups, and carriers | Cobalamin, heme, phycobilin and porphyrin |
| slr0639 | mechanosensitive ion channel homolog | Transport and binding proteins | N/A |
| slr0640 | two-component sensor histidine kinase | Regulatory functions | N/A |
| slr0642 | hypothetical protein | Hypothetical | N/A |
| slr0644 | nitrogen regulation protein NifR3 homolog | Amino acid biosynthesis | Glutamate family / Nitrogen assimilation |
| slr0646 | probable D-alanyl-D-alanine carboxypeptidase | Cell envelope | Murein sacculus and peptidoglycan |
| slr0651 | hypothetical protein | Hypothetical | N/A |
| slr0655 | hypothetical protein | Hypothetical | N/A |
| slr0656 | hypothetical protein | Hypothetical | N/A |
| slr0664 | hypothetical protein | Hypothetical | N/A |
| slr0666 | unknown protein | Unknown | N/A |
| slr0675 | unknown protein | Unknown | N/A |
| slr0679 | sun protein | Other categories | Other |
| slr0686 | hypothetical protein | Hypothetical | N/A |
| slr0687 | probable two-component response regulator | Regulatory functions | N/A |
| slr0688 | hypothetical protein | Hypothetical | N/A |
| slr0698 | hypothetical protein | Hypothetical | N/A |
| slr0699 | unknown protein | Unknown | N/A |
| slr0700 | probable amino acid permease | Other categories | Other |
| slr0701 | transcriptional regulator | Regulatory functions | N/A |
| slr0703 | putative transposase [ISY523i: 3096320 - 3097190] | Other categories | Transposon-related functions |
| slr0704 | putative transposase [ISY100g: 3097363 - 3098309] | Other categories | Transposon-related functions |
| slr0712 | hypothetical protein | Hypothetical | N/A |
| slr0722 | hypothetical protein | Hypothetical | N/A |
| slr0724 | HtaR suppressor protein homolog | Regulatory functions | N/A |
| slr0725 | hypothetical protein | Hypothetical | N/A |
| slr0728 | hypothetical protein | Hypothetical | N/A |
| slr0730 | hypothetical protein | Hypothetical | N/A |
| slr0732 | hypothetical protein | Hypothetical | N/A |
| slr0733 | integrase-recombinase protein | DNA replication, restriction, modification, recombination, and repair | N/A |
| slr0734 | hypothetical protein | Hypothetical | N/A |
| slr0740 | hypothetical protein | Hypothetical | N/A |
| slr0742 | hypothetical protein | Hypothetical | N/A |
| slr0747 | glucosylglycerol transport system ATP-binding protein | Transport and binding proteins | N/A |
| slr0751 | hypothetical protein | Hypothetical | N/A |
| slr0753 | probable transport protein | Transport and binding proteins | N/A |
| slr0755 | hypothetical protein | Hypothetical | N/A |
| slr0770 | hypothetical protein | Hypothetical | N/A |
| slr0771 | hypothetical protein | Hypothetical | N/A |
| slr0780 | hypothetical protein | Hypothetical | N/A |
| slr0784 | hypothetical protein | Hypothetical | N/A |
| slr0787 | hypothetical protein | Hypothetical | N/A |
| slr0788 | similar to pre-B cell enhancing factor | Other categories | Other |
| slr0789 | hypothetical protein | Hypothetical | N/A |
| slr0790 | similar to ultraviolet light resistance protein B | DNA replication, restriction, modification, recombination, and repair | N/A |
| slr0793 | cation efflux system protein involved in nickel and cobalt tolerance | Unknown | N/A |
| slr0795 | cation efflux system protein involved in nickel and cobalt tolerance | Hypothetical | N/A |
| slr0796 | nickel permease involved in nickel and cobalt tolerance | Transport and binding proteins | N/A |
| slr0797 | cobalt-transporting P-type ATPase (cobalt efflux pump) involved in cobalt tolerance | Transport and binding proteins | N/A |
| slr0799 | putative transposase [ISY802c: 3066278 - 3067184] | Other categories | Transposon-related functions |
| slr0800 | putative transposase [ISY802c: 3066278 - 3067184] | Other categories | Transposon-related functions |
| slr0801 | hypothetical protein | Hypothetical | N/A |
| slr0807 | probable o-sialoglycoprotein endopeptidase | Translation | Degradation of proteins, peptides, and glycopeptides |
| slr0808 | 16S rRNA processing protein RimM homolog | Translation | Ribosomal proteins |
| slr0810 | hypothetical protein | Hypothetical | N/A |
| slr0812 | hypothetical protein | Hypothetical | N/A |
| slr0813 | hypothetical protein | Hypothetical | N/A |
| slr0815 | hypothetical protein | Hypothetical | N/A |
| slr0816 | hypothetical protein | Hypothetical | N/A |
| slr0817 | salicylate biosynthesis isochorismate synthase | Transport and binding proteins | N/A |
| slr0819 | apolipoprotein N-acyltransferase | Cell envelope | Membranes, lipoproteins, and porins |
| slr0820 | probable glycosyltransferase | Central intermediary metabolism | Polysaccharides and glycoproteins |
| slr0833 | replicative DNA helicase [Contains | DNA replication, restriction, modification, recombination, and repair | N/A |
| slr0846 | hypothetical protein | Hypothetical | N/A |
| slr0847 | phosphopantetheine adenylyltransferase | Cell envelope | Surface polysaccharides, lipopolysaccharides and antigens |
| slr0851 | type 2 NADH dehydrogenase | Photosynthesis and respiration | NADH dehydrogenase |
| slr0852 | hypothetical protein | Hypothetical | N/A |
| slr0853 | ribosomal-protein-alanine acetyltransferase | Translation | Ribosomal proteins |
| slr0856 | putative transposase [ISY100l: 1346125 - 1347070] | Other categories | Transposon-related functions |
| slr0857 | putative transposase [ISY100l: 1346125 - 1347070] | Other categories | Transposon-related functions |
| slr0867 | unknown protein | Unknown | N/A |
| slr0868 | unknown protein | Unknown | N/A |
| slr0870 | hypothetical protein | Hypothetical | N/A |
| slr0871 | unknown protein | Unknown | N/A |
| slr0878 | hypothetical protein | Hypothetical | N/A |
| slr0881 | unknown protein | Unknown | N/A |
| slr0883 | hypothetical protein | Hypothetical | N/A |
| slr0885 | hypothetical protein | Hypothetical | N/A |
| slr0887 | hypothetical protein | Hypothetical | N/A |
| slr0888 | hypothetical protein | Hypothetical | N/A |
| slr0889 | hypothetical protein | Hypothetical | N/A |
| slr0890 | unknown protein | Unknown | N/A |
| slr0895 | transcriptional regulator | Regulatory functions | N/A |
| slr0896 | multi-drug efflux transporter | Transport and binding proteins | N/A |
| slr0903 | molybdopterin (MPT) converting factor, subunit 2 | Biosynthesis of cofactors, prosthetic groups, and carriers | Molybdopterin |
| slr0904 | competence protein ComM homolog | Cellular processes | Transformation |
| slr0905 | Mg-protoporphyrin IX monomethyl ester oxidative cyclase | Biosynthesis of cofactors, prosthetic groups, and carriers | Cobalamin, heme, phycobilin and porphyrin |
| slr0913 | unknown protein | Unknown | N/A |
| slr0915 | putative endonuclease [encoded in trnfM-intron | DNA replication, restriction, modification, recombination, and repair | N/A |
| slr0917 | 7-keto-8-aminopelargonic acid synthetase | Biosynthesis of cofactors, prosthetic groups, and carriers | Biotin |
| slr0918 | methionine aminopeptidase | Translation | Protein modification and translation factors |
| slr0919 | hypothetical protein | Hypothetical | N/A |
| slr0920 | mutator MutT protein | DNA replication, restriction, modification, recombination, and repair | N/A |
| slr0921 | hypothetical protein | Hypothetical | N/A |
| slr0926 | 4-hydroxybenzoate-octaprenyl transferase | Biosynthesis of cofactors, prosthetic groups, and carriers | Menaquinone and ubiquinone |
| slr0935 | hypothetical protein | Hypothetical | N/A |
| slr0936 | nicotinate-nucleotide pyrophosphorylase | Biosynthesis of cofactors, prosthetic groups, and carriers | Nicotinate and nicotinamide |
| slr0944 | multidrug-efflux transporter | Transport and binding proteins | N/A |
| slr0945 | arsenical resistance protein ArsH homolog | Other categories | Drug and analog sensitivity |
| slr0946 | arsenate reductase | Other categories | Drug and analog sensitivity |
| slr0949 | Integral membrane protein of the ABC-type Nat permease for neutral amino acids NatD | Transport and binding proteins | N/A |
| slr0950 | hemolysin-like protein | Cellular processes | Cell killing |
| slr0953 | sucrose-phosphate phosphatase | Energy metabolism | Sugars |
| slr0957 | hypothetical protein | Hypothetical | N/A |
| slr0960 | unknown protein | Unknown | N/A |
| slr0964 | hypothetical protein | Hypothetical | N/A |
| slr0967 | hypothetical protein | Hypothetical | N/A |
| slr0971 | hypothetical protein | Hypothetical | N/A |
| slr0976 | hypothetical protein | Hypothetical | N/A |
| slr0977 | ABC transporter, permease component | Transport and binding proteins | N/A |
| slr0978 | hypothetical protein | Hypothetical | N/A |
| slr0980 | hypothetical protein | Hypothetical | N/A |
| slr0981 | hypothetical protein | Hypothetical | N/A |
| slr0993 | putative peptidase | Cell envelope | Membranes, lipoproteins, and porins |
| slr1019 | phenazine biosynthetic protein PhzF homolog | Other categories | Other |
| slr1023 | unknown protein | Unknown | N/A |
| slr1025 | hypothetical protein | Hypothetical | N/A |
| slr1032 | unknown protein | Unknown | N/A |
| slr1035 | hypothetical protein | Hypothetical | N/A |
| slr1037 | two-component response regulator CheY subfamily | Regulatory functions | N/A |
| slr1039 | hypothetical protein | Hypothetical | N/A |
| slr1041 | two-component response regulator PatA subfamily | Regulatory functions | N/A |
| slr1042 | two-component response regulator CheY subfamily | Regulatory functions | N/A |
| slr1043 | similar to chemotaxis protein CheW | Cellular processes | Chemotaxis |
| slr1046 | putative TatA protein | Hypothetical | N/A |
| slr1050 | hypothetical protein | Hypothetical | N/A |
| slr1052 | hypothetical protein | Hypothetical | N/A |
| slr1062 | unknown protein | Unknown | N/A |
| slr1068 | hypothetical protein | Hypothetical | N/A |
| slr1069 | hypothetical protein | Hypothetical | N/A |
| slr1070 | unknown protein | Unknown | N/A |
| slr1071 | unknown protein | Unknown | N/A |
| slr1074 | unknown protein | Unknown | N/A |
| slr1075 | putative transposase [ISY100b: 378993 - 379939] | Other categories | Transposon-related functions |
| slr1077 | plobable glycosyltransferase | Other categories | Other |
| slr1078 | similar to UDP-glucose 4-epimerase | Energy metabolism | Sugars |
| slr1079 | unknown protein | Unknown | N/A |
| slr1081 | hypothetical protein | Hypothetical | N/A |
| slr1082 | unknown protein | Unknown | N/A |
| slr1083 | hypothetical protein | Hypothetical | N/A |
| slr1087 | hypothetical protein | Hypothetical | N/A |
| slr1093 | 2-amino-4-hydroxy-6-hydroxymethyldihydropteridine pyrophosphokinase | Biosynthesis of cofactors, prosthetic groups, and carriers | Folic acid |
| slr1094 | hypothetical protein | Hypothetical | N/A |
| slr1095 | hypothetical protein | Hypothetical | N/A |
| slr1099 | 3-octaprenyl-4-hydroxybenzoate carboxy-lyase | Biosynthesis of cofactors, prosthetic groups, and carriers | Menaquinone and ubiquinone |
| slr1101 | hypothetical protein | Hypothetical | N/A |
| slr1107 | unknown protein | Unknown | N/A |
| slr1110 | hypothetical protein | Hypothetical | N/A |
| slr1113 | ATP-binding protein of ABC transporter | Transport and binding proteins | N/A |
| slr1114 | hypothetical protein | Hypothetical | N/A |
| slr1115 | probable methyltransferase | Other categories | Other |
| slr1116 | hypothetical protein | Hypothetical | N/A |
| slr1118 | probable UDP-N-acetyl-D-mannosaminuronic acid transferase | Cell envelope | Surface polysaccharides, lipopolysaccharides and antigens |
| slr1119 | hypothetical protein | Hypothetical | N/A |
| slr1120 | type 4 prepilin-like proteins leader peptide processing enzyme | Cellular processes | Chemotaxis |
| slr1125 | probable glucosyl transferase | Biosynthesis of cofactors, prosthetic groups, and carriers | Carotenoid |
| slr1127 | unknown protein | Unknown | N/A |
| slr1130 | ribonuclease HII | Transcription | Degradation of RNA |
| slr1134 | mutator MutT homolog | DNA replication, restriction, modification, recombination, and repair | N/A |
| slr1136 | cytochrome c oxidase subunit II | Photosynthesis and respiration | Respiratory terminal oxidases |
| slr1137 | cytochrome c oxidase subunit I | Photosynthesis and respiration | Respiratory terminal oxidases |
| slr1138 | cytochrome c oxidase subunit III | Photosynthesis and respiration | Respiratory terminal oxidases |
| slr1142 | hypothetical protein | Hypothetical | N/A |
| slr1147 | two-component sensor histidine kinase | Regulatory functions | N/A |
| slr1148 | unknown protein | Unknown | N/A |
| slr1150 | unknown protein | Unknown | N/A |
| slr1152 | hypothetical protein | Hypothetical | N/A |
| slr1162 | unknown protein | Unknown | N/A |
| slr1163 | unknown protein | Unknown | N/A |
| slr1168 | unknown protein | Unknown | N/A |
| slr1170 | hypothetical protein | Hypothetical | N/A |
| slr1174 | hypothetical protein | Hypothetical | N/A |
| slr1177 | hypothetical protein | Hypothetical | N/A |
| slr1182 | hypothetical protein | Hypothetical | N/A |
| slr1183 | hypothetical protein | Hypothetical | N/A |
| slr1184 | hypothetical protein | Hypothetical | N/A |
| slr1185 | cytochrome b6f complex alternative iron-sulfur subunit (Rieske iron sulfur protein) | Photosynthesis and respiration | Cytochrome b6/f complex |
| slr1187 | unknown protein | Unknown | N/A |
| slr1188 | hypothetical protein | Hypothetical | N/A |
| slr1189 | unknown protein | Unknown | N/A |
| slr1197 | SMF protein | Other categories | Other |
| slr1199 | DNA mismatch repair protein MutL | DNA replication, restriction, modification, recombination, and repair | N/A |
| slr1200 | urea transport system permease protein | Transport and binding proteins | N/A |
| slr1203 | hypothetical protein | Hypothetical | N/A |
| slr1209 | hypothetical protein | Hypothetical | N/A |
| slr1210 | unknown protein | Unknown | N/A |
| slr1213 | two-component response regulator AraC subfamily | Regulatory functions | N/A |
| slr1214 | two-component response regulator PatA subfamily | Regulatory functions | N/A |
| slr1215 | hypothetical protein | Hypothetical | N/A |
| slr1218 | hypothetical protein YCF39 | Hypothetical | N/A |
| slr1219 | urease accessory protein E | Central intermediary metabolism | Other |
| slr1222 | unknown protein | Unknown | N/A |
| slr1229 | sulfate permease | Transport and binding proteins | N/A |
| slr1230 | hypothetical protein | Hypothetical | N/A |
| slr1232 | unknown protein | Unknown | N/A |
| slr1236 | hypothetical protein | Hypothetical | N/A |
| slr1241 | hypothetical protein | Hypothetical | N/A |
| slr1245 | transcriptional regulator | Regulatory functions | N/A |
| slr1246 | putative transposase [ISY802b(partial copy): 1384736 - 1385513] | Other categories | Transposon-related functions |
| slr1248 | phosphate transport system permease protein PstC homolog | Transport and binding proteins | N/A |
| slr1249 | phosphate transport system permease protein PstA homolog | Transport and binding proteins | N/A |
| slr1253 | unknown protein | Unknown | N/A |
| slr1256 | urease gamma subunit | Central intermediary metabolism | Other |
| slr1260 | hypothetical protein | Hypothetical | N/A |
| slr1262 | hypothetical protein | Hypothetical | N/A |
| slr1266 | hypothetical protein | Hypothetical | N/A |
| slr1271 | probable UDP-N-acetyl-D-mannosaminuronic acid transferase | Cell envelope | Surface polysaccharides, lipopolysaccharides and antigens |
| slr1278 | hypothetical protein YCF62 | Hypothetical | N/A |
| slr1279 | NADH dehydrogenase subunit 3 | Photosynthesis and respiration | NADH dehydrogenase |
| slr1282 | putative transposase [ISY508b: 1877114 - 1878081] | Other categories | Transposon-related functions |
| slr1283 | putative transposase [ISY508b: 1877114 - 1878081] | Other categories | Transposon-related functions |
| slr1285 | two-component sensor histidine kinase | Regulatory functions | N/A |
| slr1288 | hypothetical protein | Hypothetical | N/A |
| slr1290 | hypothetical protein | Hypothetical | N/A |
| slr1291 | NADH dehydrogenase subunit 4 | Photosynthesis and respiration | NADH dehydrogenase |
| slr1293 | similar to to phytoene dehydrogenase | Biosynthesis of cofactors, prosthetic groups, and carriers | Carotenoid |
| slr1300 | similar to 2-octaprenyl-6-methoxyphenol hydroxylase | Biosynthesis of cofactors, prosthetic groups, and carriers | Menaquinone and ubiquinone |
| slr1303 | hypothetical protein | Hypothetical | N/A |
| slr1307 | hypothetical protein | Hypothetical | N/A |
| slr1315 | hypothetical protein | Hypothetical | N/A |
| slr1316 | ABC-type iron(III) dicitrate transport system permease protein | Transport and binding proteins | N/A |
| slr1317 | ABC-type iron(III) dicitrate transport system permease protein | Transport and binding proteins | N/A |
| slr1318 | iron(III) dicitrate transport system ATP-binding protein | Transport and binding proteins | N/A |
| slr1327 | hypothetical protein | Hypothetical | N/A |
| slr1332 | beta ketoacyl-acyl carrier protein synthase | Fatty acid, phospholipid and sterol metabolism | N/A |
| slr1336 | H+/Ca2+ exchanger | Transport and binding proteins | N/A |
| slr1339 | hypothetical protein | Hypothetical | N/A |
| slr1340 | unknown protein | Unknown | N/A |
| slr1343 | hypothetical protein | Hypothetical | N/A |
| slr1344 | hypothetical protein | Hypothetical | N/A |
| slr1357 | putative transposase [ISY100c: 1098251 - 1099197] | Other categories | Transposon-related functions |
| slr1362 | hypothetical protein | Hypothetical | N/A |
| slr1365 | hypothetical protein | Hypothetical | N/A |
| slr1366 | lipoprotein signal peptidase (signal peptidase II) | Cellular processes | Protein and peptide secretion |
| slr1376 | hypothetical protein | Hypothetical | N/A |
| slr1379 | quinol oxidase subunit I | Photosynthesis and respiration | Respiratory terminal oxidases |
| slr1380 | quinol oxidase subunit II | Photosynthesis and respiration | Respiratory terminal oxidases |
| slr1383 | unknown protein | Unknown | N/A |
| slr1384 | hypothetical protein | Hypothetical | N/A |
| slr1391 | unknown protein | Unknown | N/A |
| slr1392 | ferrous iron transport protein B | Transport and binding proteins | N/A |
| slr1394 | hypothetical protein | Hypothetical | N/A |
| slr1396 | unknown protein | Unknown | N/A |
| slr1400 | two-component hybrid sensor and regulator | Regulatory functions | N/A |
| slr1414 | two-component sensor histidine kinase | Regulatory functions | N/A |
| slr1417 | hypothetical protein YCF57 | Hypothetical | N/A |
| slr1419 | hypothetical protein | Hypothetical | N/A |
| slr1420 | probable sugar kinase | Other categories | Other |
| slr1421 | unknown protein | Unknown | N/A |
| slr1425 | hypothetical protein | Hypothetical | N/A |
| slr1429 | hypothetical protein | Hypothetical | N/A |
| slr1436 | unknown protein | Unknown | N/A |
| slr1440 | hypothetical protein | Hypothetical | N/A |
| slr1441 | hypothetical protein | Hypothetical | N/A |
| slr1442 | hypothetical protein | Hypothetical | N/A |
| slr1443 | serine/threonine kinase | Regulatory functions | N/A |
| slr1444 | hypothetical protein | Hypothetical | N/A |
| slr1450 | unknown protein | Unknown | N/A |
| slr1451 | hypothetical protein | Hypothetical | N/A |
| slr1453 | sulfate transport system permease protein | Transport and binding proteins | N/A |
| slr1454 | sulfate transport system permease protein | Transport and binding proteins | N/A |
| slr1455 | sulfate transport system ATP-binding protein | Transport and binding proteins | N/A |
| slr1456 | type 4 pilin-like protein, or general secretion pathway protein G | Unknown | N/A |
| slr1457 | chromate transport protein | Transport and binding proteins | N/A |
| slr1464 | hypothetical protein | Hypothetical | N/A |
| slr1468 | hypothetical protein | Hypothetical | N/A |
| slr1469 | protein subunit of ribonuclease P (RNase P) | Transcription | RNA synthesis, modification, and DNA transcription |
| slr1470 | hypothetical protein | Hypothetical | N/A |
| slr1472 | hypothetical protein | Hypothetical | N/A |
| slr1475 | unknown protein | Unknown | N/A |
| slr1484 | unknown protein | Unknown | N/A |
| slr1488 | multidrug resistance family ABC transporter | Transport and binding proteins | N/A |
| slr1489 | transcriptional regulator | Regulatory functions | N/A |
| slr1491 | iron(III) dicitrate transport system substrate-binding protein | Transport and binding proteins | N/A |
| slr1492 | iron(III) dicitrate transport system substrate-binding protein | Transport and binding proteins | N/A |
| slr1493 | hypothetical protein | Hypothetical | N/A |
| slr1494 | MDR (multidrug resistance) family ABC transporter | Transport and binding proteins | N/A |
| slr1495 | hypothetical protein | Hypothetical | N/A |
| slr1496 | putative transposase: | Other categories | Transposon-related functions |
| slr1498 | putative hydrogenase expression/formation protein HypD | Other categories | Hydrogenase |
| slr1508 | probable glycosyltransferase | Other categories | Other |
| slr1509 | membrane subunit of a Ktr-like ion transport system | Transport and binding proteins | N/A |
| slr1515 | putative membrane protein required for bicarbonate uptake | Hypothetical | N/A |
| slr1518 | phylloquinone biosynthesis protein, probable 1,4-dihydroxy-2-naphthoic acid phytyltransferase | Biosynthesis of cofactors, prosthetic groups, and carriers | Menaquinone and ubiquinone |
| slr1520 | oxidoreductase, aldo/keto reductase family | Other categories | Other |
| slr1522 | putative transposase [ISY352d: 1614422 - 1615835] | Other categories | Transposon-related functions |
| slr1523 | putative transposase: | Other categories | Transposon-related functions |
| slr1524 | putative transposase [ISY100u(partial copy): 1616832 - 1617509] | Other categories | Transposon-related functions |
| slr1529 | nitrogen assimilation regulatory protein | Amino acid biosynthesis | Glutamate family / Nitrogen assimilation |
| slr1530 | hypothetical protein | Hypothetical | N/A |
| slr1533 | hypothetical protein | Hypothetical | N/A |
| slr1538 | cobalamin biosynthesis protein D | Biosynthesis of cofactors, prosthetic groups, and carriers | Cobalamin, heme, phycobilin and porphyrin |
| slr1543 | DNA-damage-inducible protein F | DNA replication, restriction, modification, recombination, and repair | N/A |
| slr1544 | unknown protein | Unknown | N/A |
| slr1545 | RNA polymerase ECF-type (group 3) sigma-E factor | Transcription | RNA synthesis, modification, and DNA transcription |
| slr1546 | hypothetical protein | Hypothetical | N/A |
| slr1547 | hypothetical protein | Hypothetical | N/A |
| slr1549 | polypeptide deformylase | Translation | Protein modification and translation factors |
| slr1564 | group 3 RNA polymerase sigma factor | Transcription | RNA synthesis, modification, and DNA transcription |
| slr1565 | hypothetical protein | Hypothetical | N/A |
| slr1567 | unknown protein | Unknown | N/A |
| slr1568 | hypothetical protein | Hypothetical | N/A |
| slr1572 | hypothetical protein | Hypothetical | N/A |
| slr1576 | unknown protein | Unknown | N/A |
| slr1583 | hypothetical protein | Hypothetical | N/A |
| slr1584 | two-component transcription regulator OmpR subfamily | Regulatory functions | N/A |
| slr1585 | putative transposase [ISY508c(partial copy): 3405449 - 3406337] | Other categories | Transposon-related functions |
| slr1586 | putative transposase [ISY508c(partial copy): 3405449 - 3406337] | Other categories | Transposon-related functions |
| slr1591 | hypothetical protein | Hypothetical | N/A |
| slr1592 | probable pseudouridine synthase | Translation | Aminoacyl tRNA synthetases and tRNA modification |
| slr1593 | hypothetical protein | Hypothetical | N/A |
| slr1595 | Na+/H+ antiporter | Transport and binding proteins | N/A |
| slr1599 | hypothetical protein | Hypothetical | N/A |
| slr1600 | hypothetical protein | Hypothetical | N/A |
| slr1601 | hypothetical protein | Hypothetical | N/A |
| slr1614 | hypothetical protein | Hypothetical | N/A |
| slr1627 | unknown protein | Unknown | N/A |
| slr1629 | ribosomal large subunit pseudouridine synthase D | Translation | Ribosomal proteins |
| slr1634 | hypothetical protein | Hypothetical | N/A |
| slr1635 | putative transposase [ISY203e: 2048410 - 2049583] | Other categories | Transposon-related functions |
| slr1636 | unknown protein | Unknown | N/A |
| slr1638 | hypothetical protein | Hypothetical | N/A |
| slr1646 | ribonuclease III | Transcription | Degradation of RNA |
| slr1652 | hypothetical protein | Hypothetical | N/A |
| slr1653 | N-acyl-L-amino acid amidohydrolase | Amino acid biosynthesis | Glutamate family / Nitrogen assimilation |
| slr1660 | hypothetical protein | Hypothetical | N/A |
| slr1664 | hypothetical protein | Hypothetical | N/A |
| slr1667 | hypothetical protein | Unknown | N/A |
| slr1670 | unknown protein | Unknown | N/A |
| slr1673 | probable tRNA/rRNA methyltransferase | Translation | Aminoacyl tRNA synthetases and tRNA modification |
| slr1674 | hypothetical protein | Hypothetical | N/A |
| slr1675 | putative hydrogenase expression/formation protein HypA1 | Other categories | Hydrogenase |
| slr1676 | hypothetical protein | Hypothetical | N/A |
| slr1677 | hypothetical protein | Hypothetical | N/A |
| slr1682 | putative transposase [ISY391b: 1970517 - 1970880, join 1972064 - 1973077] | Other categories | Transposon-related functions |
| slr1683 | putative transposase [ISY391b: 1970517 - 1970880, join 1972064 - 1973077] | Other categories | Transposon-related functions |
| slr1684 | putative transposase [ISY391b: 1970517 - 1970880, join 1972064 - 1973077] | Other categories | Transposon-related functions |
| slr1690 | hypothetical protein | Hypothetical | N/A |
| slr1704 | hypothetical protein | Hypothetical | N/A |
| slr1705 | aspartoacylase | Energy metabolism | Amino acids and amines |
| slr1706 | dihydroflavonol 4-reductase | Biosynthesis of cofactors, prosthetic groups, and carriers | Others |
| slr1708 | probable peptidase | Cell envelope | Murein sacculus and peptidoglycan |
| slr1715 | putative transposase [ISY100m: 1463383 - 1464328] | Other categories | Transposon-related functions |
| slr1716 | putative transposase [ISY100m: 1463383 - 1464328] | Other categories | Transposon-related functions |
| slr1723 | permease protein of sugar ABC transporter | Transport and binding proteins | N/A |
| slr1724 | hypothetical protein | Hypothetical | N/A |
| slr1726 | unknown protein | Unknown | N/A |
| slr1728 | potassium-transporting P-type ATPase A chain | Transport and binding proteins | N/A |
| slr1731 | potassium-transporting P-type ATPase D chain | Transport and binding proteins | N/A |
| slr1736 | homogentisate phytyltransferase | Hypothetical | N/A |
| slr1737 | hypothetical protein | Hypothetical | N/A |
| slr1738 | transcription regulator Fur family | Regulatory functions | N/A |
| slr1746 | glutamate racemase | Cell envelope | Murein sacculus and peptidoglycan |
| slr1747 | cell death suppressor protein Lls1 homolog | Cellular processes | Cell killing |
| slr1752 | hypothetical protein | Hypothetical | N/A |
| slr1760 | two-component response regulator | Regulatory functions | N/A |
| slr1764 | similar to tellurium resistance protein TerE | Other categories | Drug and analog sensitivity |
| slr1767 | hypothetical protein | Hypothetical | N/A |
| slr1770 | hypothetical protein | Hypothetical | N/A |
| slr1771 | unknown protein | Unknown | N/A |
| slr1773 | unknown protein | Unknown | N/A |
| slr1774 | unknown protein | Unknown | N/A |
| slr1776 | high affinity sulfate transporter | Transport and binding proteins | N/A |
| slr1778 | unknown protein | Unknown | N/A |
| slr1787 | thiamine-monophosphate kinase | Biosynthesis of cofactors, prosthetic groups, and carriers | Thiamin |
| slr1789 | unknown protein | Unknown | N/A |
| slr1798 | unknown protein | Unknown | N/A |
| slr1803 | adenine-specific DNA metylase | DNA replication, restriction, modification, recombination, and repair | N/A |
| slr1804 | unknown protein | Unknown | N/A |
| slr1805 | two-component sensor histidine kinase | Regulatory functions | N/A |
| slr1807 | hypothetical protein | Hypothetical | N/A |
| slr1809 | unknown protein | Unknown | N/A |
| slr1811 | hypothetical protein | Hypothetical | N/A |
| slr1812 | hypothetical protein | Hypothetical | N/A |
| slr1813 | hypothetical protein | Hypothetical | N/A |
| slr1818 | hypothetical protein | Hypothetical | N/A |
| slr1820 | hypothetical protein | Hypothetical | N/A |
| slr1822 | endonuclease III | DNA replication, restriction, modification, recombination, and repair | N/A |
| slr1826 | hypothetical protein | Hypothetical | N/A |
| slr1827 | hypothetical protein | Hypothetical | N/A |
| slr1830 | poly(3-hydroxyalkanoate) synthase | Central intermediary metabolism | Polysaccharides and glycoproteins |
| slr1838 | carbon dioxide concentrating mechanism protein CcmK homolog 3, putative carboxysome assembly protein | Photosynthesis and respiration | CO2 fixation |
| slr1840 | hypothetical protein | Hypothetical | N/A |
| slr1846 | hypothetical protein YCF64 | Biosynthesis of cofactors, prosthetic groups, and carriers | Thioredoxin, glutaredoxin, and glutathione |
| slr1847 | hypothetical protein | Hypothetical | N/A |
| slr1851 | hypothetical protein | Hypothetical | N/A |
| slr1861 | probable sigma regulatory factor | Transcription | RNA synthesis, modification, and DNA transcription |
| slr1862 | unknown protein | Unknown | N/A |
| slr1864 | hypothetical protein | Hypothetical | N/A |
| slr1865 | unknown protein | Unknown | N/A |
| slr1866 | unknown protein | Unknown | N/A |
| slr1869 | unknown protein | Unknown | N/A |
| slr1870 | hypothetical protein | Hypothetical | N/A |
| slr1871 | transcriptional regulator | Regulatory functions | N/A |
| slr1876 | hypothetical protein | Hypothetical | N/A |
| slr1879 | precorrin-2 methyltransferase | Biosynthesis of cofactors, prosthetic groups, and carriers | Cobalamin, heme, phycobilin and porphyrin |
| slr1885 | hypothetical protein | Hypothetical | N/A |
| slr1886 | hypothetical protein | Hypothetical | N/A |
| slr1895 | hypothetical protein | Hypothetical | N/A |
| slr1896 | hypothetical protein | Hypothetical | N/A |
| slr1902 | putative transposase [ISY120a: 851653 - 852454] | Other categories | Transposon-related functions |
| slr1903 | putative transposase [ISY120a: 851653 - 852454] | Other categories | Transposon-related functions |
| slr1907 | hypothetical protein | Hypothetical | N/A |
| slr1910 | probable N-acetylmuramoyl-L-alanine amidase | Cell envelope | Murein sacculus and peptidoglycan |
| slr1911 | hypothetical protein | Hypothetical | N/A |
| slr1912 | putative PP2C-type protein phosphatase | Transcription | RNA synthesis, modification, and DNA transcription |
| slr1913 | hypothetical protein | Hypothetical | N/A |
| slr1914 | hypothetical protein | Hypothetical | N/A |
| slr1915 | hypothetical protein | Hypothetical | N/A |
| slr1917 | hypothetical protein | Hypothetical | N/A |
| slr1926 | hypothetical protein | Hypothetical | N/A |
| slr1927 | hypothetical protein | Hypothetical | N/A |
| slr1928 | type 4 pilin-like protein | Unknown | N/A |
| slr1929 | type 4 pilin-like protein | Cellular processes | Chemotaxis |
| slr1930 | type 4 pilin-like protein | Unknown | N/A |
| slr1931 | type 4 pilin-like protein | Unknown | N/A |
| slr1932 | unknown protein | Unknown | N/A |
| slr1935 | hypothetical protein | Hypothetical | N/A |
| slr1936 | putative transposase [ISY100r: 2235489 - 2236434] | Other categories | Transposon-related functions |
| slr1937 | putative transposase [ISY100r: 2235489 - 2236434] | Other categories | Transposon-related functions |
| slr1946 | hypothetical protein | Hypothetical | N/A |
| slr1957 | hypothetical protein | Hypothetical | N/A |
| slr1960 | putative transposase [ISY391a(partial copy): 1762937 - 1763383] | Other categories | Transposon-related functions |
| slr1964 | hypothetical protein | Hypothetical | N/A |
| slr1966 | hypothetical protein | Hypothetical | N/A |
| slr1971 | hypothetical protein | Hypothetical | N/A |
| slr1972 | hypothetical protein YCF81 | Hypothetical | N/A |
| slr1977 | hypothetical protein | Hypothetical | N/A |
| slr1979 | anthranilate synthase component I | Amino acid biosynthesis | Aromatic amino acid family |
| slr1980 | unknown protein | Unknown | N/A |
| slr1982 | two-component response regulator CheY subfamily | Regulatory functions | N/A |
| slr1990 | hypothetical protein | Hypothetical | N/A |
| slr1991 | adenylate cyclase | Regulatory functions | N/A |
| slr1999 | hypothetical protein | Hypothetical | N/A |
| slr2003 | hypothetical protein | Hypothetical | N/A |
| slr2006 | hypothetical protein | Hypothetical | N/A |
| slr2007 | NADH dehydrogenase subunit 4 | Photosynthesis and respiration | NADH dehydrogenase |
| slr2008 | hypothetical protein | Hypothetical | N/A |
| slr2009 | NADH dehydrogenase subunit 4 | Photosynthesis and respiration | NADH dehydrogenase |
| slr2010 | hypothetical protein | Hypothetical | N/A |
| slr2012 | hypothetical protein | Hypothetical | N/A |
| slr2013 | hypothetical protein | Hypothetical | N/A |
| slr2016 | type 4 pilin-like protein, essential for motility | Unknown | N/A |
| slr2026 | dihydropteroate synthase | Biosynthesis of cofactors, prosthetic groups, and carriers | Folic acid |
| slr2027 | unknown protein | Unknown | N/A |
| slr2030 | hypothetical protein | Hypothetical | N/A |
| slr2031 | putative PP2C-type protein phosphatase, gene required to recover from the nitrogen or sulfate starvation induced stationary phase | Hypothetical | N/A |
| slr2033 | membrane-associated rubredoxin, essential for photosystem I assembly | Fatty acid, phospholipid and sterol metabolism | N/A |
| slr2036 | putative transposase [ISY203a: 573408 - 574580] | Other categories | Transposon-related functions |
| slr2037 | unknown protein | Unknown | N/A |
| slr2038 | hypothetical protein | Hypothetical | N/A |
| slr2041 | probable two-component response regulator | Regulatory functions | N/A |
| slr2042 | hypothetical protein | Hypothetical | N/A |
| slr2043 | zinc transport system substrate-binding protein | Transport and binding proteins | N/A |
| slr2045 | zinc transport system permease protein | Transport and binding proteins | N/A |
| slr2046 | unknown protein | Unknown | N/A |
| slr2052 | hypothetical protein | Hypothetical | N/A |
| slr2057 | water channel protein | Transport and binding proteins | N/A |
| slr2059 | iron-sulfur cluster binding protein homolog | Photosynthesis and respiration | Soluble electron carriers |
| slr2062 | putative transposase [ISY052a: 1420842 - 1422331] | Other categories | Transposon-related functions |
| slr2071 | unknown protein | Unknown | N/A |
| slr2074 | similar to mannose-1-phosphate guanylyltransferase | Energy metabolism | Sugars |
| slr2077 | probable ABC transporter, periplasmic binding protein | Transport and binding proteins | N/A |
| slr2078 | hypothetical protein | Hypothetical | N/A |
| slr2080 | hypothetical protein | Hypothetical | N/A |
| slr2082 | cytochrome c oxidase subunit I | Photosynthesis and respiration | Respiratory terminal oxidases |
| slr2083 | cytochrome c oxidase subunit III | Photosynthesis and respiration | Respiratory terminal oxidases |
| slr2084 | hypothetical protein | Hypothetical | N/A |
| slr2092 | hypothetical protein | Hypothetical | N/A |
| slr2095 | putative transposase [ISY120c: 1561629 - 1562430] | Other categories | Transposon-related functions |
| slr2096 | putative transposase [ISY120c: 1561629 - 1562430] | Other categories | Transposon-related functions |
| slr2097 | cyanoglobin | Other categories | Other |
| slr2099 | two-component hybrid sensor and regulator | Regulatory functions | N/A |
| slr2103 | hypothetical protein | Hypothetical | N/A |
| slr2107 | probable polysaccharide ABC transporter permease protein | Transport and binding proteins | N/A |
| slr2108 | probable polysaccharide ABC transporter ATP binding subunit | Transport and binding proteins | N/A |
| slr2110 | unknown protein | Unknown | N/A |
| slr2111 | unknown protein | Unknown | N/A |
| slr2112 | putative transposase [ISY100o: 1626093 - 1627038] | Other categories | Transposon-related functions |
| slr2113 | putative transposase [ISY100o: 1626093 - 1627038] | Other categories | Transposon-related functions |
| slr2114 | perosamine synthetase | Cell envelope | Surface polysaccharides, lipopolysaccharides and antigens |
| slr2117 | hypothetical protein | Hypothetical | N/A |
| slr2118 | unknown protein | Unknown | N/A |
| slr2119 | unknown protein | Unknown | N/A |
| slr2120 | hypothetical protein | Hypothetical | N/A |
| slr2121 | hypothetical protein | Hypothetical | N/A |
| slr2124 | 3-oxoacyl-[acyl-carrier protein] reductase | Fatty acid, phospholipid and sterol metabolism | N/A |
| slr2125 | hypothetical protein | Hypothetical | N/A |
| slr2126 | probable glycosyltransferase | Other categories | Other |
| slr2127 | hypothetical protein | Hypothetical | N/A |
| slr2128 | hypothetical protein | Hypothetical | N/A |
| slr2135 | hydrogenase accessory protein HupE | Other categories | Hydrogenase |
| slr5005 | hypothetical protein | Hypothetical | N/A |
| slr5010 | integrase/recombinase: | Other categories | Transposon-related functions |
| slr5012 | hypothetical protein | Hypothetical | N/A |
| slr5013 | unknown protein | Unknown | N/A |
| slr5016 | unknown protein | Unknown | N/A |
| slr5017 | hypothetical protein | Hypothetical | N/A |
| slr5018 | hypothetical protein | Hypothetical | N/A |
| slr5021 | hypothetical protein | Hypothetical | N/A |
| slr5022 | probable aminotransferase | Other categories | Other |
| slr5023 | hypothetical protein | Hypothetical | N/A |
| slr5024 | hypothetical protein | Hypothetical | N/A |
| slr5029 | putative transposase [ISY391d(partial copy): 31333 - 32206] | Other categories | Transposon-related functions |
| slr5037 | hypothetical protein | Hypothetical | N/A |
| slr5038 | chromate transporter | Transport and binding proteins | N/A |
| slr5040 | putative transposase [ISY523u: 38789 - 39659] | Other categories | Transposon-related functions |
| slr5051 | unknown protein | Unknown | N/A |
| slr5053 | unknown protein | Unknown | N/A |
| slr5054 | probable glycosyltransferase | Other categories | Other |
| slr5055 | similar to UDP-N-acetyl-D-mannosaminuronic acid transferase | Other categories | Other |
| slr5056 | probable glycosyltransferase | Other categories | Other |
| slr5058 | hypothetical protein | Hypothetical | N/A |
| slr5071 | unknown protein | Unknown | N/A |
| slr5073 | unknown protein | Unknown | N/A |
| slr5077 | hypothetical protein | Hypothetical | N/A |
| slr5078 | similar to potassium channel protein | Transport and binding proteins | N/A |
| slr5082 | hypothetical protein | Hypothetical | N/A |
| slr5085 | unknown protein | Unknown | N/A |
| slr5087 | hypothetical protein | Hypothetical | N/A |
| slr5088 | probable short-chain dehydrogenase | Other categories | Other |
| slr5093 | probable flavin-containing amine oxidase | Other categories | Other |
| slr5101 | hypothetical protein | Hypothetical | N/A |
| slr5102 | hypothetical protein | Hypothetical | N/A |
| slr5105 | plasmid partitioning protein, ParA family | Other categories | Other |
| slr5110 | unknown protein | Unknown | N/A |
| slr5111 | unknown protein | Unknown | N/A |
| slr5112 | unknown protein | Unknown | N/A |
| slr5115 | hypothetical protein | Hypothetical | N/A |
| slr5116 | hypothetical protein | Hypothetical | N/A |
| slr5118 | hypothetical protein | Hypothetical | N/A |
| slr5119 | hypothetical protein | Hypothetical | N/A |
| slr5124 | hypothetical protein | Hypothetical | N/A |
| slr5126 | unknown protein | Unknown | N/A |
| slr5127 | unknown protein | Unknown | N/A |
| slr6001 | two-component hybrid sensor and regulator | Regulatory functions | N/A |
| slr6004 | unknown protein | Unknown | N/A |
| slr6005 | unknown protein | Unknown | N/A |
| slr6006 | unknown protein | Unknown | N/A |
| slr6007 | unknown protein | Unknown | N/A |
| slr6008 | unknown protein | Unknown | N/A |
| slr6009 | unknown protein | Unknown | N/A |
| slr6011 | probable nuclease | Other categories | Other |
| slr6012 | unknown protein | Unknown | N/A |
| slr6013 | unknown protein | Unknown | N/A |
| slr6014 | unknown protein | Unknown | N/A |
| slr6015 | unknown protein | Unknown | N/A |
| slr6016 | unknown protein | Unknown | N/A |
| slr6021 | unknown protein | Unknown | N/A |
| slr6022 | unknown protein | Unknown | N/A |
| slr6025 | probable antirestriction protein | DNA replication, restriction, modification, recombination, and repair | N/A |
| slr6028 | unknown protein | Unknown | N/A |
| slr6029 | hypothetical protein | Hypothetical | N/A |
| slr6031 | unknown protein | Unknown | N/A |
| slr6033 | unknown protein | Unknown | N/A |
| slr6034 | cytidine deaminase | Purines, pyrimidines, nucleosides, and nucleotides | Interconversions and salvage of nucleosides and nucleotides |
| slr6037 | arsenate reductase | Cellular processes | Detoxification |
| slr6038 | hypothetical protein | Hypothetical | N/A |
| slr6039 | hypothetical protein | Hypothetical | N/A |
| slr6040 | two-component response regulator | Regulatory functions | N/A |
| slr6041 | two-component sensor histidine kinase | Regulatory functions | N/A |
| slr6042 | probable cation efflux system protein, czcB homolog | Transport and binding proteins | N/A |
| slr6043 | probable cation efflux system protein, czcA homolog | Transport and binding proteins | N/A |
| slr6044 | hypothetical protein | Hypothetical | N/A |
| slr6045 | unknown protein | Unknown | N/A |
| slr6047 | hypothetical protein | Hypothetical | N/A |
| slr6049 | hypothetical protein | Hypothetical | N/A |
| slr6050 | hypothetical protein | Hypothetical | N/A |
| slr6051 | hypothetical protein | Hypothetical | N/A |
| slr6056 | probable transcriptional regulator | Regulatory functions | N/A |
| slr6057 | hypothetical protein | Hypothetical | N/A |
| slr6058 | hypothetical protein | Hypothetical | N/A |
| slr6063 | unknown protein | Unknown | N/A |
| slr6064 | unknown protein | Unknown | N/A |
| slr6065 | unknown protein | Unknown | N/A |
| slr6066 | unknown protein | Unknown | N/A |
| slr6067 | unknown protein | Unknown | N/A |
| slr6068 | unknown protein | Unknown | N/A |
| slr6070 | probable nuclease | Other categories | Other |
| slr6072 | unknown protein | Unknown | N/A |
| slr6073 | unknown protein | Unknown | N/A |
| slr6074 | unknown protein | Unknown | N/A |
| slr6075 | unknown protein | Unknown | N/A |
| slr6080 | unknown protein | Unknown | N/A |
| slr6081 | unknown protein | Unknown | N/A |
| slr6084 | probable antirestriction protein | DNA replication, restriction, modification, recombination, and repair | N/A |
| slr6087 | unknown protein | Unknown | N/A |
| slr6088 | hypothetical protein | Hypothetical | N/A |
| slr6090 | unknown protein | Unknown | N/A |
| slr6091 | unknown protein | Unknown | N/A |
| slr6094 | hypothetical protein | Hypothetical | N/A |
| slr6095 | type I restriction-modification system, M subunit (fragment) | DNA replication, restriction, modification, recombination, and repair | N/A |
| slr6096 | type I restriction-modification system, M subunit (fragment) | DNA replication, restriction, modification, recombination, and repair | N/A |
| slr6097 | type I site-specific deoxyribonuclease | DNA replication, restriction, modification, recombination, and repair | N/A |
| slr6100 | hypothetical protein | Hypothetical | N/A |
| slr6101 | hypothetical protein | Hypothetical | N/A |
| slr6102 | type I restriction-modification system, R subunit | DNA replication, restriction, modification, recombination, and repair | N/A |
| slr6103 | hypothetical protein | Hypothetical | N/A |
| slr6104 | hypothetical protein | Hypothetical | N/A |
| slr6105 | putative transposase [ISY203x: 99448 - 100621] | Other categories | Transposon-related functions |
| slr6106 | hypothetical protein | Hypothetical | N/A |
| slr6107 | hypothetical protein | Hypothetical | N/A |
| slr6108 | hypothetical protein | Hypothetical | N/A |
| slr6110 | putative signalling protein | Regulatory functions | N/A |
| slr7005 | integrase/recombinase: | Other categories | Transposon-related functions |
| slr7008 | putative transposase [ISY203k: 4154 - 5327] | Other categories | Transposon-related functions |
| slr7010 | unknown protein | Unknown | N/A |
| slr7011 | unknown protein | Unknown | N/A |
| slr7012 | hypothetical protein | Hypothetical | N/A |
| slr7013 | hypothetical protein | Hypothetical | N/A |
| slr7014 | unknown protein | Unknown | N/A |
| slr7015 | hypothetical protein | Hypothetical | N/A |
| slr7016 | hypothetical protein | Hypothetical | N/A |
| slr7023 | hypothetical protein | Hypothetical | N/A |
| slr7024 | hypothetical protein | Hypothetical | N/A |
| slr7025 | hypothetical protein | Hypothetical | N/A |
| slr7026 | unknown protein | Unknown | N/A |
| slr7032 | hypothetical protein | Hypothetical | N/A |
| slr7037 | hypothetical protein | Hypothetical | N/A |
| slr7041 | probable growth inhibitor, PemK-like protein | Other categories | Other |
| slr7049 | resolvase: | Other categories | Transposon-related functions |
| slr7052 | unknown protein | Unknown | N/A |
| slr7054 | unknown protein | Unknown | N/A |
| slr7057 | unknown protein | Unknown | N/A |
| slr7058 | hypothetical protein | Hypothetical | N/A |
| slr7059 | hypothetical protein | Hypothetical | N/A |
| slr7060 | hypothetical protein | Hypothetical | N/A |
| slr7061 | unknown protein | Unknown | N/A |
| slr7068 | hypothetical protein | Hypothetical | N/A |
| slr7071 | hypothetical protein | Hypothetical | N/A |
| slr7073 | hypothetical protein | Hypothetical | N/A |
| slr7076 | hypothetical protein | Hypothetical | N/A |
| slr7080 | unknown protein | Unknown | N/A |
| slr7081 | unknown protein | Unknown | N/A |
| slr7082 | unknown protein | Unknown | N/A |
| slr7083 | unknown protein | Unknown | N/A |
| slr7088 | hypothetical protein | Hypothetical | N/A |
| slr7091 | hypothetical protein | Hypothetical | N/A |
| slr7092 | hypothetical protein | Hypothetical | N/A |
| slr7095 | hypothetical protein | Hypothetical | N/A |
| slr7096 | hypothetical protein | Hypothetical | N/A |
| slr7097 | hypothetical protein | Hypothetical | N/A |
| slr7098 | unknown protein | Unknown | N/A |
| slr7099 | unknown protein | Unknown | N/A |
| slr7100 | unknown protein | Unknown | N/A |
| slr7101 | unknown protein | Unknown | N/A |
| slr7102 | hypothetical protein | Hypothetical | N/A |
| slr7104 | putative transposase [ISY100w: 100709 - 101654] | Other categories | Transposon-related functions |
| slr7105 | putative transposase [ISY100w: 100709 - 101654] | Other categories | Transposon-related functions |
| slr8014 | hypothetical protein | Hypothetical | N/A |
| slr8015 | plasmid partitioning protein, ParA family | Other categories | Other |
| slr8016 | plasmid partitioning protein, ParB | Other categories | Other |
| slr8021 | hypothetical protein | Hypothetical | N/A |
| slr8022 | hypothetical protein | Hypothetical | N/A |
| slr8023 | probable esterase | Other categories | Other |
| slr8026 | transcriptional regulatory protein MarR family | Regulatory functions | N/A |
| slr8029 | resolvase: | Other categories | Transposon-related functions |
| slr8030 | hypothetical protein | Hypothetical | N/A |
| slr8036 | probable acetyltransferase | Other categories | Other |
| slr8037 | probable acetyltransferase | Other categories | Other |
| slr8038 | WD-repeat protein | Other categories | WD repeat proteins |
| slr8044 | unknown protein | Unknown | N/A |
| slr8045 | putative transposase [ISY100x: 40984 - 41929] | Other categories | Transposon-related functions |
| slr8046 | putative transposase [ISY100x: 40984 - 41929] | Other categories | Transposon-related functions |
| slr9002 | unknown protein [ORF-A] | Unknown | N/A |
| slr9003 | hypothetical protein | Hypothetical | N/A |
| slr9101 | replication protein A | DNA replication, restriction, modification, recombination, and repair | N/A |
| slr9102 | unknown protein [ORF2] | Unknown | N/A |
| slr9201 | unknown protein [ORF2] | Unknown | N/A |
| slr9203 | unknown protein [ORF1] | Unknown | N/A |
| sml0001 | photosystem II reaction center PsbI protein | Photosynthesis and respiration | Photosystem II |
| sml0002 | photosystem II PsbX protein | Photosynthesis and respiration | Photosystem II |
| sml0003 | photosystem II reaction center M protein | Photosynthesis and respiration | Photosystem II |
| sml0004 | cytochrome b6f complex subunit VIII | Photosynthesis and respiration | Cytochrome b6/f complex |
| sml0005 | photosystem II PsbK protein | Photosynthesis and respiration | Photosystem II |
| sml0007 | photosystem II protein Y | Photosynthesis and respiration | Photosystem II |
| sml0008 | photosystem I subunit IX | Photosynthesis and respiration | Photosystem I |
| sml0009 | similar to virulence-associated protein VapC | Cellular processes | Transformation |
| sml0010 | putative transposase: | Other categories | Transposon-related functions |
| sml0011 | hypothetical protein | Hypothetical | N/A |
| sml0012 | hypothetical protein | Hypothetical | N/A |
| sml0013 | hypothetical protein | Hypothetical | N/A |
| smr0001 | photosystem II PsbT protein | Photosynthesis and respiration | Photosystem II |
| smr0002 | putative transposase [ISY100v: 3095975 - 3096319, join 3097194 - 3097362, join 3098314 - 3098743] | Other categories | Transposon-related functions |
| smr0003 | cytochrome b6f complex subunit PetM | Photosynthesis and respiration | Cytochrome b6/f complex |
| smr0004 | photosystem I subunit VIII | Photosynthesis and respiration | Photosystem I |
| smr0005 | photosystem I subunit XII | Photosynthesis and respiration | Photosystem I |
| smr0006 | cytochrome b559 b subunit | Photosynthesis and respiration | Photosystem II |
| smr0007 | photosystem II PsbL protein | Photosynthesis and respiration | Photosystem II |
| smr0008 | photosystem II PsbJ protein | Photosynthesis and respiration | Photosystem II |
| smr0009 | photosystem II PsbN protein | Photosynthesis and respiration | Photosystem II |
| smr0010 | cytochrome b6f complex subunit 5 | Photosynthesis and respiration | Cytochrome b6/f complex |
| smr0011 | 50S ribosomal protein L34 | Translation | Ribosomal proteins |
| smr0012 | putative transposase [ISY523j(partial copy): 106904 - 107226] | Other categories | Transposon-related functions |
| smr0013 | hypothetical protein | Hypothetical | N/A |
| smr0014 | hypothetical protein | Hypothetical | N/A |
| smr0015 | hypothetical protein | Hypothetical | N/A |
| ssl0090 | hypothetical protein | Hypothetical | N/A |
| ssl0105 | hypothetical protein | Hypothetical | N/A |
| ssl0109 | unknown protein | Unknown | N/A |
| ssl0172 | putative transposase [ISY391c: 2997600 - 2998989] | Other categories | Transposon-related functions |
| ssl0241 | hypothetical protein | Hypothetical | N/A |
| ssl0242 | hypothetical protein | Hypothetical | N/A |
| ssl0258 | hypothetical protein | Hypothetical | N/A |
| ssl0259 | hypothetical protein | Hypothetical | N/A |
| ssl0294 | hypothetical protein | Hypothetical | N/A |
| ssl0296 | putative transposase [ISY523p: 2328180 - 2329050] | Other categories | Transposon-related functions |
| ssl0312 | hypothetical protein | Hypothetical | N/A |
| ssl0318 | unknown protein | Unknown | N/A |
| ssl0323 | unknown protein | Unknown | N/A |
| ssl0331 | hypothetical protein | Hypothetical | N/A |
| ssl0350 | unknown protein | Unknown | N/A |
| ssl0353 | hypothetical protein | Hypothetical | N/A |
| ssl0385 | hypothetical protein | Hypothetical | N/A |
| ssl0410 | unknown protein | Unknown | N/A |
| ssl0426 | putative transposase [ISY100t(partial copy): 141097 - 141410] | Other categories | Transposon-related functions |
| ssl0431 | unknown protein | Unknown | N/A |
| ssl0438 | similar to 50S ribosomal protein L12 | Translation | Ribosomal proteins |
| ssl0452 | phycobilisome degradation protein NblA | Photosynthesis and respiration | Phycobilisome |
| ssl0453 | phycobilisome degradation protein NblA | Photosynthesis and respiration | Phycobilisome |
| ssl0461 | hypothetical protein | Hypothetical | N/A |
| ssl0467 | unknown protein | Unknown | N/A |
| ssl0483 | hypothetical protein | Hypothetical | N/A |
| ssl0511 | hypothetical protein | Hypothetical | N/A |
| ssl0546 | septum site-determining protein MinE | Cellular processes | Cell division |
| ssl0564 | transcriptional regulator | Regulatory functions | N/A |
| ssl0601 | 30S ribosomal protein S21 | Translation | Ribosomal proteins |
| ssl0606 | unknown protein | Unknown | N/A |
| ssl0738 | unknown protein | Unknown | N/A |
| ssl0739 | hypothetical protein | Hypothetical | N/A |
| ssl0750 | unknown protein | Unknown | N/A |
| ssl0769 | putative transposase: | Other categories | Transposon-related functions |
| ssl0832 | hypothetical protein | Hypothetical | N/A |
| ssl0900 | hypothetical protein | Hypothetical | N/A |
| ssl1004 | hypothetical protein | Hypothetical | N/A |
| ssl1045 | hypothetical protein | Hypothetical | N/A |
| ssl1046 | hypothetical protein | Hypothetical | N/A |
| ssl1047 | hypothetical protein | Hypothetical | N/A |
| ssl1255 | hypothetical protein | Hypothetical | N/A |
| ssl1263 | hypothetical protein | Hypothetical | N/A |
| ssl1277 | putative transposase: | Other categories | Transposon-related functions |
| ssl1300 | hypothetical protein | Hypothetical | N/A |
| ssl1326 | unknown protein | Unknown | N/A |
| ssl1328 | hypothetical protein | Hypothetical | N/A |
| ssl1376 | hypothetical protein | Hypothetical | N/A |
| ssl1377 | hypothetical protein | Hypothetical | N/A |
| ssl1378 | hypothetical protein | Hypothetical | N/A |
| ssl1417 | hypothetical protein YCF33 | Hypothetical | N/A |
| ssl1426 | 50S ribosomal protein L35 | Translation | Ribosomal proteins |
| ssl1464 | unknown protein | Unknown | N/A |
| ssl1493 | unknown protein | Unknown | N/A |
| ssl1498 | hypothetical protein | Hypothetical | N/A |
| ssl1507 | putative transposase [ISY508a: 1710788 - 1711753] | Other categories | Transposon-related functions |
| ssl1520 | unknown protein | Unknown | N/A |
| ssl1533 | unknown protein | Unknown | N/A |
| ssl1552 | unknown protein | Unknown | N/A |
| ssl1577 | hypothetical protein | Hypothetical | N/A |
| ssl1633 | high light-inducible polypeptide HliC, CAB/ELIP/HLIP superfamily | Hypothetical | N/A |
| ssl1707 | hypothetical protein | Hypothetical | N/A |
| ssl1762 | hypothetical protein | Hypothetical | N/A |
| ssl1784 | 30S ribosomal protein S15 | Translation | Ribosomal proteins |
| ssl1792 | hypothetical protein | Hypothetical | N/A |
| ssl1807 | hypothetical protein | Hypothetical | N/A |
| ssl1911 | glutamine synthetase inactivating factor IF7 | Hypothetical | N/A |
| ssl1918 | hypothetical protein | Hypothetical | N/A |
| ssl1920 | putative transposase [ISY523l(partial copy): 520871 - 521420] | Other categories | Transposon-related functions |
| ssl1922 | putative transposase [ISY523l(partial copy): 520871 - 521420] | Other categories | Transposon-related functions |
| ssl1923 | hypothetical protein | Hypothetical | N/A |
| ssl2065 | unknown protein | Unknown | N/A |
| ssl2069 | hypothetical protein | Hypothetical | N/A |
| ssl2100 | unknown protein | Unknown | N/A |
| ssl2138 | unknown protein | Unknown | N/A |
| ssl2148 | hypothetical protein | Hypothetical | N/A |
| ssl2153 | probable ribose phosphate isomerase B | Energy metabolism | Sugars |
| ssl2162 | unknown protein | Unknown | N/A |
| ssl2233 | 30S ribosomal protein S20 | Translation | Ribosomal proteins |
| ssl2245 | unknown protein | Unknown | N/A |
| ssl2250 | bacterioferritin-associated ferredoxin | Other categories | Drug and analog sensitivity |
| ssl2380 | unknown protein | Unknown | N/A |
| ssl2384 | unknown protein | Unknown | N/A |
| ssl2420 | unknown protein | Unknown | N/A |
| ssl2471 | hypothetical protein | Hypothetical | N/A |
| ssl2502 | unknown protein | Unknown | N/A |
| ssl2507 | unknown protein | Unknown | N/A |
| ssl2542 | high light-inducible polypeptide HliA, CAB/ELIP/HLIP superfamily | Other categories | Adaptations and atypical conditions |
| ssl2559 | ferredoxin | Photosynthesis and respiration | Soluble electron carriers |
| ssl2615 | ATP synthase C chain of CF(0) | Photosynthesis and respiration | ATP synthase |
| ssl2648 | hypothetical protein | Hypothetical | N/A |
| ssl2653 | unknown protein | Unknown | N/A |
| ssl2667 | an assembly factor for iron-sulfur culsters | Amino acid biosynthesis | Glutamate family / Nitrogen assimilation |
| ssl2717 | hypothetical protein | Hypothetical | N/A |
| ssl2733 | hypothetical protein | Hypothetical | N/A |
| ssl2749 | hypothetical protein | Hypothetical | N/A |
| ssl2789 | similar to resolvase: | Other categories | Transposon-related functions |
| ssl2807 | hypothetical protein | Hypothetical | N/A |
| ssl2814 | unknown protein | Unknown | N/A |
| ssl2823 | hypothetical protein | Hypothetical | N/A |
| ssl2891 | unknown protein | Unknown | N/A |
| ssl2920 | hypothetical protein | Hypothetical | N/A |
| ssl2921 | hypothetical protein | Hypothetical | N/A |
| ssl2922 | similar to virulence-associated protein VapB | Cellular processes | Transformation |
| ssl2923 | similar to virulence-associated protein VapC | Cellular processes | Transformation |
| ssl2971 | hypothetical protein | Hypothetical | N/A |
| ssl2996 | unknown protein | Unknown | N/A |
| ssl3044 | probable ferredoxin | Photosynthesis and respiration | Soluble electron carriers |
| ssl3076 | unknown protein | Unknown | N/A |
| ssl3127 | similar to permease protein of ABC transporter | Transport and binding proteins | N/A |
| ssl3142 | unknown protein | Unknown | N/A |
| ssl3177 | hypothetical protein | Hypothetical | N/A |
| ssl3222 | unknown protein | Unknown | N/A |
| ssl3291 | hypothetical protein | Hypothetical | N/A |
| ssl3297 | hypothetical protein | Hypothetical | N/A |
| ssl3335 | preprotein translocase SecE subunit | Cellular processes | Protein and peptide secretion |
| ssl3342 | hypothetical protein | Hypothetical | N/A |
| ssl3379 | hypothetical protein | Hypothetical | N/A |
| ssl3382 | hypothetical protein | Hypothetical | N/A |
| ssl3383 | unknown protein | Unknown | N/A |
| ssl3389 | hypothetical protein | Hypothetical | N/A |
| ssl3410 | unknown protein | Unknown | N/A |
| ssl3432 | 30S ribosomal protein S19 | Translation | Ribosomal proteins |
| ssl3437 | 30S ribosomal protein S17 | Translation | Ribosomal proteins |
| ssl3446 | hypothetical protein | Hypothetical | N/A |
| ssl3451 | hypothetical protein | Hypothetical | N/A |
| ssl3549 | hypothetical protein | Hypothetical | N/A |
| ssl3573 | hypothetical protein | Hypothetical | N/A |
| ssl3580 | putative hydrogenase expression/formation protein HypC | Other categories | Hydrogenase |
| ssl3615 | unknown protein | Unknown | N/A |
| ssl3649 | putative transposase [ISY120d(partial copy): 604956 - 605288] | Other categories | Transposon-related functions |
| ssl3692 | hypothetical protein | Hypothetical | N/A |
| ssl3712 | hypothetical protein | Hypothetical | N/A |
| ssl3719 | hypothetical protein | Hypothetical | N/A |
| ssl3769 | unknown protein | Unknown | N/A |
| ssl3803 | hypothetical protein | Hypothetical | N/A |
| ssl3829 | hypothetical protein | Hypothetical | N/A |
| ssl5001 | unknown protein | Unknown | N/A |
| ssl5007 | unknown protein | Unknown | N/A |
| ssl5008 | unknown protein | Unknown | N/A |
| ssl5015 | unknown protein | Unknown | N/A |
| ssl5025 | hypothetical protein | Hypothetical | N/A |
| ssl5027 | hypothetical protein | Hypothetical | N/A |
| ssl5031 | hypothetical protein | Hypothetical | N/A |
| ssl5039 | unknown protein | Unknown | N/A |
| ssl5045 | unknown protein | Unknown | N/A |
| ssl5064 | hypothetical protein | Hypothetical | N/A |
| ssl5065 | unknown protein | Unknown | N/A |
| ssl5068 | unknown protein | Unknown | N/A |
| ssl5070 | unknown protein | Unknown | N/A |
| ssl5091 | unknown protein | Unknown | N/A |
| ssl5095 | hypothetical protein | Hypothetical | N/A |
| ssl5096 | unknown protein | Unknown | N/A |
| ssl5098 | unknown protein | Unknown | N/A |
| ssl5099 | hypothetical protein | Hypothetical | N/A |
| ssl5100 | hypothetical protein | Hypothetical | N/A |
| ssl5103 | unknown protein | Unknown | N/A |
| ssl5108 | unknown protein | Unknown | N/A |
| ssl5113 | unknown protein | Unknown | N/A |
| ssl5114 | unknown protein | Unknown | N/A |
| ssl5125 | unknown protein | Unknown | N/A |
| ssl5129 | hypothetical protein | Hypothetical | N/A |
| ssl6018 | unknown protein | Unknown | N/A |
| ssl6023 | unknown protein | Unknown | N/A |
| ssl6035 | unknown protein | Unknown | N/A |
| ssl6061 | unknown protein | Unknown | N/A |
| ssl6077 | unknown protein | Unknown | N/A |
| ssl6082 | unknown protein | Unknown | N/A |
| ssl6092 | unknown protein | Unknown | N/A |
| ssl7004 | probable plasmid stability protein | Other categories | Other |
| ssl7007 | hypothetical protein | Hypothetical | N/A |
| ssl7019 | unknown protein | Unknown | N/A |
| ssl7020 | unknown protein | Unknown | N/A |
| ssl7021 | unknown protein | Unknown | N/A |
| ssl7022 | unknown protein | Unknown | N/A |
| ssl7038 | hypothetical protein | Hypothetical | N/A |
| ssl7039 | hypothetical protein | Hypothetical | N/A |
| ssl7042 | hypothetical protein | Hypothetical | N/A |
| ssl7045 | unknown protein | Unknown | N/A |
| ssl7046 | hypothetical protein | Hypothetical | N/A |
| ssl7048 | hypothetical protein | Hypothetical | N/A |
| ssl7051 | unknown protein | Unknown | N/A |
| ssl7053 | hypothetical protein | Hypothetical | N/A |
| ssl7074 | hypothetical protein | Hypothetical | N/A |
| ssl8003 | unknown protein | Unknown | N/A |
| ssl8005 | hypothetical protein | Hypothetical | N/A |
| ssl8008 | hypothetical protein | Hypothetical | N/A |
| ssl8010 | type I restriction-modification system, M subunit | DNA replication, restriction, modification, recombination, and repair | N/A |
| ssl8024 | unknown protein | Unknown | N/A |
| ssl8028 | hypothetical protein | Hypothetical | N/A |
| ssl8039 | unknown protein | Unknown | N/A |
| ssl8041 | transposase: | Other categories | Transposon-related functions |
| ssl9001 | unknown protein [ORF-F] | Unknown | N/A |
| ssr0102 | hypothetical protein YCF40 | Hypothetical | N/A |
| ssr0109 | hypothetical protein | Hypothetical | N/A |
| ssr0256 | putative transposase [ISY523n: 2210271 - 2211142] | Other categories | Transposon-related functions |
| ssr0332 | hypothetical protein | Hypothetical | N/A |
| ssr0335 | unknown protein | Unknown | N/A |
| ssr0336 | hypothetical protein | Hypothetical | N/A |
| ssr0349 | hypothetical protein | Hypothetical | N/A |
| ssr0390 | photosystem I reaction center subunit X | Photosynthesis and respiration | Photosystem I |
| ssr0511 | unknown protein | Unknown | N/A |
| ssr0515 | hypothetical protein | Hypothetical | N/A |
| ssr0536 | unknown protein | Unknown | N/A |
| ssr0550 | hypothetical protein | Hypothetical | N/A |
| ssr0657 | hypothetical protein | Hypothetical | N/A |
| ssr0663 | hypothetical protein | Hypothetical | N/A |
| ssr0680 | unknown protein | Unknown | N/A |
| ssr0692 | hypothetical protein | Hypothetical | N/A |
| ssr0693 | unknown protein | Unknown | N/A |
| ssr0706 | unknown protein | Unknown | N/A |
| ssr0755 | hypothetical protein | Hypothetical | N/A |
| ssr0756 | hypothetical protein | Hypothetical | N/A |
| ssr0757 | hypothetical protein | Hypothetical | N/A |
| ssr0759 | unknown protein | Unknown | N/A |
| ssr0761 | hypothetical protein | Hypothetical | N/A |
| ssr0817 | putative transposase [ISY352g: 3511668 - 3512290, join 3513238 - 3514051] | Other categories | Transposon-related functions |
| ssr0854 | hypothetical protein | Hypothetical | N/A |
| ssr0871 | putative transposase [ISY352e: 2921301 - 2921595, join 3108631 - 3109754] | Other categories | Transposon-related functions |
| ssr1038 | unknown protein | Unknown | N/A |
| ssr1041 | hypothetical protein | Hypothetical | N/A |
| ssr1049 | unknown protein | Unknown | N/A |
| ssr1114 | hypothetical protein | Hypothetical | N/A |
| ssr1155 | hypothetical protein | Hypothetical | N/A |
| ssr1169 | stress induced hydrophobic peptide homolog | Other categories | Adaptations and atypical conditions |
| ssr1175 | putative transposase [ISY100v: 3095975 - 3096319, join 3097194 - 3097362, join 3098314 - 3098743] | Other categories | Transposon-related functions |
| ssr1176 | putative transposase [ISY100v: 3095975 - 3096319, join 3097194 - 3097362, join 3098314 - 3098743] | Other categories | Transposon-related functions |
| ssr1238 | hypothetical protein | Hypothetical | N/A |
| ssr1251 | hypothetical protein | Hypothetical | N/A |
| ssr1256 | hypothetical protein | Hypothetical | N/A |
| ssr1258 | hypothetical protein | Hypothetical | N/A |
| ssr1260 | hypothetical protein | Hypothetical | N/A |
| ssr1274 | unknown protein | Unknown | N/A |
| ssr1375 | hypothetical protein | Hypothetical | N/A |
| ssr1386 | NADH dehydrogenase subunit NdhL | Photosynthesis and respiration | NADH dehydrogenase |
| ssr1391 | hypothetical protein | Hypothetical | N/A |
| ssr1398 | 50S ribosomal protein L33 | Translation | Ribosomal proteins |
| ssr1407 | hypothetical protein | Hypothetical | N/A |
| ssr1425 | hypothetical protein YCF34 | Hypothetical | N/A |
| ssr1473 | hypothetical protein | Hypothetical | N/A |
| ssr1499 | hypothetical protein | Hypothetical | N/A |
| ssr1513 | hypothetical protein | Hypothetical | N/A |
| ssr1527 | probable molybdopterin [MPT] converting factor, subunit 1 | Biosynthesis of cofactors, prosthetic groups, and carriers | Molybdopterin |
| ssr1552 | hypothetical protein | Hypothetical | N/A |
| ssr1558 | hypothetical protein | Hypothetical | N/A |
| ssr1562 | hypothetical protein | Hypothetical | N/A |
| ssr1604 | 50S ribosomal protein L28 | Translation | Ribosomal proteins |
| ssr1720 | similar to tyrosyl tRNA synthetase | Translation | Aminoacyl tRNA synthetases and tRNA modification |
| ssr1736 | 50S ribosomal protein L32 | Translation | Ribosomal proteins |
| ssr1765 | hypothetical protein | Hypothetical | N/A |
| ssr1766 | hypothetical protein | Hypothetical | N/A |
| ssr1768 | unknown protein | Unknown | N/A |
| ssr1789 | CAB/ELIP/HLIP-related protein HliD | Other categories | Adaptations and atypical conditions |
| ssr1880 | hypothetical protein | Hypothetical | N/A |
| ssr1951 | hypothetical protein | Hypothetical | N/A |
| ssr1966 | hypothetical protein | Hypothetical | N/A |
| ssr2009 | hypothetical protein | Hypothetical | N/A |
| ssr2016 | hypothetical protein | Hypothetical | N/A |
| ssr2047 | hypothetical protein | Hypothetical | N/A |
| ssr2049 | unknown protein | Unknown | N/A |
| ssr2060 | unknown protein | Unknown | N/A |
| ssr2062 | hypothetical protein | Hypothetical | N/A |
| ssr2066 | hypothetical protein | Hypothetical | N/A |
| ssr2067 | hypothetical protein | Hypothetical | N/A |
| ssr2078 | putative transposase [ISY802b(partial copy): 1384736 - 1385513] | Other categories | Transposon-related functions |
| ssr2087 | hypothetical protein | Hypothetical | N/A |
| ssr2130 | hypothetical protein | Hypothetical | N/A |
| ssr2142 | hypothetical protein YCF19 | Hypothetical | N/A |
| ssr2153 | unknown protein | Unknown | N/A |
| ssr2194 | unknown protein | Unknown | N/A |
| ssr2201 | unknown protein | Unknown | N/A |
| ssr2227 | putative transposase: | Other categories | Transposon-related functions |
| ssr2254 | unknown protein | Unknown | N/A |
| ssr2317 | unknown protein | Unknown | N/A |
| ssr2318 | unknown protein | Unknown | N/A |
| ssr2333 | unknown protein | Unknown | N/A |
| ssr2340 | hypothetical protein | Hypothetical | N/A |
| ssr2377 | hypothetical protein | Hypothetical | N/A |
| ssr2406 | unknown protein | Unknown | N/A |
| ssr2439 | hypothetical protein | Hypothetical | N/A |
| ssr2549 | unknown protein | Unknown | N/A |
| ssr2551 | hypothetical protein | Hypothetical | N/A |
| ssr2554 | hypothetical protein | Hypothetical | N/A |
| ssr2595 | high light-inducible polypeptide HliB, CAB/ELIP/HLIP superfamily | Other categories | Adaptations and atypical conditions |
| ssr2611 | hypothetical protein | Hypothetical | N/A |
| ssr2615 | hypothetical protein | Hypothetical | N/A |
| ssr2699 | putative transposase [ISY523k(partial copy): 473025 - 473752] | Other categories | Transposon-related functions |
| ssr2708 | hypothetical protein | Hypothetical | N/A |
| ssr2710 | hypothetical protein | Hypothetical | N/A |
| ssr2711 | hypothetical protein | Hypothetical | N/A |
| ssr2723 | hypothetical protein | Hypothetical | N/A |
| ssr2754 | hypothetical protein | Hypothetical | N/A |
| ssr2781 | hypothetical protein | Hypothetical | N/A |
| ssr2784 | antitoxin ChpI homolog | Cellular processes | Detoxification |
| ssr2802 | hypothetical protein | Hypothetical | N/A |
| ssr2803 | hypothetical protein | Hypothetical | N/A |
| ssr2806 | hypothetical protein | Hypothetical | N/A |
| ssr2843 | hypothetical protein | Hypothetical | N/A |
| ssr2848 | unknown protein | Unknown | N/A |
| ssr2898 | putative transposase [ISY523m(partial copy): 1483390 - 1484062] | Other categories | Transposon-related functions |
| ssr2899 | putative transposase [ISY523m(partial copy): 1483390 - 1484062] | Other categories | Transposon-related functions |
| ssr2912 | unknown protein | Unknown | N/A |
| ssr2962 | hypothetical protein | Hypothetical | N/A |
| ssr2972 | unknown protein | Unknown | N/A |
| ssr2975 | unknown protein | Unknown | N/A |
| ssr3000 | hypothetical protein | Hypothetical | N/A |
| ssr3129 | unknown protein | Unknown | N/A |
| ssr3154 | hypothetical protein | Hypothetical | N/A |
| ssr3159 | unknown protein | Unknown | N/A |
| ssr3184 | 4Fe-4S type iron-sulfur protein | Photosynthesis and respiration | Soluble electron carriers |
| ssr3188 | hypothetical protein | Hypothetical | N/A |
| ssr3189 | hypothetical protein | Hypothetical | N/A |
| ssr3300 | unknown protein | Unknown | N/A |
| ssr3304 | hypothetical protein | Hypothetical | N/A |
| ssr3307 | preprotein translocase SecG subunit | Cellular processes | Protein and peptide secretion |
| ssr3341 | hypothetical protein | Hypothetical | N/A |
| ssr3402 | unknown protein | Unknown | N/A |
| ssr3409 | hypothetical protein | Hypothetical | N/A |
| ssr3410 | hypothetical protein | Hypothetical | N/A |
| ssr3452 | putative transposase [ISY352a(partial copy): 572672 - 572905] | Other categories | Transposon-related functions |
| ssr3465 | unknown protein | Unknown | N/A |
| ssr3467 | unknown protein | Unknown | N/A |
| ssr3550 | hypothetical protein | Hypothetical | N/A |
| ssr3570 | unknown protein | Unknown | N/A |
| ssr3571 | hypothetical protein | Hypothetical | N/A |
| ssr3572 | hypothetical protein | Hypothetical | N/A |
| ssr3588 | hypothetical protein | Hypothetical | N/A |
| ssr3589 | hypothetical protein | Hypothetical | N/A |
| ssr5009 | unknown protein | Unknown | N/A |
| ssr5011 | hypothetical protein | Hypothetical | N/A |
| ssr5019 | hypothetical protein | Hypothetical | N/A |
| ssr5020 | hypothetical protein | Hypothetical | N/A |
| ssr5074 | unknown protein | Unknown | N/A |
| ssr5092 | hypothetical protein | Hypothetical | N/A |
| ssr5106 | hypothetical protein | Hypothetical | N/A |
| ssr5117 | hypothetical protein | Hypothetical | N/A |
| ssr5120 | unknown protein | Unknown | N/A |
| ssr5121 | hypothetical protein | Hypothetical | N/A |
| ssr6002 | unknown protein | Unknown | N/A |
| ssr6003 | unknown protein | Unknown | N/A |
| ssr6019 | unknown protein | Unknown | N/A |
| ssr6020 | unknown protein | Unknown | N/A |
| ssr6024 | unknown protein | Unknown | N/A |
| ssr6026 | unknown protein | Unknown | N/A |
| ssr6027 | unknown protein | Unknown | N/A |
| ssr6030 | unknown protein | Unknown | N/A |
| ssr6032 | hypothetical protein | Hypothetical | N/A |
| ssr6046 | hypothetical protein | Hypothetical | N/A |
| ssr6048 | unknown protein | Unknown | N/A |
| ssr6062 | unknown protein | Unknown | N/A |
| ssr6078 | unknown protein | Unknown | N/A |
| ssr6079 | unknown protein | Unknown | N/A |
| ssr6083 | unknown protein | Unknown | N/A |
| ssr6085 | unknown protein | Unknown | N/A |
| ssr6086 | unknown protein | Unknown | N/A |
| ssr6089 | unknown protein | Unknown | N/A |
| ssr6099 | unknown protein | Unknown | N/A |
| ssr7017 | hypothetical protein | Hypothetical | N/A |
| ssr7018 | unknown protein | Unknown | N/A |
| ssr7035 | unknown protein | Unknown | N/A |
| ssr7036 | unknown protein | Unknown | N/A |
| ssr7040 | probable cell growth regulatory protein | Other categories | Other |
| ssr7072 | hypothetical protein | Hypothetical | N/A |
| ssr7079 | unknown protein | Unknown | N/A |
| ssr7084 | unknown protein | Unknown | N/A |
| ssr7093 | hypothetical protein | Hypothetical | N/A |
| ssr8013 | hypothetical protein | Hypothetical | N/A |
| ssr8047 | unknown protein | Unknown | N/A |
| ssr9004 | hypothetical protein | Hypothetical | N/A |
| ssr9005 | unknown protein [ORF-D] | Unknown | N/A |
| ssr9202 | unknown protein [ORF3] | Unknown | N/A |
