# Supplementary figures and images for: Identification and bioinformatic analysis of the membrane proteins of synechocystis sp. PCC 6803
Source: Proteome Sci. 2009 Mar 25;7:11. doi: 10.1186/1477-5956-7-11 (PMC2666656; doi:10.1186/1477-5956-7-11)

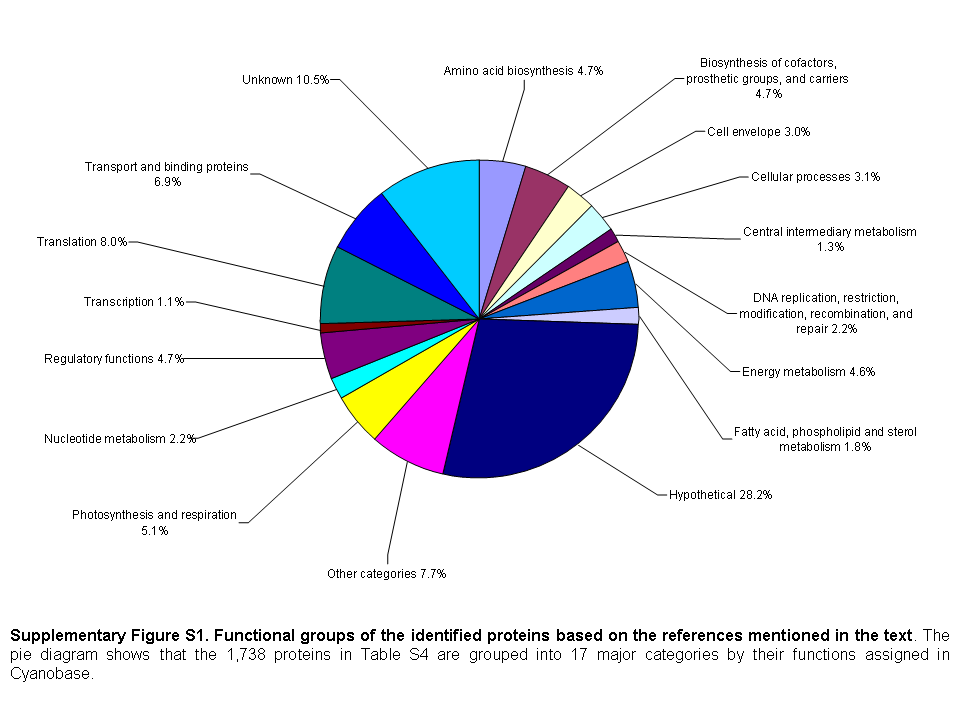

Supplement: Additional file 6 — Functional groups of the identified proteins based on the references mentioned in the text. Additional file 6 is a pie diagram shows that the 1,738 proteins in additional file 4 are grouped into 17 major categories by their functions assigned in Cyanobase. [file 1477-5956-7-11-S6.tiff]

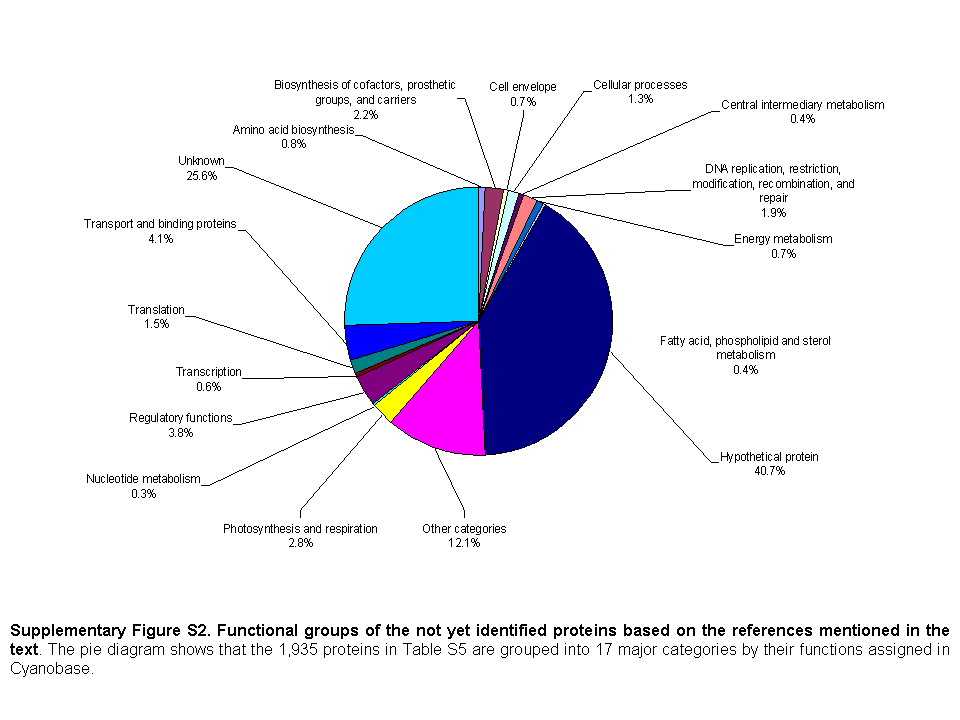

Supplement: Additional file 7 — Functional groups of the not yet identified proteins based on the references mentioned in the text. Additional file 7 is a pie diagram shows that the 1,935 proteins in additional file 5 are grouped into 17 major categories by their functions assigned in Cyanobase. [file 1477-5956-7-11-S7.tiff]
